# Subcellular Localization Signals of bHLH-PAS Proteins: Their Significance, Current State of Knowledge and Future Perspectives

**DOI:** 10.3390/ijms20194746

**Published:** 2019-09-24

**Authors:** Beata Greb-Markiewicz, Marta Kolonko

**Affiliations:** Department of Biochemistry, Faculty of Chemistry, Wrocław University of Science and Technology, Wybrzeże Wyspiańskiego 27, 50-370 Wrocław, Poland; marta.kolonko@pwr.edu.pl

**Keywords:** ARNT, BMAL, bHLH-PAS, CLOCK, CYCLE, GCE, HIF, MET, NPAS, NLS, NES, nucleocytoplasmic shuttling, SIM, SIMA, TANGO

## Abstract

The bHLH-PAS (basic helix-loop-helix/ Period-ARNT-Single minded) proteins are a family of transcriptional regulators commonly occurring in living organisms. bHLH-PAS members act as intracellular and extracellular “signals” sensors, initiating response to endo- and exogenous signals, including toxins, redox potential, and light. The activity of these proteins as transcription factors depends on nucleocytoplasmic shuttling: the signal received in the cytoplasm has to be transduced, via translocation, to the nucleus. It leads to the activation of transcription of particular genes and determines the cell response to different stimuli. In this review, we aim to present the current state of knowledge concerning signals that affect shuttling of bHLH-PAS transcription factors. We summarize experimentally verified and published nuclear localization signals/nuclear export signals (NLSs/NESs) in the context of performed in silico predictions. We have used most of the available NLS/NES predictors. Importantly, all our results confirm the existence of a complex system responsible for protein localization regulation that involves many localization signals, which activity has to be precisely controlled. We conclude that the current stage of knowledge in this area is still not complete and for most of bHLH-PAS proteins an experimental verification of the activity of further NLS/NES is needed.

## 1. Introduction

The bHLH-PAS (basic helix-loop-helix/ Period-ARNT-Single minded) proteins are a family of transcriptional regulators commonly occurring in living organisms [1,2]. They play a significant role in essential physiological and developmental processes [2] and a number of them participates in adaptive responses to generalized and cellular stress [3]. bHLH-PAS family members act as intracellular and extracellular “signals” sensors, initiating primary response to endogenous compounds, foreign chemicals, gas molecules, redox potential, light, and others [4].

**Figure 1:**
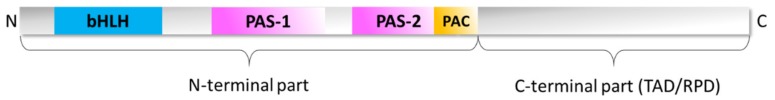
Schematic representation of the basic helix-loop-helix/ Period-ARNT-Single minded (bHLH-PAS) protein domain structure. The N-terminal part is characterized by the presence of defined domains: bHLH (blue), PAS-1 and PAS-2 (rose) associated with PAC (C-terminal to PAS domain) motif (yellow). The C-terminal part is highly variable and often contains transactivation/repression domains (TAD/RPD). Based on [2].

Despite the fact that bHLH-PAS proteins perform a high diversity of function, their structural properties have been well-conserved during evolution (Figure 1) [1]. The N-terminal part comprises two domains: bHLH and PAS. The bHLH domain can be divided into two specific fragments: the basic region responsible for DNA binding, and the HLH region, which takes part in protein dimerization [5]. The followed PAS domain comprises two structurally conserved regions named PAS-1 and PAS-2 [6], separated by a poorly conserved link [1]. The PAS-1 takes part in a dimerization partner selection and ensures the specificity of target gene activation [2]. The PAS-2 is typically responsible for ligand binding and sensing diverse exogenous and endogenous signals, which enable protein activity regulation [2,7]. PAS-2 is often associated with PAC (C-terminal to PAS domain), which is proposed to contribute to the PAS domain appropriate folding. The division between the PAS and PAC domains is caused by major sequence differences in the region connecting these two motifs [8]. Contrary to conserved domains, the C-terminal part of bHLH-PAS proteins sequence is significantly variable [2] and contains transcription activation/repression domains (TAD/RPD) [9,10].

bHLH-PAS proteins, like many other transcription factors (TFs), dimerize with other family members to create functional heterodimer acting as a functional complex regulating the expression of genes under its control [4]. Consequently, most of bHLH–PAS proteins can be divided into two classes: class I proteins whose expression is specifically regulated by diverse physiological states and/or environmental signals [11], and class II proteins expressed in a constitutive way and serving as heterodimerization partners for class I members [4].

For a number of TFs regulating gene expression in response to extracellular signals, translocation from the cytoplasm to the nucleus is an important event, enabling TFs to recruit coactivators [12,13]. As shown, steroid/nuclear receptors continuously shuttle between the cytoplasm and the nucleus and their localization at any time is a consequence of the fine balance between the operational strength of the sequences for the nuclear localization signal (NLS) and the nuclear export signal (NES) [14]. The nuclear transport is usually mediated by the family of transport receptors known as karyopherins: importins, responsible for nuclear import, and exportins, like exportin-1 (CRM1, Chromosome region maintenance 1 protein homolog), responsible for nuclear export [15]. Karyopherins recognize specific NLS/NES signals presented at cargo protein to create the transporting complex [15]. The best-described transport signal responsible for nuclear import is the classical NLS (cNLS) consisting of monopartite or bipartite motifs rich in basic amino acid residues [16]. The most popular NES is a non-conserved motif-containing hydrophobic residues including leucine residues repeats [15]. Known inhibitor of protein export dependent on exportin-1 and often used for studies of NES activity is Leptomycin B (LMB) [17,18].

bHLH-PAS proteins are an important class of TFs which activity depends on nucleocytoplasmic shuttling. To perform their function as sensors, indispensable is receiving the signal in cytoplasm and its transduction, by translocation, to the nucleus. It leads to the activation of particular genes transcription. Previously, we have performed systematic research of NLS and NES motifs in *Drosophila melonagaster* Methoprene tolerant protein (MET) [19] and its paralog—germ cell-expressed (GCE) protein [20]. Then, we carried out similar analyzes for mammalian NPAS4 protein [21]. We have determined an interesting pattern of overlaying signals with opposing activity in bHLH and PAC domains as well as in C-termini. Our results were the first, suggesting the presence of multiple localization signals regulating TF shuttling and their complex localization pattern. Now, we ask a question if it is more general mechanism of bHLH-PAS proteins localization regulation and how precise and systematic studies in this area are reported to date.

To answer the question, we performed bioinformatic analysis allowing to predict NLS and NES signals. We have studied human representatives of bHLH/PAS class I: AHR (Aryl hydrocarbon Receptor), HIF-1α (Hypoxia Inducible Factor), HIF-2α and HIF-3α, SIM1 (Single-minded homolog) and SIM2, NPAS1-4 (Neuronal PAS domain-containing protein), CLOCK, and representatives of class II: ARNT (Aryl Hydrocarbon Nuclear Translocator), ARNT2, ARNTL1 (Aryl hydrocarbon receptor nuclear translocator-like protein 1, BMAL1, ARNT3), ARNTL2 (BMAL2, ARNT4). Additionally, we analyzed *Drosophila* class I proteins: MET, GCE, SIMA (similar) and class II representant: TANGO, being a homolog of mammalian ARNT and CYCLE (homolog of mammalian BMAL1). We used cNLS Mapper [22] (http://nls-mapper.iab.keio.ac.jp/cgi-bin/NLS_Mapper_y.cgi), NLStradamus [23] (http://www.moseslab.csb.utoronto.ca/NLStradamus/), PSORTII [24] (http://www.psort.org/), NucPred (http://www.sbc.su.se/~maccallr/nucpred/) and SeqNLS [25] (http://mleg.cse.sc.edu/seqNLS/) servers for NLS predictions, while NetNES [26] (http://www.cbs.dtu.dk/services/NetNES/), NESFinder0.2 [27] (http://research.nki.nl/fornerodlab/NES-Finder.htm) and LocNES [28] (http://prodata.swmed.edu/LocNES/LocNES.php) for NES predictions. Additionally, we used The Eukaryotic linear motif resource ELM for both putative NLS and NES searching [29,30,31] (http://elm.eu.org/).

In this review, we present results of performed predictions in the context of published data concerning NLSs/NESs taking part in regulation of bHLH-PAS TFs shuttling. As most of the published research and predictions were performed previously only for selected fragments of these proteins, the current state of knowledge in this area is still not complete.

## 2. Regulation of the Subcellular Localization of Class I bHLH-PAS Transcription Factors

### 2.1. AHR Localization Regulation

AHR is the only known bHLH-PAS cytoplasmic sensor activated by small ligands. It is involved in dioxin and related environmental pollutants metabolism [32]. It was shown [33] that non-ligated AHR creates an inactive heterodimer with Hsp90 chaperone protein and is present in the cytoplasm. Hsp90 prevents AHR proteolysis and maintains the receptor in a conformation susceptible to ligand binding [34]. Ligand binding enforces translocation of AHR to nucleus, where AHR dimerizes with its partner bHLH-PAS protein: ARNT, to create a functional complex [35]. The AHR/ARNT heterodimer regulates genes encoding xenobiotic metabolizing enzymes and mediates the severe toxicity, comprising a wasting syndrome, hepatotoxicity, teratogenesis, and tumour promotion [13,36]. Although the AHR is well-studied as a mediator of the toxicity, its normal physiological function still remains unclear [37]. Recent research data support a hypothesis that the AHR contribute to the proper functioning of the immune, hepatic, cardiovascular, vascular and reproductive systems [4,38]. AHR was shown to play a significant role in the cross-talk of signalling pathways governing cell proliferation, morphology, adhesion cell migration and cell cycle [38]. It has an important function in the regulation of hematopoietic stem cells (HSCs) [37]. Additionally, overexpression and constitutive activation of the AHR have been observed in various types of tumour [39].

AHR, as a TF, should be able to enter the nucleus, however maintaining of predominantly cytoplasmic compartmentalization is important for its ligand binding [40]. It was shown that human AHR possess, in the N-terminal bHLH domain, opposing signals: NLS (13-39aa) [35] and NES (55-75aa) [41]. Interestingly, Hsp90 is responsible for masking NLS activity in bHLH domain of unligated AHR [34]. Additional NES residing in the PAS domain of mARNT (214-222aa) [42] was shown to be active independently from the ligand binding. This signal sequence is highly conserved in hAHR (220-228aa). Very recently, Tkachenko et al. [40] described another putative NLS (648-671aa) and NES in the C-terminus of hAHR. Activity of this NES depends on the presence of V647 (or I647) residue. Interestingly, these signals are in close proximity or even partially overlapping.

The results of our predictions suggest the possible presence of additional localization signals in AHR (Table 1, Supplementary Materials 1, Supplementary Materials Summary), especially NLS in the PAS-2 domain (area of 247-280aa) and NES (area of 538-552aa) in the C-terminal region.

### 2.2. HIF-1-3α Localization Regulation

The hypoxia-inducible factor α-subunits (HIF-α) are key transcription factors in the mammalian response to oxygen deficiency. To achieve an adequate function, HIF-α levels, subcellular distribution, and activity, have to be tightly regulated [43]. The mammalian hypoxia inducible factor 1α (HIF-1α), plays an essential role in cellular and systemic oxygen homeostasis as cytoplasmic sensor [44]. The HIF-1α/ARNT heterodimer regulates genes transcription activity related to angiogenesis, erythropoiesis, glycolysis, iron metabolism and cell survival [45]. Interestingly, the HIF-α proteins are not only regulated by hypoxia, but also in response to various stresses, growth and coagulation factors, hormones, or cytokines under normoxia conditions [43]. Accumulation of HIF-1α in nucleus is observed during early development of organs, in response to ischaemia and in tumour tissue [45]. Thus, the activation of HIF-1α has been associated with proper embryonic development and with many diseases, such as cancer, stroke, and heart disease that generate a hypoxic microenvironment [44].

Under normoxic conditions, the HIF-1α is ubiquitinated by von Hippel–Lindau tumour suppressor (pVHL), translocated to the cytoplasm and targeted for proteasomal degradation [46]. Contrary, in the hypoxic conditions, HIF-1α becomes stable and is translocated to the nucleus [47], where dimerizes with ARNT creating functionally active complex. Phosphorylation of HIF-1α at S641/S643 residue by Extracellular signal-regulated kinase (ERK) masks an adjacent exportin-1-dependent NES and inhibits HIF-1α nuclear export, thereby increasing its nuclear concentration and transactivation ability [48]. Recently, Mylonis et al. [49] demonstrated an unconventional, controlled by ERK, non-genomic and anti-apoptotic function of HIF-1α. The protein can serve as an early protective mechanism upon oxygen limitation and promote cancer cell resistance to chemotherapy. Also, recently Depping and co-workers [50] proposed, that the modulation of nuclear translocation of HIFs could provide therapeutic applicability to tumours. Interestingly, one of factors influencing HIF localization, is insulin promoting its nuclear translocation [51].

It was shown that human HIF-1α possess two NLSs: one in the bHLH domain (17-33aa) and the other, responsible for import to nucleus in hypoxia, in the C-terminal part of protein (718-721aa) [12]; (717-757aa) [52]. Kallio et al. [12] proposed that PAS-2 is responsible for repression of nuclear import but did not indicate any NES position. Then, Mylonis et al. [48] documented phosphorylation-dependent NES (616-658aa) in the C-terminal part of protein. Importantly, both NLS and NES in C-terminus are located in close proximity. Zheng et al. [53] showed that localization of HIF-1α was cell-type dependent. All these facts confirm complex system of protein localization regulation, involving many localization signals and their precisely balanced control. Interestingly, Chun with colleagues showed that short variant of HIF-1α (1-516aa) without the C-terminal NLS, resides in cytoplasm and is not able to enter nucleus [54]. It suggests the possibility of the presence of additional NES, not detected to date.

Based on in silico analyses of the HIF-1α sequence, performed with various NLS and NES predictors (Table 1, Supplementary Materials 1, Supplementary Materials Summary), we suggest the presence of additional localization signals. While further NLSs were predicted by a single predictor, NES not described previously (area of 558-572aa) was predicted by most of used NES predictors. Putative motif is located in N-terminal area of protein (1-651aa) previously determined as cytoplasmic [12], which confirms the probability of NES presence in this region of protein. We emphasize the need for experimental verification of supposed NES activity in PAS-1 domain (82-97aa).

Documented NLS in bHLH domain of HIF-1α is conserved for HIF-2α (14-30aa), while NLS in C-terminus is conserved for both: HIF-2α (705-742aa) and HIF-3α (561-591aa) [52]. Very recently, HIF-2α NES (705-738aa) and HIF-3α NES (561-586aa) were detected by sequence alignments with *D. melanogaster* homolog SIMA [55]. More precisely, HIF-2α NES was shown to be dependent on exportin-1 and S672 phosphorylation [56]. Very interesting is the fact that C-terminal NLS and NES in both HIF-2α and HIF-3α are overlapping, while for HIF-1α are located in close proximity. It suggests complex and tightly regulated mechanism of discussed proteins translocation between nucleus and cytoplasm. Uniprot data for hHIF-3α protein suggests the presence of NLS in PAS-1 (77-100aa) and NES in PAS-2 domain (230-274aa) “by similarity” with other proteins. However, these sequences are not experimentally verified and none of our predictors (Table 1, Supplementary Materials 1, Supplementary Materials Summary) pointed these fragments as putative subcellular localization signals. However, performed analysis marked other sequence motifs, located in different parts of protein, as putative NLSs and NESs (Table 1, Supplementary Materials 1, Supplementary Materials Summary). For HIF-2α, the most possible NLS are located in bHLH domain (3-33aa) and C-terminus (area of 737-765aa), while highly probable NESs are proposed mostly in the C-terminus (497-511aa; 525-539aa; 698-712aa and 772-786aa). In the case of HIF-3α our predictions appointed additional NLSs in: bHLH domain (area of 7-53aa), linker between PAS-1 and PAS-2 domain (196-226aa) and C-terminal region (438-465aa) and NESs in the bHLH domain (51-70aa), PAS-1 (114-128aa) and C-terminal region (477-497aa).

### 2.3. SIM1-2 Localization Regulation

The other members of bHLH-PAS family: SIM1 and SIM2, are capable of binding mammalian HRE (Hypoxia Response Element) sequences as heterodimers with ARNT. This results in the competition between HIF-1α and SIM proteins for binding to ARNT and DNA, and consequently, in attenuation of hypoxic reporter gene transcription in hypoxia. Such a complex interplay between the bHLH-PAS proteins in cells where the factors are co-expressed, may enable adaptation of the cell to multiple environmental and developmental signals [57].

Conserved NLS in SIM1 (368-388aa) and SIM2 (367-386aa) was described by Yamaki et al. [58] in the middle part of proteins. The deletion of NLS resulted in cytoplasmic localization. Also separated SIM1 fragments: 1-289aa and 295-333aa, were located in the cytoplasm. The latter fragment (295-333aa) comprises highly hydrophobic aa residues and is suggested as NES signal [58]. Such idea is consistent with our prediction, indicating the presence of putative NES in this area (Table 1, Supplementary Materials 1, Supplementary Materials Summary). In the case of SIM2, fragment comprising 530–760aa residues was detected in the cytoplasm [58]. However, no further studies concerning putative NES were performed. The results of our in silico analyses show for both, SIM1 and SIM2, highly probable NLS in bHLH domain (SIM1 2-33aa; SIM2 2-35aa). Additionally, NLS in C-terminus of SIM2 (556-586aa) was predicted both by cNLS Mapper and NLStradamus (Table 1, Supplementary Materials 1, Supplementary Materials Summary). Predictors indicated also some sequences as NES candidates, especially in the bHLH domain.

### 2.4. CLOCK Localization Regulation

Circadian rhythms are internal processes that regulates all physiological functions and activities [59]. These rhythms, generated by the circadian clock, allow organisms to adjust their biology and behaviour to the daily light-dark cycles, as well as nutrition availability [60,61]. Circadian rhythms rely on the negative feedback loops: the gene activation is later repressed by its own protein product and the cycle can reinitiate [61]. The mammalian clock is activated by bHLH-PAS proteins CLOCK [62] and BMAL1 [63], which dimerize to create an active complex [61]. CLOCK/BMAL1 initiate the transcription of its own inhibitors, the PERIOD (PER) and CRYPTOCHROME (CRY) proteins. PER and CRY dimerize in the cytoplasm and translocate to the nucleus to inhibit CLOCK/BMAL1 and to stop further transcription. As suggested, CRY can play a significant role in the animal circadian system as blue-light photoreceptor for photo entrainment of the circadian clock [64,65].

The first studies concerning CLOCK localization revealed that monomeric CLOCK localized mainly in cytoplasm, while in the presence of its heterodimerization partner BMAL1, is shifted to the nucleus [66]. Later, bipartite NLS in the mCLOCK bHLH domain (32-47aa) was confirmed: point mutations of phosphorylated S38 and S42 residues decreased nuclear localization of mutant [67]. Ward et al. hypothesized, that specific interaction with the transcriptional repressor, cytoplasmic Inhibitor of DNA binding (ID2), is responsible for the cytoplasmic localization of CLOCK [68]. Our predictions (cNLS Mapper, Table 1, Supplementary Materials 1, Supplementary Materials Summary) indicate presence of additional putative NLSs located in PAS-2 (area of 256-290aa) and C-terminal region (411-443aa, 451-481aa and 526-553aa). NESs were predicted in PAS-1 (area of 111-127aa) and C-terminal part (546-561aa). Interestingly, predicted NLS and NES in C-terminus are partially overlapping (NLS 526-553aa/NES 546-561aa).

### 2.5. NPAS1-4 Localization Regulation

NPAS1 (MOP5) was detected only in certain regions of the brain [69] and was connected with neurogenesis and schizophrenia [70]. NPAS1 was shown to be able to repress the transactivation functions of both ARNT and ARNT2 [71]. The first localization studies determined NPAS1 as a nuclear protein [70,72]. However, a few years later, Teh et al. [71] revealed the presence of NES located in PAS-2 domain of mNPAS1 (310-317aa). They proposed, that nuclear localization of NPAS1 depends on the dimerization with ARNT. In the absence of this partner, mNPAS1 is located in cytoplasm, as a result of the detected NES activity. Interestingly, Leptomycin B (inhibitor of protein export dependent on exportin-1) did not inhibit activity of this NES [71]. Contrary, the point mutation of this NES resulted in nuclear localization suggesting the activity of NLS/NLSs. However, authors declared no positive result for NLS prediction [71]. We performed in silico analyses on the human NPAS1 sequence using currently accessible NLS and NES predictors (Table 1, Supplementary Materials 1, Supplementary Materials Summary). The results strongly suggest the presence of putative NLSs in bHLH domain (33-76aa) and in the close proximity to PAS-1 (89-128aa) and PAS-2 (250-284aa) domains. Interestingly, additional NESs were predicted for bHLH (64-78aa and 87-101aa) and PAS-2 (273-287aa) domains, suggesting complex pattern of subcellular localization, based on many opposite and partially overlapping signals in NPAS1.

NPAS2 is a gas-responsive TF, which dimerize with BMAL1. Similarly to CLOCK, NPAS2 regulates gene expression as a function of day-night cycle. As a molecular sensor, NPAS2 regulates circadian oscillation of metabolic pathways including heme catabolism. Interestingly, both PAS domains of this protein are able to bind heme. The second candidate for NPAS2 ligand is CO [73]. The localization of monomeric NPAS2 was shown to be mainly nuclear. However, NPAS2 was detected also in cytoplasmic fraction. After heterodimerization with BMAL1 localization was exclusively nuclear [74]. To date, there is no published research concerning localization signals in NPAS2. However, our in silico predictions (Table 1, Supplementary Materials 1, Supplementary Materials Summary), indicate the presence of putative NLSs in different parts of protein. The most interesting signal is NLS in bHLH domain (4-47aa), proposed by all involved predictors. We suggest experimental verification of this motif activity. Importantly, NLS in bHLH domain was detected previously for NPAS2 homolog—CLOCK [67]. Additionally, a candidate NES sequence is located in C-terminus of protein (525-540aa).

NPAS3 is highly homologous to NPAS1 and also expressed in the brain [75]. The first localization study situated 901aa isoform of NPAS3 (Q8IXF0-4) in the nucleus, as a result of activity of bipartite NLS in the C-terminal part (568-585aa) [76]. Very recently, Luoma et al. tested the localization of the canonical human NPAS3 isoform (933aa) in HEK293T cells. The bHLH domain was localized in the cytoplasm, what they discussed as consistent with the result of their predictions with NetNES server [77]. However, no experimental verification of predicted NES was performed. Authors confirmed also the activity of NLS in C-terminus, previously defined by Kamnasaran et al. [76]. The expressed C-terminal part of protein (451-951aa) was detected exclusively in the nucleus. Further analysis revealed that NPAS3 alone is present both in nucleus and cytoplasm, while co-expression with ARNT resulted in mainly nuclear localization [77]. Our predictions (Table 1, Supplementary Materials 1, Supplementary Materials Summary) revealed high probability of the presence of NLSs in bHLH domain (29-78aa), PAS-1 domain (130-161aa), linker between PAS domains (266-297aa) and C-terminal part of protein (727-773aa). Additionally, most of predictors proposed putative NES in bHLH domain (88-103aa). Again, subcellular localization signals with opposite activity and located in close proximity were predicted in bHLH domain of bHLH-PAS protein.

NPAS4 was discovered in mammalian neurons [78], however further studies detected it also in non-neuronal tissues, like in human endothelial cells [79]. NPAS4 expression is highly induced to protect pancreatic β-cells from ER stress [80] and neuronal cells after ischaemia [81,82]. NPAS4 preferably interacts with class II bHLH-PAS partner - ARNT2, but interaction with ARNT/BMAL1 is also possible [78,82]. The first studies concerning subcellular localization of NPAS4 in mammalian cells, revealed strictly nuclear localization of this protein [83]. Shammlo et al. substantiated this finding for mammalian cells culture [81], however authors reported also expression of NPAS4 in the cytoplasm of rat coronal brain tissue [81]. Finally, Sullivan et al. [84] reported that NPAS4 although mostly nuclear in mammalian cells, was also present in the cytoplasm. The cytoplasmic localization of this protein suggests its additional roles in different cellular processes. NPAS4 was shown to induce autophagy in rat primary cortical neurons and to degrade tau proteins involved in the pathogenesis of Alzheimer’s disease and other tauopathies [85]. The discrepancies in localization studies concerning NPAS4, led us to perform a detailed characterization of the subcellular localization motifs in NPAS4 sequence [21]. NPAS4 localized in the nucleus or the cytoplasm of COS-7 and N2a cells. The proportion of nuclear to cytoplasmic NPAS4 was dependent on the glucose concentration in the medium. Furthermore, cytoplasmic localization of NPAS4 was LMB sensitive. In silico analysis suggested the presence of NLSs in the bHLH domain, the PAS-2 domain and the C-terminal region of NPAS4. Accordingly, putative NESs were expected to be located in the bHLH domain, the PAS-2 domain and the C-terminal region of NPAS4 [21]. For the purpose of this review, we have repeated all predictions (Table 1, Supplementary Materials 1, Supplementary Materials Summary), which are generally still consistent. We performed detailed in vivo experiments, which revealed the presence of two overlapping signals: NLS (10-52aa) and NES (26-45aa) in bHLH domain. Simultaneously in the region adjacent to PAS-2 domain and within this domain in close proximity are located: NLS (158-191aa), NES (227-242aa) and putative NLS (285-316aa) [21]. We demonstrated that C-terminal region of NPAS4 contains overlapping NES (591-600aa) and putative NLS (593-622aa). Additionally, we detected NES activity in the 460-580aa region, however this area was not predicted as NES and we were not able to identify precisely the location of specific sequence [21].

## 3. Regulation of the Subcellular Localization of Class II of bHLH-PAS Transcription Factors: ARNT1-4

As mentioned previously, the class I bHLH/PAS proteins dimerize with class II members, to create a functional transcription factor complex, regulating the expression of genes under their control [4]. One of the most common class II representant is the aryl hydrocarbon receptor nuclear translocator (ARNT, HIF-1β), acting as a dimerization partner for several class I proteins: AHR, HIF family, SIM1 and SIM2 [86]. However, each heterodimeric complex cooperate with distinct cis-acting DNA elements, to regulate extremely different genes and pathways. Therefore, ARNT participates in signal transduction pathways engaged in the xenobiotics metabolism, angiogenesis, vasculogenesis, hypoxia response and many various developmental processes [86]. Importantly, several ARNT variants have been identified in mammals: ARNT2, BMAL1 (ARNTL, ARNT3) and BMAL2 (ARNTL2, ARNT4). The defined bHLH and PAS domains are conserved in size and location between all proteins. However, all variants differ in the length of aa sequence [3], what results from a high divergence between their C-terminal parts [86]. Importantly, bHLH-PAS proteins C-termini are believed to be responsible for proteins function modulation and their activity regulation [2]. ARNT and ARNT2 differ in tissue distribution and only ARNT create functional dimer with AHR and SIM proteins [86]. While mRNA of ARNT is expressed in all tissues, mRNA of *Arnt2* was detected only in brain, kidney, and embryos [87]. Similarly BMAL1 and BMAL2, involved in circadian rhythm signalling and expressed in an oscillatory manner [88], present different tissue distribution. BMAL1 transcripts are highly expressed in brain, skeletal muscle and heart, while BMAL2 mRNA is expressed predominantly in the human fetal brain and adult liver [89]. Recently, it was shown that insulin promotes BMAL1 S42 phosphorylation and interaction with 14-3-3 protein affecting localization of this protein by exclusion from nucleus to cytoplasm [90].

The first studies, concerning localization of ARNT were inconclusive. However, the following analysis showed that ARNT is located predominantly in nucleus, both in the absence and in the presence of ligands and suggested a putative NLS, conserved between ARNT proteins, in their N-terminal part [91]. In the same year, Eguchi et al. [92] proposed that ARNT though being mainly nuclear protein, in some conditions can translocate to cytoplasm. Authors detected active NLS in bHLH domain of human ARNT (39-61aa), which mutation resulted in shift of mutant to cytoplasm. The determined NLS motif is conserved for mouse homologues: ARNT and ARNT2, which suggests that its activity could be also conserved for human ARNT2 [92], localized in the nucleus of almost all transfected cells. Inhibition of this NLS activity in ARNT2/R46W mutant, shifts this mutant to the cytoplasm [93]. These findings suggest the presence of NES in both ARNT and ARNT2. Dougherty [86] proposed the presence of active NES in PAS-1 domain of ARNT2 due to the sequence similarity with NES documented for BMAL1 [94]. Finally, we performed in silico analyses in the context of putative NLSs and NESs presence in both human ARNT and ARNT2 (Table 2, Supplementary Materials 2, Supplementary Materials Summary). In the case of hARNT, results indicate high probability of existence of additional to documented previously NLS (area 83-130aa), and NESs located in PAS-1 domain (159-178aa) and adjacent to PAS-2 domain (336-346aa, 417-431aa). In the case of ARNT2, for which no experiment verifying the presence of NLSs/NESs was performed, the putative NLSs in: bHLH domain (73-104aa) linker between PAS-1 and PAS-2 (256-269aa) and putative NESs in PAS-2 domain (310-319aa) and C-terminus (702-711aa, 802-816aa) are interesting.

BMAL1 was shown to localise equally in nucleus and cytoplasm when expressed alone, while localized strictly in nucleus in the presence of NPAS2. To characterise motifs responsible for this protein shuttling, Kwon et al. [94] performed in silico analysis of murine BMAL1. They proposed as putative NLSs, sequences in 36-40aa and 82-88aa areas, while NESs in 109-116aa, 142-152aa and 360-369aa areas. Performed mutagenesis studies revealed, that active NLS is located in proximity to bHLH domain (36-40aa), while active NESs are located in PAS-1 (142-152aa) and PAS-2 (360-368aa) domains [94]. Interestingly, though Kwon et al. suggested, that putative NLS (82-88aa) is not active, Tamaru et al. [95] showed that for nuclear localization of BMAL1, phosphorylation of S90, which is located adjacent to predicted NLS, is necessary [94]. We performed additional NLS/NES predictions, using currently available servers (Table 2, Supplementary Materials 2, Supplementary Materials Summary). While results of NES prediction were mostly consistent with these performed by authors, we found further putative NLSs located in linker between PAS domains (244-279aa) and in the C-terminus of protein (471-502aa).

Ikeda with coworkers [89] documented the presence of two NLSs (13-16aa and 32-35aa) in N-terminal part of BMAL2. Although deletion of N-terminal part of protein resulted in cytoplasmic localization of mutant [89], no in silico or experimental analysis were performed for identification of putative NES. We performed predictions of both putative NLS and NES motifs (Table 2, Supplementary Materials 2, Supplementary Materials Summary). Results suggest the presence of three signals not described to date: NLS (103-127aa) and NES (140-156aa) in bHLH domain and an additional NLS (279-322aa) in linker between PAS domains. Interestingly, second putative NES (238-252aa) is located close to the same region between PAS domains.

## 4. Regulation of Subcellular Localization of *Drosophila melanogaster* bHLH-PAS Transcription Factors

Insect growth and development are controlled by the coordinated action of two hormones: 20-hydroxyecdysone (20E) and juvenile hormone (JH) [96]. Although the 20E receptor has been studied extensively, the identity and function of the JH receptor has long remained elusive [97]. Finally, in 2011, MET has been confirmed as JH receptor [98]. MET belongs to the bHLH-PAS family and prevents precocious metamorphosis of *D. melanogaster* during development [99]. The deletion of *met* gene is lethal to most species of insects, however in *D. melanogaster* exists MET paralog—GCE, ensuring survival of the *met* null mutants [100]. The functions of paralogues are not fully redundant and proteins exhibit tissue-specific distribution [101]. It was shown that MET is able to create inactive homodimers and heterodimers with GCE, in the absence of JH [102].

The first studies concerning MET subcellular localization were highly inconsistent [103,104]. Later, we have shown, that MET is able to translocate from the cytoplasm to the nucleus [19]. The translocation is associated with the presence of the Hsp83 (*Drosophila* homolog of Hsp90), which seem to be indispensable for JH binding and MET transport to nucleus [105]. We have shown that MET was mainly located in nucleus. However, some cells presented strictly cytoplasmic distribution. We identified dominant NLS in PAS-1 domain (98-102aa) and JH-inducible NLS (482-498aa) in PAS-2 domain. MET NESs are located in PAS-1 (126-139aa) and PAS-2 (446-456aa) as well as in the C-terminus. As no NES motif was predicted in the C-terminal area, this signal was difficult to identify [19]. Finally, we determined location of this NES (589-631aa) by sequence alignment with GCE [20]. Interestingly, in repeated for this review prediction, we got some positive result in this area (Table 3, NetNES L603, Supplementary Materials 3, Supplementary Materials Summary), however, still with very low probability. As mentioned previously, MET and GCE functions are not fully redundant. We showed that, in contrast to MET, GCE was distributed in both nucleus and cytoplasm. The homology between GCE and MET NLSs and NESs activities occurs only in the PAS-2 domains. Dominant NLS localized in MET PAS-1 domain is absent in GCE, while unique for GCE, NLS in C-terminus (840-859aa) can be distinguished. Interestingly, activity of this signal is crucial for ligand activation of NLS (580-610aa) in PAS-2 domain and GCE translocation to the nucleus [20]. It is worthy to note, that in our studies [20], we used 689aa isoform of GCE, which lacks 270 N-terminal aa residues. According to personal communication with A. Baumann, GCE used in that study and its longer variant [106], deposited in UniprotKB database as Q9VXW7, show similar function when expressed in transgenic *D. melanogaster*. For purpose of this review, we performed in silico predictions with use of the full-length GCE (Table 3, Supplementary Materials 3, Supplementary Materials Summary) and we renumbered experimentally documented sequences according to 959aa isoform. As suspected, there is no highly probable NLS motif in the first N-terminal 270aa area, however NetNES predicted 44-50aa as NES, which should be experimentally verified. We believe that all others active signals in MET and GCE proteins are well described [19,20], and all other predicted motifs are artefacts (Table 3). However, we realise that neighbouring or overlapping signals with opposite activities can mask each other and their detection can be difficult. On the other hand, we have revealed the presence of signals, not predicted by any of used servers. Therefore, we can conclude that only very systematic and detailed studies can lead to NLS/NES motif determination.

*D. melanogaster* is highly resistant to the lack of oxygen [107]. Interestingly, its hypoxia-responsive system shows significant similarity to mammalian system and is based on the activity of two bHLH-PAS proteins: SIMA and TANGO [107,108]. SIMA, as a homologue to mammalian HIF-1α, is sensitive to oxygen tension and shuttles continuously between the nucleus and the cytoplasm. It is mostly cytoplasmic in normoxi and accumulated in the nucleus in hypoxia. The nuclear export is mediated by exportin-1 [55]. The second protein - TANGO is expressed constitutively, similarly to its mammalian class II homolog - ARNT, and dimerize with SIMA to create active heterodimer regulating appropriate gene expression [107]. As shown, TANGO subcellular localization is developmentally regulated. TANGO with no dimerization partner is found predominantly in the cytoplasm, while in the presence of SIMA and Trachealess (TRH) is translocated to the nucleus [1,109]. Romero et al. [55] performed detailed in silico analysis of the SIMA sequence and determined putative NLSs in 537-568aa, 1210-1230aa, and 1406-1409aa areas, and putative NESs in 92-101aa, 115-124aa, 1011-1020aa and 1131-1140aa areas. They proved experimentally the NLS active motif (1210-1230aa), being a highly conserved sequence between all hypoxia factors. Also, active NESs (92-101aa and 115-124aa) were determined in the SIMA bHLH domain [55]. We have used currently available predictors for SIMA sequence analysis and we got some additional positive results (Table 3, Supplementary Materials 3, Supplementary Materials Summary). We suggest, that especially interesting would be experimental verification of putative NLS in bHLH domain of SIMA (46-91aa), predicted with high probability by most of used servers.

In the case of TANGO, no NLS/NES motifs were documented to date, as no detailed studies were performed. Using ClustalX server (www.clustal.org), we performed alignment of TANGO with ARNT sequence. However, it was no conservation in ARNT documented NLS area (Supplementary Materials 3, Supplementary Materials Summary). Our predictions suggest NLS activity in bHLH and PAS-2 domains, while NESs are proposed in PAS-1 and PAS-2 domains. We would recommend testing these experimentally, especially the activity of predicted with high probability NLS (23-54aa) in bHLH domain and putative NES (259-272aa) within PAS-2 domain.

**Figure 2:**
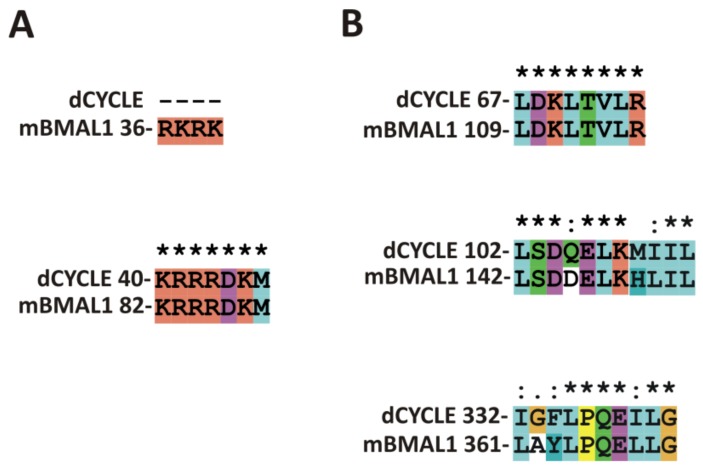
ClustalX alignment of DmCYCLE/hBMAL1 sequences in area of hBMAL1 localization signals [94]. (**A**) Sequences encompassing NLSs (**B**) Sequences encompassing NESs.

CYCLE is a *Drosophila* ortholog of mammalian BMAL1 regulating circadian rhythms. We performed alignment of CYCLE with studied previously mBMAL1 isoform 2 (Q9WTL8-2) [94] sequence using ClustalX (Figure 2). Additionally, we performed signals predictions (Table 3, Supplementary Materials 3, Supplementary Materials Summary). Obtained results suggest the presence of NLSs in: bHLH domain (41-71aa) which is consistent with conservation of mBMAL1 NLS in this area (82-88aa) (Figure 2A) and in the linker between PAS-1 and PAS-2 domains (229-262aa). Putative NLS (229-262aa) should be experimentally verified as not conserved in BMAL1. In bHLH domain of CYCLE exists NES (63-75aa) present also in mBMAL1 (109-116aa) (Figure 2B) and verified as not active [94]. Prediction did not point any further signals, however alignment shows conservation of active mBMAL1 NESs: in PAS-1(142-152aa)/ CYCLE(102-112aa) and PAS-2 (361-369aa)/CYCLE(332-340aa) (Figure 2B).

## 5. Concluding Remarks

The subcellular distribution of the bHLH-PAS proteins is one of mechanisms regulating their functions and activities. Recently it was shown, that in addition to functioning as transcriptional regulators, some of bHLH-PAS transcription factors, when located in cytoplasm, take part in regulation of translational processes [110,111,112].

**Figure 3:**
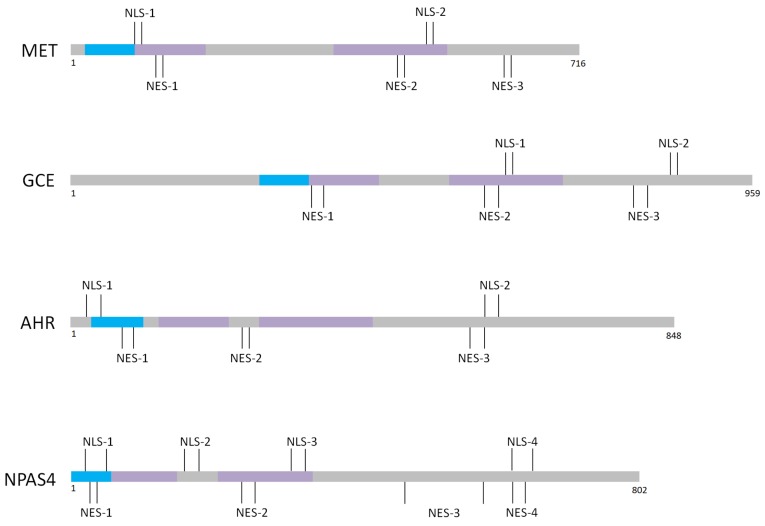
Schematic representation of documented NLSs/NESs distribution within the MET [19], GCE [20], AHR [35,40,41,42] and NPAS4 [21] proteins. bHLH domains are shown in blue, PAS domains are shown in violet. For details see Supplementary Material Summary.

Most of bHLH-PAS family members are known to possess NLS and/or NES motifs in defined bHLH /PAS domains (Figure 3). The presence of localization signals within defined domains responsible for specific interactions and ligands binding can make the regulation of subcellular translocation highly complex interplay of different factors. Importantly, in the case of many transcription factors presented in this review, at least one of NLS/NES motifs is located also in the C-terminal region (Figure 3) [19,20,21,48,52,55,76] responsible for interaction with activators/repressors influencing protein activity.

The simultaneous presence of NLS and NES with similar strength can be the reason for ubiquitous localization of proteins, or specific parts of proteins in cells and/or difficulties with precise signal detection. The presence of multiple localization signals with opposing activities enables complex and precisely balanced regulation of bHLH-PAS TFs shuttling, by masking and unmasking of specific localization signals in different parts of proteins in response to different stimuli. Interestingly, predicted in our review NLSs/NESs are often overlapping or located in close proximity. We believe that subcellular localization of presented TFs depends on the integrated action of several (no single!) localization sequences, including those not identified to date. The activity of signals can be modulated by ligands, posttranslational modifications (PTMs) and interactions with partner proteins. We emphasize the need of additional detailed studies to identify not described NLS/NES motifs.

## Figures and Tables

**Table 1:** Summary of predicted and documented nuclear localization signals (NLSs) and nuclear export signals (NESs) in Class I bHLH-PAS proteins.

Protein Name	Domains According to UniProt	Predicted NLS aa Area	Predicted NES aa Area	EXPERIMENTAL NLS	EXPERIMENTALNES
**hAHR** UniProtKB - P35869	27-80 bHLH 111-181 PAS-1 275-342 PAS-2 348-386 PAC	11-43abcdef 78-106a 247-280abf 368-398a 640-668a 746-776a	47-56g 59-77ghi 258-272gi 417-431gi 538-552fg 735-746gi	13-39 [35] 648-671 [40]	55-75 [42] 214-222 [42] NES around V647 [40]
**hHIF-1α** UniProtKB - Q16665	17-70 bHLH 85-158 PAS-1 228-298PAS-2 302-345 PAC	3-41abde 58-60e 68-97a 155-160b 245-278a 365-394a 709-746abcdf	37-46gh? 52-69gi 82-97fg 390-399h 558-572ghi? 627-647i L737h?	17-33 [86] 718-721 [12] 717-757 [52]	616-658 [48]
**hHIF-2α** (EPAS1) UniProtKB - Q99814	14-67 bHLH 84-154 PAS-1 230-300 PAS-2 304-347 PAC	3-33abcde 144-177ab 680-735ab 737-765ab 793-840a	33-47gi 49-64hi 82-102ghi? 497-511ghi? 525-539ghi? 698-712gi 772-786gi	14-30 705-742 [52]	705-738 [55]
**hHIF-3α** UniProtKB - Q9Y2N7	14-67 bHLH 82-154 PAS-1 227-297 PAS-2	7-53abe 153-182a 196-226a 361-392a 438-465a 512-574ab 563-598abc 590-620a	34-43g 51-70ghi 114-128hi L221h? 477-497ghi 603-617gi 629-643gi	77-100 Uniprot 561-591 [52]	230-274 Uniprot 561-586 [55]
**hSIM1** UniProtKB - P81133	1-53 bHLH 77-147 PAS-1 218-288 PAS-2 292-335 PAC	2-33ab 74-104a 163-197a 235-267a 347-386ab 600-628a 703-734a	19-33gi 42-56i 231-249gi? 340-347h	368-388 [58]	295-333? [58]
**hSIM2** UniProtKB - Q14190	1-53 bHLH 77-149 PAS-1 218-288 PAS-2 292-335 PAC	2-35abe 74—105a 163-195a 351-386ab 556-586ab 637-665a	8-17g 19-33i 35-49i 42-56i 65-74g 102-116i? 275-289gi? 325-344hi?	367-386 [58]	-
**hCLOCK** UniProtKB - O15516	34-84 bHLH 107-177 PAS-1 262-332 PAS-2 336-379 PAC	27-85abcde 256-290a 411-443a 451-481a 526-553a	111-127hi 507-516g 546-561fgi? 588-597g 798-812i?	32-47 mouse [67]	-
**hNPAS1** UniProtKB - Q99742	45-98 bHLH 135-207 PAS-1 293-359 PAS-2 365-408 PAC	33-76abe 89-128ab 250-284ab 456-487abf	64-78gi 87-101h?i 273-287i 300-320ghi	-	310-317 [71]
**hNPAS2** UniProtKB - Q99743	9-59 bHLH 82-152 PAS-1 237-307 PAS-2 311-354 PAC	4-47abcde 214-265a 502-532a 698-730a 803-824b	25-29i 88-102hi 492-501g 525-540fgi 545-559i?	-	-
**hNPAS3** UniProtKB - Q8IXF0	51-104 bHLH 147-217 PAS-1 319-389 PAS-2 363-406 PAC	1-18e 29-78ab 130-161ab 266-297ab 578-635abcdf 645-674a 727—773ab	70-84gi? 88-103ghi 284-298gi 333-347gi? 871-885gi? 903-921i?	568-585 [76]	NES in bHLH [77]
**hNPAS4** UniProtKB - Q8IUM7	1-53 bHLH 70-144 PAS-1 203-273 PAS-2 278-317 PAC	8-39ab 158-191ab 193-211b 220-251a 283-314a 593-621a	20-46 ghi 230-245h?i 394-408i? 590-604ghi? 664-678gi	10-52 rat 158-191 285-316? 593-622? [21]	29-45 rat 227-2424 60-580? 591-600 [21]

NLS/NES signal was predicted by (a) cNLS Mapper (b) NLStradamus (c) NucPred (d) PSORT II (e) SeqNLS (f) ELM (g) NES Finder (h) NetNES (i) LocNES. (?)—low probability of prediction. (red color) - predicted sequence was proven experimentally. See Supplementary Materials Summary and Supplementary Materials 1 for prediction details.

**Table 2:** Summary of predicted and documented NLSs and NESs in Class II bHLH-PAS proteins.

Protein Name	Domains According to UniProt	Predicted NLS aa Area	Predicted NES aa Area	EXPERIMENTAL NLS	EXPERIMENTAL NES
**hARNT** **HIF-1β** UniProtKB - P27540	89-142 bHLH 161-235 PAS-1 349-419 PAS-2 424-467 PAC	34-61abdef 83-130ab 151-182a 260-291a 310-339a 390-421a	159-178hi 274-288i 336-345g 417-431i 671-685i?	39-61 [93]	-
**hARNT2** **HIF-2β** UniProtKB - Q9HBZ2	63-116 bHLH 134-209 PAS-1 323-393 PAS-2 398-441 PAC	15-29e 34-85b 39-66aef 73-104acd 256-269abf 364-394a 591-619a	133-150h 310-319gh? 702-711g	42-64 by similarity to ARNT [93]	132-144 by similarity to BMAL1 [87]
**hBMAL1 ARNTL,** **ARNT3** UniProtKB - O00327	78-131 bHLH 146-213 PAS-1 328-394 PAS-2 401-444 PAC	23-45acdef 37-91b 82-113acd 174205a 244-279ab 291-322a 471-502a	105-121ghi 142-159hi?	36-41 active 81-87 not active mouse [94]	109-116 not active 142-152 active 361-369 active mouse [94]
**hBMAL2** **ARNTL2 ARNT4** UniProtKB - Q8WYA1	107-160 bHLH 178-250 PAS-1 357-475 PAS-2 432-475 PAC	1-10e 19-26e 35-60abcdef 103-127abcd 209-240a 279-322ab 444-474a 554-586a	140-156ghi 238-252g 463-477f	13-16 32-35 mouse [90]	-

NLS/NES signal was predicted by (a) cNLS Mapper (b) NLStradamus (c) NucPred (d) PSORT II (e) SeqNLS (f) ELM (g) NES Finder (h) Net NES (i) LocNES. (?)—low probability of prediction. (red color) - predicted sequence was proven experimentally. See Supplementary Materials Summary and Supplementary Materials 2 for prediction details.

**Table 3:** Summary of predicted and documented NLSs and NESs in selected *Drosophila melanogaster* bHLH-PAS proteins.

Protein Name	Domains According to UniProt	Predicted NLS	Predicted NES	EXPERIMENTAL NLS	EXPERIMENTAL NES
**MET** UniProtKB - Q9VYW2	36-89 bHLH 143-185 PAS-1 403-508 PAS-2 Domains according to Uniprot and [19]	34-72abe 68-115b 98-128acdf 252-285ab 296—330a 489-519a 532-554a 628-662ab	130-142g 262-276gi 425-443ghi? 446-460i? 542-556i? L603h?	98-102 absent in GCE 482-498 present in GCE [19]	126-139 present in GCE 446-456 present in GCE 598-631 present in GCE [19]
**GCE** UniProtKB - Q9VXW7	277-330 bHLH 341-416 PAS-1 Uniprot 272-324 bHLH339-425 PAS-1 531-660 PAS-2 According to [20] *	12-25e? 39-56e? 278-323ab 424-458ab 469-499a 521-554a 625-656abf 838-860adf	44-50h 347-361hi 567-576g 582-596i? 650-659g 778-792i?	618-634 ** present in MET 840-859** absent in MET [20]	343-359** present in Met 580-610** present in MET 762-795** present in MET [20]
**SIMA** UniProtKB - Q24167	72-125 bHLH 167-249 PAS-1 307-377 PAS-2 381-422 PAC	46-91bde 150-180a 347-374a 383-414a 545-546b 554-591abd 1102-1131a 1212-1232af 1288-1316a 1408-1417af	90-105fghi? 114-130gh?i 173-189fh?i 477-491i 636-650i? 1006-1020i 1064-1078 1125-1139gi?	537-568 not active? 1210-1230 activeConserved with HIF-1-3α 1406-1409 not active? [55]	92-101 active 115-124 active 1011-1020 not active? 1131-1140 not active? [55]
**TANGO** UniProtKB - O15945	13-66 bHLH 85-156 PAS-1 271-341 PAS-2 346-389 PAC	11-52b 23-54acd 167-196a? 207-237a? 288-320a	83-97hi 259-272ghi? 624-638h?i	-	-
**CYCLE** (BMAL1) UniProtKB - O61734	30-83 bHLH 104-175 PAS-1 297-367 PAS-2 372-413 PAC	25-46 bcd 41-71ae 210-240a 229-262abf	50-59g 63-75gh? 102-111h?	-	-

NLS/NES signal was predicted by (a) cNLS Mapper (b) NLStradamus (c) NucPred (d) PSORT II (e) SeqNLS (f) ELM (g) NES Finder (h) Net NES (i) LocNES. (?)—low probability of prediction. (red color) - predicted sequence was proven experimentally. See Supplementary Materials Summary and Supplementary Materials 3 for prediction details. * GCE isoform used by Greb-Markiewicz et al. [20] is 270aa N-terminally shorter and no longer exists in Uniprot database. **aa renumbered according to UniProtKB—Q9VXW7 sequence.

## Supplementary Materials

Supplementary materials can be found at https://www.mdpi.com/1422-0067/20/19/4746/s1.

## Abbreviations

20E	20-hydroxyecdysone
bHLH-PAS	basic helix-loop-helix/ Period-ARNT-Single minded
AHRHIF	Aryl hydrocarbon ReceptorHypoxia Inducible Factor
ARNT	Aryl Hydrocarbon Nuclear Translocator
ARNTL	Aryl hydrocarbon receptor nuclear translocator-like protein
cNLS	classical NLS
CRM1	Chromosome region maintenance 1 protein homolog
CRY	Cryptochrome
ER	Endoplasmatic reticulum
ERK	Extracellular signal-regulated kinase
GCE	germ cell-expressed protein
HRE	Hypoxia Response Element
HSCs	hematopoietic stem cells
JH	juvenile hormone
LMB	Leptomycin B
MET	Methoprene tolerant protein
NES	nuclear export signal
NLS	nuclear localization signal
NPAS	Neuronal PAS domain-containing protein
PAC	C-terminal to PAS domain
PER	Period protein
pVHL	von Hippel–Lindau tumor suppressor
RPD	repression domain
SIM	Single-minded homolog protein
SIMA	similar
TAD	transcription activation domain
TF	transcription factor
TRH	Trachealess protein
