# Supplementary material for: Subcellular Localization Signals of bHLH-PAS Proteins: Their Significance, Current State of Knowledge and Future Perspectives
Source: Int J Mol Sci. 2019 Sep 24;20(19):4746. doi: 10.3390/ijms20194746 (PMC6801399; doi:10.3390/ijms20194746)
Supplement: Supplementary file 1 [file ijms-20-04746-s001.zip › SupplementaryIJMS/Supplementary material Summary.pdf]

Subcellular Localization Signals of bHLH-PAS Proteins: their Significance, Current State of Knowledge and Future Perspectives

Beata Greb-Markiewicz [1,*] and Marta Kolonko [1]

Supplementary Materials Summary

Predictors used for NLS prediction:

cNLS Mapper	a
NLStradamus	b
NucPred	c
PSORT II	d
SeqNLS	e
ELM	f

Predictors used for NES prediction:

ELM	f
NES Finder	g
NetNES	h
LocNES	i

Prediction summary for Table 1

hAhR

cNLSMapper

11-20 score	11
13-41	5.4
34-62	4.9
78-106	3.0
250-280	3.1
368-398	3.7
640-668	2.7
746-776	2.9

NLStradamus

12-42 Posterior 0.3

247-254

NucPred
13-17 score 0.94

PSORT II
13-19 score 0.94
35-41

SeqNLS

11-24 score	0.887
27-43	0.886

ELM
NLS

13-18	1.276e-03
249-254	7.252e-04

NES

538-552	7.626e-04

NES Finder
47-56
60-69
63-72
259-268
420-429
539-548
735-744

NetNES
64-77
577-584?

LocNES

59-73	0.746
258-272	0.138
417-431	0.118
732-746	0.174

hHIF1α

cNLS Mapper

8-36 score	2.6

68-97	2.9
245-278	2.8
365-394	2.7
716-725	8.0
717-746	3.2

NLStradamus

3-41 Posterior 0.1
155-160
709-723

NucPred

718-721 score 0.94

PSORT II

17-33 score 0.33
718-721

SeqNLS

10-33	score	0.964
34-39		0.696
58-60		0.568

ELM

NLS

717-723	7.252e-04

NES

82-97	7.626e-04

NES Finder

37-46
60-69
84-93
559-568

NetNES

L46?
64-80
390-399
559-566?

L583?
L737?

LocNES

52-66	0.122
443-457	0.284
558-572	0.190
627-641	0.365

hHIF2α

cNLS Mapper

5-33	score	2.5
20-42		2.6
65-94		3.8
144-177		2.9
682-711		4.1
705-735		5.4
737-765		3.2
793-822		4.0

NLStradamus

3-33 Posterior 0.1
156-160
680-710
738-741

NucPred

14-16 score 0.86

PSORT II

14-30 score 0.02

SeqNLS

8-30 score 0.916
34-40 score 0.844

ELM

No NLS/NES

NES Finder

34-43
92-101
498-507
526-535
699-708
773-782

NetNES
56-64
L92?
498-501?
L526?

LocNES

33-47	0.135
49-63	0.190
82-96	0.158
116-130	0.105
497-511	0.234
525-539	0.038
564-578	0.124
698-712	0.354
772-786	0.166

hHIF3α

cNLS Mapper

20-53	score	2.3
153-182		2.6
196-226		3.3
361-392		2.3
438-465		3.6
542-574		3.7
567-598		6.8
590-620		3.0

NLStradamus
7-30 Posterior 0.1
512-533
563-597

NucPred
569-571 score 0.90

PSORT II
No NLS

SeqNLS
24-35 score 0.805

ELM
No NLS/NES

NES Finder
34-43
57-66
487-496
603-612
630-639

NetNES
51-61
122-127
L221?
478-494

LocNES

56-70	0.265
114-128	0.256
477-491	0.387
603-617	0.500
629-643	0.547

hSIM1

cNLSMapper

4-33 score	3.0
74-104	2.3
163-197	3.4
235-267	2.8
347-377	2.7
600-628	3.6

703-734 2.6

NLStradamus

2-24 Posterior 0.1

365-386

PSORT II

No NLS

SeqNLS

No NLS

ELM

No NLS/NES

NES Finder

20-29

231-240

NetNES

340-347

LocNES

19-33	0.254
42-56	0.368
235-249	0.113

hSIM2

cNLS Mapper

6-35	score	3.7
74-105		3.5
163-195		3.5
235-267		2.8
351-378		6.1
454-484		2.6
556-586		2.4
637-665		2.9

NLStradamus

2-25 Posterior 0.1

363-386
556-572

NucPred
No NLS

PSORT II
No NLS

SeqNLS
5-20 score 0.598

ELM
No NLS/NES

NES Finder
8-17
65-74
276-285

NetNES
332-344

LocNES

19-33	0.228
35-49	0.352
42-56	0.338
102-116	0.130
190-204	0.105
234-248	0.136
275-289	0.083
325-339	0.026

hClock

cNLS Mapper

39-72	score	7.3
256-290		3.4
411-443		3.5
451-481		3.9
526-553		3.1

NLStradamus
27-85 Posterior 0.1

NucPred
44-47 score 0.94

PSORT II
44-47
82-85
Score 0.03

SeqNLS

6-16	score	0.522
44-47		0.862

ELM
No NLS
NES

546-561	7.626e-04

NES Finder
507-516
548-557
588-597

NetNES
115-125

LocNES

111-127	0.373
414-428	0.119
547-561	0.122
798-812	0.136

hNPAS1

cNLS Mapper

33-61	score	3.1
55-87		2.9

89-122	2.2
250-284	3.0
458-487	2.2

NLStradamus

33-76 Posterior 0.1
96-128
258-259
456-475

NucPred

No NLS

PSORT II

No NLS

SeqNLS

41-49	score	0.447
52-60		0.351

ELM

NLS 460-466 7.252e-04
No NES

NES Finder

65-74
88-97
300-309
304-313
306-315

NetNES

L97?
309-318

LocNES

64-78	0.245
87-101	0.295
273-287	0.780
306-320	0.211

hNPAS2

cNLS Mapper

14-47	score	7.3
214-242		3.4
231-265		3.7
502-532		2.2
698-730		2.5

NLStradamus

4-46 Posterior 0.1
803-816
821-824

NucPred

19-22 score 0.89

PSORT II

19-22 score -0.16

SeqNLS

19-22 score 0.873

ELM

No NLS
NES 525-539 7.626e-04

NES Finder

492-501
527-536

NetNES

90-102

LocNES

25-29	0.348
88-102	0.398
526-540	0.150
545-559	0.187

hNPAS3

cNLS Mapper

29-58 score	3.4
130-161	3.0
266-297	2.7
360-390	2.5
497-527	2.0
578-608	3.9
606-640	5.1
645-674	3.2
727-755	3.6

NLStradamus

44-78 Posterior 0.1
133-136
273-274
582-635
754-773
596-616 Viterbi Path

NucPred

599-606 score 0.96

PSORT II

599-606
600-618
Score 3.61

SeqNLS

1-18 score 0.908

ELM

NLS
596-609 score 1.276e-03
No NES

NES Finder

71-80
94-103

285-294
334-343
872-881

NetNES
88-98

LocNES

70-84	0.101
88-102	0.507
284-298	0.266
333-347	0.084
871-885	0.079
903-921	0.187

hNPAS4

cNLS Mapper

10-39	score	2.5
158-191		2.1
220-251		3.2
283-314		2.3
593-621		3.0

NLStradamus
8-16 Posterior 0.1
158-178
193-211

NucPred
No positive result

PSORT II
No specified NLS

SeqNLS
No positive result

ELM
NLS
No positive result
NES

593-607 7.626e-04

NES Finder

20-29

591-600

665-674

NetNES

26-45

230-245?

L598?

LocNES

19-33	0.104
32-46	0.664
220-234	0.531
230-244	0.343
394-408	0.143
590-604	0.124
664-678	0.602

Prediction summary for Table 2

hARNT

cNLSMapper

39-61 score 8.6

99-130	4.6
151-182	2.3
260-291	2.3
310-339	2.6
390-421	3.1

NLStradamus

34-47

83-128

NucPred

No NLS

PSORT II

42-45
99-102
Score 0.09

SeqNLS

39-50 score 0.820

ELM

NLS

39-46 Probability 1.276e-03

No NES

NES Finder

336-345

NetNES

159-178

LocNES

159-173	score	0.222
274-288		0.308
417-431		0.198
671-685		0.104

hARNT2

cNLS Mapper

42-66 score	10.5
73-104	4.0
256-266	6.0
364-394	3.4
591-619	2.1

NLStradamus

34-85
95-102
258-269

NucPred

73-76 0.89

PSORT II
73-76 score -0.16

SeqNLS

15-29	0.862
39-51	0.883

ELM
NLS

42-49	1.276e-03
260-265	

NES
No positive result

NES Finder
310-319
702-711

NetNES
133-150
V319?

LocNES
No NES candidate

hBMAL1 (ARNT3)

cNLS Mapper

34-44 score	5.0
83-113	5.4
174-205	3.1
244-277	4.6
291-322	3.0
471-502	3.8

NLStradamus
37-91 posterior 0.1
258-279
472-499

NucPred
score 0.87
37-40
82-85

PSORT II
37-40
82-85
Score 0.15

SeqNLS
23-45 score 0.879

ELM
NLS
37-42 Probability 1.276e-03
NES
No positive result

NES Finder
105-114
108-117

NetNES
V114?
142-159

LocNES

107-121	0.636
-115-129	0.150
151-165	0.048

hBMAL2 (ARNT4)

cNLS Mapper
47-57 score 7.0

114-123	6.0
209-240	3.7
279-314	3.3

444-474	2.2
554-586	2.6

NLStradamus
35-60
103-127
285-322

NucPred
49-52
117-120
Score 0.85

PSORT II
49-55
117-120
301-307
NLS score 1.18

SeqNLS

1-10	0.893
19-26	0.791
44-59	0.895

ELM
NLS
49-55 Probability 1.276e-03
NES
463-477 Probability 7.626e-04

NES Finder
140-149
143-152
239-248

NetNES
140-152?

LocNES
141-156 score 0.614

238-252	0.225
463-477	0.084

Prediction summary for Table 3

MET

cNLS Mapper

38-72 score	2.9
98-128	3.8
252-285	3.9
296-330	3.5
489-519	3.0
523-554	2.7
628-662	2.6

NLStradamus

38-52
68-115
252-260
636-640

NucPred

98-102 score 0.82

PSORT II

98-101 score 0.15

SeqNLS

34-43 score 0.548

ELM

NLS

98-104 Probability 1.276e-03

NES

No positive result

NES Finder

263-272
431-440

NetNES
130-142
L443?
L603?

LocNES

130-144 score	0.658
262-276	0.094
425-439	0.064
446-460	0.044
542-556	0.178

GCE

Full length isoform

cNLS Mapper

279-323 score	3.3
424-454	3.0
469-499	3.1
521-554	3.2
625-655	2.7
838-860	12.5

NLStradamus
278-294
426-458
647-656

NucPred
No predicted NLS

PSORT II
840-856 score 0.02

SeqNLS

12-25 score	0.399
39-56	0.346

ELM

NLS

646-652	Probability 7.252e-04
840-859	Probability 2.588e-04
852-858	Probability 1.276e-03

NES

No positive result

NES Finder

567-576

650-659

NetNES

44-50

L329

L352-359

LocNES

35-49	score	0.073
347-361		0.533
582-596		0.044
778-792		0.016

SIMA

cNLS Mapper

150-180	score	2.9
347-374		3.1
383-414		3.0
561-591		3.3
1102-1131		2.8
1212-1232		6.4
1288-1316		3.3
1409-1417		3.0

NLStradamus

46-91

545-546

554-581

NucPred

No NLS prediction

PSORT II

63-69
72-88
562-568
1408-1414
NLS score 1.64

SeqNLS

4-32	score	0.902
47-59		0.605

ELM

NLS

1212-1232	2.588e-04
1408-1413	1.276e-03

NES

90-105	7.626e-04
173-189	

NES Finder

92-101
115-124
121-130
1007-1016
1129-1138

NetNES

L101?
122-124?
L183?

LocNES

91-105	score	0.254
114-128		0.318
120-134		0.139

175-189	0.398
477-491	0.437
636-650	0.226
1006-1020	0.235
1125-1139	0.130

TANGO

cNLS Mapper

23-54 score	4.6
167-196	2.2
207-137	2.7
288-320	3.2

NLStradamus (cutoff 0.1)
11-52
170

NucPred
23-26
Score 0.76

PSORT II
23-26
NLS score -0.16

SeqNLS
No predicted NLS

ELM
No NLS/NES positive result

NES Finder
259-268

NetNES
83-97
V268?
I634?

LocNES

83-97	score	0.352
258-272		0.036
624-638		0.539

CYCLE

cNLS Mapper

41-71	score	4.8
176-209		3.3
210-240		4.3
232-262		3.4

NLStradamus (cutoff 0.1)
25-46
229-246

NucPred score 0.90
40-44

PSORT II
40-44
NLS score -0.16

SeqNLS
55-60 score 0.659

ELM
NLS

235-240	7.252e-04
236-241	1.276e-03

NES
No NES positive result

NES Finder
50-59
63-72
66-75

NetNES

63-75?

102-111?

L213?

LocNES

No NES candidates

Supplementary Material for Figure 3

Sequences of documented NLS/NES motifs within MET, GCE, AHR and NPAS4 proteins

MET:

length	716
HLH	38-87
PAS-1	137-186
PAS-2	403-510

NLS: PAS-1 (98-102), PAS-2 (482-498)

NES PAS-1 (126-139), PAS2 (446-456), C-terminus (598-631)

98-RRRKK-102; NLS-1

126-HLTDTLMQLLDCCFL-139; NES-1

446-VRWVIVALRQM-456; NES-2

482-TKFLEVDRGSNKVHSF-498; NLS-2

598-TPALALVPPAPSSVKSITKSISVVNVTAAKLR-631; NES-3

GCE:

length	959
HLH	279-325
PAS-1	352-407
PAS-2	539-646

NLS: PAS-2 (618-634), C-terminus (840-859)

NES PAS-1 (343-359), PAS-2 (580-610), C-terminus (761-795)

343-QITDTLMQLLDCCFLTL-359; NES-1

580-DDVRWVIVALRQMYDCNSSYGESTYRLFTRN-610; NES-2

618-SKGYLEIDKETNKVHSF-634; NLS-1

840- KRGSTAHVETEEKLSKRRF-859; NLS-2

762-TPPLALVPPEASSVKSAITKSISVVNVTAAKLR-795; NES-3

AHR:

length	848
HLH	37-78
PAS-1	113-226
PAS-2	297-382

NLS: bHLH (13-39), C-terminus (648-671)

NES: bHLH (55-75), PAS-1 (214-222), C-terminus (around V647)

13-RKRRKPVQKTVKPIPAEGIKSNPSKRH-39; NLS-1 (human)

63-KLDKLSVLRLS-73; NES-1 (human) 55-75 mouse

214-FRCRLRCLL-222; NES-2 (mouse)/ 220-FICRLRCLL-228 human

V647 indispensable for NES activity; NES-3 (human)

648-VNGMFENWNSNQFVPFNCPQQDPQQ-671; NLS-2 (human)

NPAS4:

lenght 802 mouse

HLH	1-59
PAS-1	59-144
PAS-2	194-340

NLS: bHLH (10-52), PAS-2 x2 (158-191, 285-316?), C-terminus (593-622?)
NES: bHLH (26-45), PAS-2 (227-242), C-terminus x2 (461-580?, 591-600)

10-KARRDQINAEIRNLKELLPLAEADKVRLSYLHIMSLACIYTRK-52; NLS-1
26- LLPLAEADKVRLSYLHIMSL-45; NES-1
158- RRQSAGNKLVLIRGRFHAHPPGAYWAGNPVFTAF-191; NLS-2
227-LALLDISESVLITYLGF-242; NES-2
285-RLQAKH GGWTWIYCMLYSDGPEGPITANNYPI-316; NLS-3?
461-580; NES-3?
591-LAQLRGPLSV- 600; NES-4
593- QLRGPLSVDVPLVPEGLLTPEASPVKQSFF-622; NLS-4?
