# Supplementary material for: Subcellular Localization Signals of bHLH-PAS Proteins: Their Significance, Current State of Knowledge and Future Perspectives
Source: Int J Mol Sci. 2019 Sep 24;20(19):4746. doi: 10.3390/ijms20194746 (PMC6801399; doi:10.3390/ijms20194746)
Supplement: Supplementary file 1 [file ijms-20-04746-s001.zip › SupplementaryIJMS/Supplementary materials1.pdf]

Subcellular Localization Signals of bHLH-PAS Proteins: their Significance, Current State of Knowledge and Future Perspectives

Beata Greb-Markiewicz [1,*] and Marta Kolonko [1]

Supplementary Materials Table 1

Predictors used for NLS prediction:

cNLS Mapper	a
NLStradamus	b
NucPred	c
PSORT II	d
SeqNLS	e
ELM	f

Predictors used for NES prediction:

ELM	f
NES Finder	g
NetNES	h
LocNES	i

hAhR

```
>sp|P35869|AHR_HUMAN Aryl hydrocarbon receptor OS=Homo sapiens OX=9606
GN=AHR PE=1 SV=2
MNSSSANITYASRKRRKPVQKTVKPIPAEGIKSNPSKRHRDRLNTELDRLASLLPFPQDV
INKLDKLSVLRLSVSYLRAKSFFDVALKSSPTERNGGQDNCRAANFREGLNLQEGEFLLQ
ALNGFVLVVTTDALVFYASSTIQDYLGFQQSDVIHQSVYELIHTEDRAEFQRQLHWALNP
SQCTESGQGIEEATGLPQTVVCYNPDQIPPENSPLMERCFICRLRCLLDNSSGFLAMNFQ
GKLKYLHGQKKKGKDGSILPPQLALFAIATPLQPPSILEIRTKNFIFRTKHKLDFTPIGC
DAKGRIVLGYTEAELCTRGSGYQFIHAADMLYCAESHIRMIKTGESGMIVFRLLTKNNRW
TWVQSNARLLYKNGRPDYIIVTQRPLTDEEGTEHLRKRNTKLPFMFTTGEAVLYEATNPF
PAIMDPLPLRTKNGTSGKDSATTSTLSKDSLNPSSLLAAMMQQDESIYLYPASSTSSTAP
FENNFFNESMNECRNWQDNTAPMGNDTILKHEQIDQPQDVNSFAGGHPGLFQDSKNSDLY
SIMKNLGIDFEDIRHMQNEKFFRNDFSGEVDFRDIDLTDEILTYVQDSLSKSPFIPSDYQ
QQQSLALNSSCMVQEHLHLEQQQQHHQKQVVVEPQQQLCQKMKHMQVNGMFENWNSNQFV
PFNCPQQDPQQYNVFTDLHGISQEFPYKSEMDSMPYTQNFISCNQPVLPQHSKCTELDYP
MGSFEPSPYPTTSSLEDFVTCLQLPENQKHGLNPQSAIITPQTCYAGAVSMYQCQPEPQH
THVGQMQYNPVLPGQQAFLNKFQNGVLNETYPAELNNINNTQTTTHLQPLHHPSEARPFP
DLTSSGFL
```

cNLS Mapper Results cut-off score 2.0

Predicted NLSs in query sequence

```
MNSSSANITYASRKRRKPVQKTVKPIPAEGIKSNPSKRHRDRLNTELDRL  50
ASLLPFPQDVINKLDKLSVLRLSVSYLRAKSFFDVALKSSPTERNGGQDN  100
CRAANFREGLNLQEGEFLLQALNGFVLVVTTDALVFYASSTIQDYLGFQQ  150
SDVIHQSVYELIHTEDRAEFQRQLHWALNPSQCTESGQGIEEATGLPQTV  200
VCYNPDQIPPENSPLMERCFICRLRCLLDNSSGFLAMNFQGKLKYLHGQK  250
KKGKDGSILPPQLALFAIATPLQPPSILEIRTKNFIFRTKHKLDFTPIGC  300
DAKGRIVLGYTEAELCTRGSGYQFIHAADMLYCAESHIRMIKTGESGMIV  350
FRLLTKNNRWTWVQSNARLLYKNGRPDYIIVTQRPLTDEEGTEHLRKRNT  400
KLPFMFTTGEAVLYEATNPFPAIMDPLPLRTKNGTSGKDSATTSTLSKDS  450
LNPSSLLAAMMQQDESIYLYPASSTSSTAPFENNFFNESMNECRNWQDNT  500
APMGNDTILKHEQIDQPQDVNSFAGGHPGLFQDSKNSDLYSIMKNLGIDF  550
EDIRHMQNEKFFRNDFSGEVDFRDIDLTDEILTYVQDSLSKSPFIPSDYQ  600
QQQSLALNSSCMVQEHLHLEQQQQHHQKQVVVEPQQQLCQKMKHMQVNGM  650
FENWNSNQFVPFNCPQQDPQQYNVFTDLHGISQEFPYKSEMDSMPYTQNF  700
ISCNQPVLPQHSKCTELDYPMGSFEPSPYPTTSSLEDFVTCLQLPENQKH  750
GLNPQSAIITPQTCYAGAVSMYQCQPEPQHTHVGQMQYNPVLPGQQAFLN  800
KFQNGVLNETYPAELNNINNTQTTTHLQPLHHPSEARPFPDLTSSGFL    848
```

Predicted monopartite NLS

Pos.	Sequence	Score
10	YASRKRRKPV	9
11	ASRKRRKPVQ	11

Predicted bipartite NLS

Pos.	Sequence	Score
13	RKRRKPVQKTVKPIPAEGIKSNPSKRH	4.4
13	RKRRKPVQKTVKPIPAEGIKSNPSKRHRD	5.4
13	RKRRKPVQKTVKPIPAEGIKSNPSKRHRDR	2.4
13	RKRRKPVQKTVKPIPAEGIKSNPSKRHRDR	3.4
13	RKRRKPVQKTVKPIPAEGIKSNPSKRHRDR	2
13	RKRRKPVQKTVKPIPAEGIKSNPSKRHRDRL	3.7
13	RKRRKPVQKTVKPIPAEGIKSNPSKRHRDR	3.2
13	RKRRKPVQKTVKPIPAEGIKSNPSKRHRDRLNTELD	2.3
34	NPSKRHRDRLNTELDRLASLLPFPQDVINK	2.9
34	NPSKRHRDRLNTELDRLASLLPFPQDVIN	4.9
37	KRHRDRLNTELDRLASLLPFPQDVINKLD	2.5
38	RHRDRLNTELDRLASLLPFPQDVINKLDKLS	2.3
63	KLDKLSVLRLSVSYLRAKSFFDVALKSSPTE	2
78	RAKSFFDVALKSSPTERNGGQDNCRAANF	3
218	RCFICRLRCLLDNSSGFLAMNFQGKLKYLHG	2
218	RCFICRLRCLLDNSSGFLAMNFQGKLKYLHGQK	2.2
239	FQGKLKYLHGQKKKGKDGSILPPQLALFAI	2.2
241	GKLKYLHGQKKKGKDGSILPPQLALFAIATP	2.1
248	GQKKKGKDGSILPPQLALFAIATPLQPPSI	2.3
250	KKKGKDGSILPPQLALFAIATPLQPPSILEI	3.1
279	EIRTKNFIFRTKHKLDFTPIGCDAKGRIVLGY	2.2
368	RLLYKNGRPDYIIVTQRPLTDEEGTEHLRKR	3.7
368	RLLYKNGRPDYIIVTQRPLTDEEGTEHLRKRNT	3.5
393	EHLRKRNTKLPFMFTTGEAVLYEATNPFPAI	3.4
396	RKRNTKLPFMFTTGEAVLYEATNPFPAIMDPLP	3
640	QKMKHMQVNGMFENWNSNQFVPFNCPQQD	2.7

643	KHMQVNGMFENWNSNQFVPFNCPQQDP	2.5
684	EFPYKSEMDSMPYTQNFISCNQPVLPQHSKCT	2.1
746	ENQKHGLNPQSAIITPQTCYAGAVSMYQCQP	2.9

NLStradamus

Cut-off 0.3 4 state static

12 - SRKRRKPVQKTVKPIPAEGIKSNPSKRHRDR - 42

247 - HGQKKKGK – 254

NucPred

The NucPred score for your sequence is 0.94 (see score help below)

```
  1  MNSSSANITYASRKRRKPVQKTVKPIPAEGIKSNPSKRHRDRLNTELDRL    50
 51  ASLLPFPQDVINKLDKLSVLRLSVSYLRAKSFFDVALKSSPTERNGGQDN   100
101  CRAANFREGLNLQEGEFLLQALNGFVLVVTTDALVFYASSTIQDYLGFQQ   150
151  SDVIHQSVYELIHTEDRAEFQRQLHWALNPSQCTESGQGIEEATGLPQTV   200
201  VCYNPDQIPPENSPLMERCFICRLRCLLDNSSGFLAMNFQGKLKYLHGQK   250
251  KKGKDGSILPPQLALFAIATPLQPPSILEIRTKNFIFRTKHKLDFTPIGC   300
301  DAKGRIVLGYTEAELCTRGSGYQFIHAADMLYCAESHIRMIKTGESGMIV   350
351  FRLLTKNNRWTWVQSNARLLYKNGRPDYIIVTQRPLTDEEGTEHLRKRNT   400
401  KLPFMFTTGEAVLYEATNPFPAIMDPLPLRTKNGTSGKDSATTSTLSKDS   450
451  LNPSSLLAAMMQQDESIYLYPASSTSSTAPFENNFFNESMNECRNWQDNT   500
501  APMGNDTILKHEQIDQPQDVNSFAGGHPGLFQDSKNSDLYSIMKNLGIDF   550
551  EDIRHMQNEKFFRNDFSGEVDFRDIDLTDEILTYVQDSLSKSPFIPSDYQ   600
601  QQQSLALNSSCMVQEHLHLEQQQQHHQKQVVVEPQQQLCQKMKHMQVNGM   650
651  FENWNSNQFVPFNCPQQDPQQYNVFTDLHGISQEFPYKSEMDSMPYTQNF   700
701  ISCNQPVLPQHSKCTELDYPMGSFEPSPYPTTSSLEDFVTCLQLPENQKH   750
751  GLNPQSAIITPQTCYAGAVSMYQCQPEPQHTHVGQMQYNPVLPGQQAFLN   800
801  KFQNGVLNETYPAELNNINNTQTTTHLQPLHHPSEARPFPDLTSSGFL     848
```

Positively and negatively influencing subsequences are coloured according to the following scale:

(non-nuclear) negative |||||||||||||||||||||||||||||||||||||||||||||||||||||||||||||||| positive (nuclear)

PSORTII

NUCDISC: discrimination of nuclear localization signals

```
        pat4: RKRR (5) at   13
        pat4: KRRK (5) at   14
        pat4: RRKP (4) at   15
        pat4: KRHR (3) at   37
        pat7: PSKRHRD (4) at   35
        bipartite: none
        content of basic residues:   8.8%
        NLS Score:  0.94
```

SeqNLS

Prediction result (The predicted NLS(s) are underlined)

AhR: MNSSSANITYASRKRRKPVQKTVKPIPAEGIKSNPSKRHRDRLNTELDRLASLLPFPQDV

Definition of different colors in predictions

score range	color
0.1-0.3	
0.3-0.5	
0.5-0.7	
0.7-0.8	
0.8-0.86	
0.86-0.89	
>0.89	

The predicted NLS(s) (score cutoff = 0.5)

Protein ID	Predicted NLS	Start	Stop	The highest score of matches within the prediction
AhR	ASRKRRKPVQKTVK	11	24	0.887
AhR	PAEGIKSNPSKRHRDRL	27	43	0.886

ELM

Motif Probability Cutoff:100

TRG_NLS_MonoCore_2 2.345e-04

SRKRRK 12-17

RKRRKP 13-18

Monopartite variant of the classical basically charged NLS. Strong core version.

TRG_NLS_MonoExtC_3 7.252e-04

SRKRRK 12-17

RKRRKP 13-18

QKKKGK 249-254

Monopartite variant of the classical basically charged NLS. C-extended version.

TRG_NLS_MonoExtN_4 1.276e-03

RKRRKP 13-18

Monopartite variant of the classical basically charged NLS. N-extended version.

TRG_NES_CRM1_1   7.626e-04

DLYSIMKNLGIDFED   538-552

NES Finder

Position   NES   Type

==============================

47   LDRLASLLPF   2-3-1

60   VINKLDKLSV   3-2-1

63   KLDKLSVLRL   2-2-1

259   LPPQLALFAI   3-2-1

420   FPAIMDPLPL   3-2-1

539   LYSIMKNLGI   3-2-1

735   LEDFVTCLQL   3-2-1

LocNES

Protein Name	Position	Sequence	Score
>LocNES1759629158_0	46-60	RLNTELDRLASLLPF	0.394
>LocNES1759629158_0	59-73	PFPQDVINKLDKLSV	0.746
>LocNES1759629158_0	62-76	QDVINKLDKLSVLRL	0.658
>LocNES1759629158_0	64-78	VINKLDKLSVLRLSV	0.444
>LocNES1759629158_0	67-81	KLDKLSVLRLSVSYL	0.452
>LocNES1759629158_0	77-91	SVSYLRAKSFFDVAL	0.026
>LocNES1759629158_0	102-116	QDNCRAANFREGLNL	0.043
>LocNES1759629158_0	117-131	QEGEFLLQALNGFVL	0.053
>LocNES1759629158_0	118-132	EGEFLLQALNGFVLV	0.081
>LocNES1759629158_0	119-133	GEFLLQALNGFVLVV	0.055
>LocNES1759629158_0	126-140	LNGFVLVVTTDALVF	0.043
>LocNES1759629158_0	138-152	LVFYASSTIQDYLGF	0.015
>LocNES1759629158_0	236-250	SGFLAMNFQGKLKYL	0.045
>LocNES1759629158_0	258-272	KDGSILPPQLALFAI	0.138
>LocNES1759629158_0	277-291	QPPSILEIRTKNFIF	0.013
>LocNES1759629158_0	285-299	RTKNFIFRTKHKLDF	0.017
>LocNES1759629158_0	298-312	DFTPIGCDAKGRIVL	0.027
>LocNES1759629158_0	330-344	HAADMLYCAESHIRM	0.013

>LocNES1759629158_0	331-345	AADMLYCAESHIRMI	0.022
>LocNES1759629158_0	413-427	GEAVLYEATNPFPAI	0.021
>LocNES1759629158_0	417-431	LYEATNPFPAIMDPL	0.118
>LocNES1759629158_0	419-433	EATNPFPAIMDPLPL	0.083
>LocNES1759629158_0	538-552	SKNSDLYSIMKNLGI	0.070
>LocNES1759629158_0	543-557	LYSIMKNLGIDFEDI	0.043
>LocNES1759629158_0	562-576	NEKFFRNDFSGEVDF	0.005
>LocNES1759629158_0	567-581	RNDFSGEVDFRDIDL	0.020
>LocNES1759629158_0	575-589	DFRDIDLTDEILTYV	0.029
>LocNES1759629158_0	637-651	EPQQQLCQKMKHMQV	0.026
>LocNES1759629158_0	732-746	PYPTTSSLEDFVTCL	0.174
>LocNES1759629158_0	734-748	PTTSSLEDFVTCLQL	0.128

NetNES 1.1 Server prediction results

>Sequence - NetNES 1.1 prediction

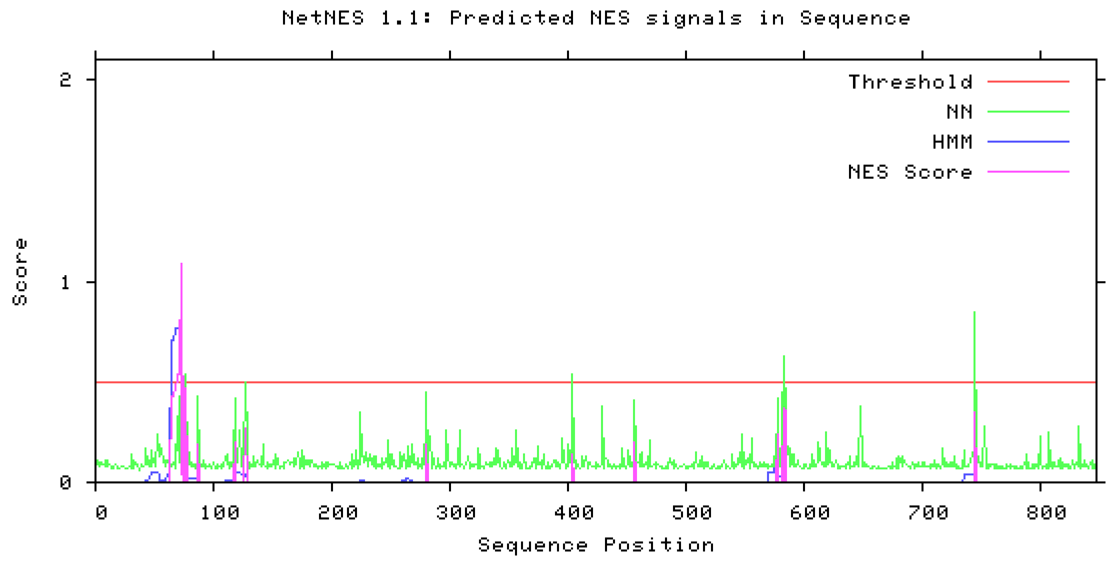

hHIF1α

>sp|Q16665|HIF1A_HUMAN Hypoxia-inducible factor 1-alpha OS=Homo sapiens OX=9606 GN=HIF1A PE=1 SV=1
MEGAGGANDKKKISSERRKEKSRDAARSRRSKESEVFYELAHQLPLPHNVSSHLDKASVM

RLTISYLRVRKLLDAGDLDIEDDMKAQMNCFYLKALDGFVMVLTDDGDMIYISDNVNKYM
GLTQFELTGHSVFDFTHPCDHEEMREMLTHRNGLVKKGKEQNTQRSFFLRMKCTLTSRGR
TMNIKSATWKVLHCTGHIHVYDTNSNQPQCGYKKPPMTCLVLICEPIPHPSNIEIPLDSK
TFLSRHSLDMKFSYCDERITELMGYEPEELLGRSIYEYYHALDSDHLTKTHHDMFTKGQV
TTGQYRMLAKRGGYVWVETQATVIYNTKNSQPQCIVCVNYVVSGIIQHDLIFSLQQTECV
LKPVESSDMKMTQLFTKVESEDTSSLFDKLKKEPDALTLLAPAAGDTIISLDFGSNDTET
DDQQLEEVPLYNDVMLPSPNEKLQNINLAMSPLPTAETPKPLRSSADPALNQEVALKLEP
NPESLELSFTMPQIQDQTPSPSDGSTRQSSPEPNSPSEYCFYVDSDMVNEFKLELVEKLF
AEDTEAKNPFSTQDTDLDLEMLAPYIPMDDDFQLRSFDQLSPLESSSASPESASPQSTVT
VFQQTQIQEPTANATTTTATTDELKTVTKDRMEDIKILIASPSPTHIHKETTSATSSPYR
DTQSRTASPNRAGKGVIEQTEKSHPRSPNVLSVALSQRTTVPEEELNPKILALQNAQRKR
KMEHDGSLFQAVGIGTLLQQPDDHAATTSLSWKRVKGCKSSEQNGMEQKTIILIPSDLAC
RLLGQSMDESGLPQLTSYDCEVNAPIQGSRNLLQGEELLRALDQVN

cNLS Mapper Result cut-off score 2.0

Predicted NLSs in query sequence	
MEGAGGANDKKKISSERRKEKSRDAARSRRSKESEVFYELAHQLPLPHNV	50
SSHLDKASVMRLTISYLRVRKLLDAGDLDIEDDMKAQMNCFYLKALDGFV	100
MVLTDDGDMIYISDNVNKYMGLTQFELTGHSVFDFTHPCDHEEMREMLTH	150
RNGLVKKGKEQNTQRSFFLRMKCTLTSRGRTMNIKSATWKVLHCTGHIHV	200
YDTNSNQPQCGYKKPPMTCLVLICEPIPHPSNIEIPLDSKTFLSRHSLDM	250
KFSYCDERITELMGYEPEELLGRSIYEYYHALDSDHLTKTHHDMFTKGQV	300
TTGQYRMLAKRGGYVWVETQATVIYNTKNSQPQCIVCVNYVVSGIIQHDL	350
IFSLQQTECVLKPVESSDMKMTQLFTKVESEDTSSLFDKLKKEPDALTLL	400
APAAGDTIISLDFGSNDTETDDQQLEEVPLYNDVMLPSPNEKLQNINLAM	450
SPLPTAETPKPLRSSADPALNQEVALKLEPNPESLELSFTMPQIQDQTPS	500
PSDGSTRQSSPEPNSPSEYCFYVDSDMVNEFKLELVEKLFAEDTEAKNPF	550
STQDTDLDLEMLAPYIPMDDDFQLRSFDQLSPLESSSASPESASPQSTVT	600
VFQQTQIQEPTANATTTTATTDELKTVTKDRMEDIKILIASPSPTHIHKE	650
TTSATSSPYRDTQSRTASPNRAGKGVIEQTEKSHPRSPNVLSVALSQRTT	700
VPEEELNPKILALQNAQRKRKMEHDGSLFQAVGIGTLLQQPDDHAATTSL	750
SWKRVKGCKSSEQNGMEQKTIILIPSDLACRLLGQSMDESGLPQLTSYDC	800
EVNAPIQGSRNLLQGEELLRALDQVN	826

Predicted monopartite NLS

Pos.	Sequence	Score
716	AQRKRKMEHD	8

Predicted bipartite NLS

Pos.	Sequence	Score
8	NDKKKISSERRKEKSRDAARSRRSKESEV	2.6
23	RDAARSRRSKESEVFYELAHQLPLPHNVSSHLDK	2.4
68	RVRKLLDAGDLDIEDDMKAQMNCFYLKALD	2.6
68	RVRKLLDAGDLDIEDDMKAQMNCFYLKALD	2.9
189	WKVLHCTGHIHVYDTNSNQPQCGYKKPP	2.6
211	GYKKPPMTCLVLICEPIPHPSNIEIPLDSKT	2.1

245	RHSLDMKFSYCDERITELMGYEPEELLGRSIYEY	2.8
285	DHLTKTHHDMFTKGQVTTGQYRMLAKRGGY	2.3
303	GQYRMLAKRGGYVWVETQATVIYNTKNSQP	2.4
306	RMLAKRGGYVWVETQATVIYNTKNSQPQCIV	2.3
365	ESSDMKMTQLFTKVESEDTSSLFDKLKKEPDA	2.7
647	IHKETTSATSSPYRDTQSRTASPNRAGKG	2.4
697	QRTTVPEEELNPKILALQNAQRKRKME	2.6
697	QRTTVPEEELNPKILALQNAQRKRKMEHD	2.7
717	QRKRKMEHDGSLFQAVGIGTLLQQPDDHA	3.2

NLStradamus

cutoff 0.1 4 state HMM

3 - GAGGANDKKKISSERRKEKSRDAARSRRSKESEVFYELA - 41

155 - VKKGKE - 160

709 - KILALQNAQRKRKME – 723

NucPred

The NucPred score for your sequence is 0.94 (see score help below)

1	MEGAGGANDKKKISSERRKEKSRDAARSRRSKESEVFYELAHQLPLPHNV	50
51	SSHLDKASVMRLTISYLRVRKLLDAGDLDIEDDMKAQMNCFYLKALDGFV	100
101	MVLTDDGDMIYISDNVNKYMGLTQFELTGHSVFDFTHPCDHEEMREMLTH	150
151	RNGLVKKGKEQNTQRSFFLRMKCTLTSRGRTMNIKSATWKVLHCTGHIHV	200
201	YDTNSNQPQCGYKKPPMTCLVLICEPIPHPSNIEIPLDSKTFLSRHSLDM	250
251	KFSYCDERITELMGYEPEELLGRSIYEYYHALDSDHLTKTHHDMFTKGQV	300
301	TTGQYRMLAKRGGYVWVETQATVIYNTKNSQPQCIVCVNYVVSGIIQHDL	350
351	IFSLQQTECVLKPVESSDMKMTQLFTKVESEDTSSLFDKLKKEPDALTLL	400
401	APAAGDTIISLDFGSNDTETDDQQLEEVPLYNDVMLPSPNEKLQNINLAM	450
451	SPLPTAETPKPLRSSADPALNQEVALKLEPNPESLELSFTMPQIQDQTPS	500
501	PSDGSTRQSSPEPNSPSEYCFYVDSDMVNEFKLELVEKLFAEDTEAKNPF	550
551	STQDTDLDLEMLAPYIPMDDDFQLRSFDQLSPLESSSASPESASPQSTVT	600
601	VFQQTQIQEPTANATTTTATTDELKTVTKDRMEDIKILIASPSPTHIHKE	650
651	TTSATSSPYRDTQSRTASPNRAGKGVIEQTEKSHPRSPNVLSVALSQRTT	700
701	VPEEELNPKILALQNAQRKRKMEHDGSLFQAVGIGTLLQQPDDHAATTSL	750
751	SWKRVKGCKSSEQNGMEQKTIILIPSDLACRLLGQSMDESGLPQLTSYDC	800
801	EVNAPIQGSRNLLQGEELLRALDQVN	826

Positively and negatively influencing subsequences are coloured according to the following scale:

(non-nuclear) negative positive (nuclear)

PSORTII

NUCDISC: discrimination of nuclear localization signals

```
pat4: RKRK (5) at  718
pat7: none
bipartite: RRKEKSRDAARSRRSKE at   17
content of basic residues:  10.3%
NLS Score:  0.33
```

SeqNLS

Prediction result (The predicted NLS(s) are underlined)

Hif1a: MEGAGGANDKKKISSERRKEKSRDAARSRRSKESEVFYELAHQLPLPHNVSSHLDKASVM

The predicted NLS(s) (score cutoff = 0.5)

Protein ID	Predicted NLS	Start	Stop	The highest score of matches within the prediction
Hif1a	KKKISSERRKEKSRDAARSRRSKE	10	33	0.964
Hif1a	SVM	58	60	0.696
Hif1a	SEVFYE	34	39	0.568

ELM

TRG_NLS_MonoCore_2 2.345e-04

QRKRKM 717-722

Monopartite variant of the classical basically charged NLS. Strong core version.

TRG_NLS_MonoExtC_3 7.252e-04

QRKRKME 717-723

Monopartite variant of the classical basically charged NLS. C-extended version.

TRG_NES_CRM1_1 7.626e-04

DDMKAQMNCFYLKALD 82-97 ;

DMKAQMNCFYLKALD 83-97

Some proteins re-exported from the nucleus contain a Leucine-rich nuclear export signal (NES) binding to the CRM1 exportin protein.

NES Finder

Position	NES	Type
37	FYELAHQLPL	2-3-1
60	MRLTISYLRV	3-2-1
84	MKAQMNCFYL	3-2-1
559	LEMLAPYIPM	2-3-1

LocNES

Protein Name	Position	Sequence	Score
>LocNES223789098_0	36-50	KESEVFYELAHQLPL	0.072
>LocNES223789098_0	52-66	HNVSSHLDKASVMRL	0.122
>LocNES223789098_0	54-68	VSSHLDKASVMRLTI	0.026
>LocNES223789098_0	57-71	HLDKASVMRLTISYL	0.090
>LocNES223789098_0	59-73	DKASVMRLTISYLRV	0.110
>LocNES223789098_0	70-84	YLRVRKLLDAGDLDI	0.103
>LocNES223789098_0	83-97	DIEDDMKAQMNCFYL	0.046
>LocNES223789098_0	91-105	QMNCFYLKALDGFVM	0.022
>LocNES223789098_0	92-106	MNCFYLKALDGFVMV	0.012
>LocNES223789098_0	93-107	NCFYLKALDGFVMVL	0.011
>LocNES223789098_0	112-126	DMIYISDNVNKYMGL	0.035
>LocNES223789098_0	117-131	SDNVNKYMGLTQFEL	0.027
>LocNES223789098_0	125-139	GLTQFELTGHSVFDF	0.009
>LocNES223789098_0	190-204	SATWKVLHCTGHIHV	0.011
>LocNES223789098_0	335-349	QPQCIVCVNYVVSGI	0.019
>LocNES223789098_0	342-356	VNYVVSGIIQHDLIF	0.066
>LocNES223789098_0	354-368	LIFSLQQTECVLKPV	0.012
>LocNES223789098_0	403-417	LLAPAAGDTIISLDF	0.028
>LocNES223789098_0	443-457	PNEKLQNINLAMSPL	0.284
>LocNES223789098_0	466-480	LRSSADPALNQEVAL	0.114
>LocNES223789098_0	523-537	YCFYVDSDMVNEFKL	0.065
>LocNES223789098_0	558-572	DTDLDLEMLAPYIPM	0.190
>LocNES223789098_0	627-641	ELKTVTKDRMEDIKI	0.365

NetNES 1.1 Server - prediction results

>sp_Q16665_HIF1A_HUMAN - NetNES 1.1 prediction

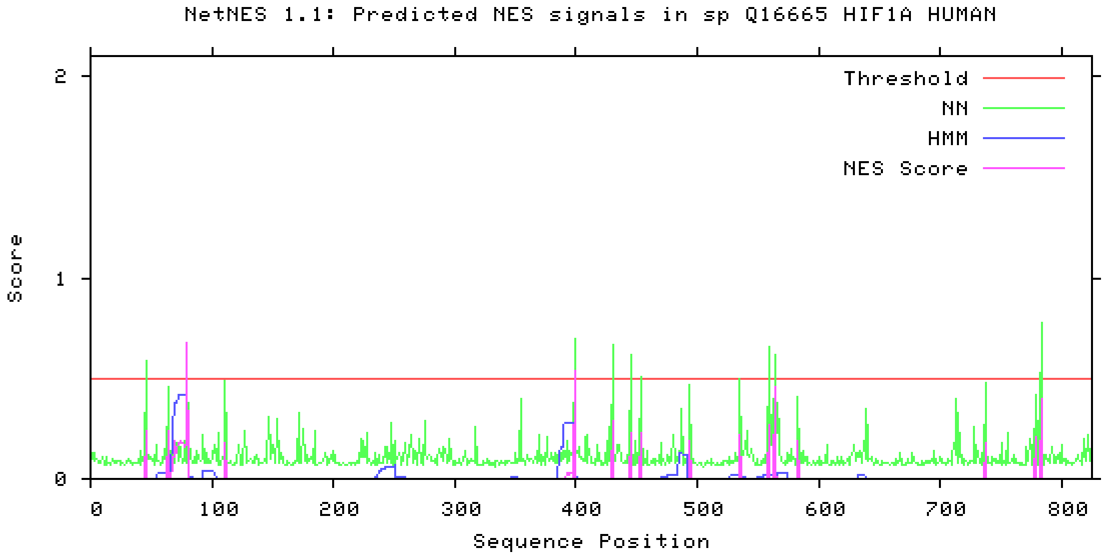

#

hHIF2α

>sp|Q99814|EPAS1_HUMAN Endothelial PAS domain-containing protein 1 OS=Homo sapiens OX=9606 GN=EPAS1 PE=1 SV=3
MTADKEKKRSSSERRKEKSRDAARCRRSKETEVFYELAHELPLPHSVSSHLDKASIMRLA
ISFLRTHKLLSSVCSENESEAEADQQMDNLYLKALEGFIAVVTQDGDMIFLSENISKFMG
LTQVELTGHSIFDFTHPCDHEEIRENLSLKNGSGFGKKSKDMSTERDFFMRMKCTVTNRG

```
RTVNLKSATWKVLHCTGQVKVYNNCPPHNSLCGYKEPLLSCLIIMCEPIQHPSHMDIPLD
SKTFLSRHSMDMKFTYCDDRITELIGYHPEELLGRSAYEFYHALDSENMTKSHQNLCTKG
QVVSGQYRMLAKHGGYVWLETQGTVIYNPRNLQPQCIMCVNYVLSEIEKNDVVFSMDQTE
SLFKPHLMAMNSIFDSSGKGAVSEKSNFLFTKLKEEPEELAQLAPTPGDAIISLDFGNQN
FEESSAYGKAILPPSQPWATELRSHSTQSEAGSLPAFTVPQAAAPGSTTPSATSSSSSCS
TPNSPEDYYTSLDNDLKIEVIEKLFAMDTEAKDQCSTQTDFNELDLETLAPYIPMDGEDF
QLSPICPEERLLAENPQSTPQHCFSAMTNIFQPLAPVAPHSPFLLDKFQQQLESKKTEPE
HRPMSSIFFDAGSKASLPPCCGQASTPLSSMGGRSNTQWPPDPPLHFGPTKWAVGDQRTE
FLGAAPLGPPVSPPHVSTFKTRSAKGFGARGPDVLSPAMVALSNKLKLKRQLEYEEQAFQ
DLSGGDPPGGSTSHLMWKRMKNLRGGSCPLMPDKPLSANVPNDKFTQNPMRGLGHPLRHL
PLPQPPSAISPGENSKSRFPPQCYATQYQDYSLSSAHKVSGMASRLLGPSFESYLLPELT
RYDCEVNVPVLGSSTLLQGGDLLRALDQAT
```

cNLS Mapper Result cut-off score 2.0

Predicted NLSs in query sequence

```
MTADKEKKRSSSERRKEKSRDAARCRRSKETEVFYELAHELPLPHSVSSH  50
LDKASIMRLAISFLRTHKLLSSVCSENESEAEADQQMDNLYLKALEGFIA  100
VVTQDGDMIFLSENISKFMGLTQVELTGHSIFDFTHPCDHEEIRENLSLK  150
NGSGFGKKSKDMSTERDFFMRMKCTVTNRGRTVNLKSATWKVLHCTGQVK  200
VYNNCPPHNSLCGYKEPLLSCLIIMCEPIQHPSHMDIPLDSKTFLSRHSM  250
DMKFTYCDDRITELIGYHPEELLGRSAYEFYHALDSENMTKSHQNLCTKG  300
QVVSGQYRMLAKHGGYVWLETQGTVIYNPRNLQPQCIMCVNYVLSEIEKN  350
DVVFSMDQTESLFKPHLMAMNSIFDSSGKGAVSEKSNFLFTKLKEEPEEL  400
AQLAPTPGDAIISLDFGNQNFEESSAYGKAILPPSQPWATELRSHSTQSE  450
AGSLPAFTVPQAAAPGSTTPSATSSSSSCSTPNSPEDYYTSLDNDLKIEV  500
IEKLFAMDTEAKDQCSTQTDFNELDLETLAPYIPMDGEDFQLSPICPEER  550
LLAENPQSTPQHCFSAMTNIFQPLAPVAPHSPFLLDKFQQQLESKKTEPE  600
HRPMSSIFFDAGSKASLPPCCGQASTPLSSMGGRSNTQWPPDPPLHFGPT  650
KWAVGDQRTEFLGAAPLGPPVSPPHVSTFKTRSAKGFGARGPDVLSPAMV  700
ALSNKLKLKRQLEYEEQAFQDLSGGDPPGGSTSHLMWKRMKNLRGGSCPL  750
MPDKPLSANVPNDKFTQNPMRGLGHPLRHLPLPQPPSAISPGENSKSRFP  800
PQCYATQYQDYSLSSAHKVSGMASRLLGPSFESYLLPELTRYDCEVNVPV  850
LGSSTLLQGGDLLRALDQAT                                870
```

Predicted monopartite NLS

Pos.	Sequence	Score

Predicted bipartite NLS

Pos.	Sequence	Score
3	ADKEKKRSSSERRKEKSRDAARCRRSKETE	2
5	KEKKRSSSERRKEKSRDAARCRRSKETEV	2.5
6	EKKRSSSERRKEKSRDAARCRRSKETEV	2.1
20	RDAARCRRSKETEVFYELAHELPLPHSVSSH	2
20	RDAARCRRSKETEVFYELAHELPLPHSVSSHLDK	2.6
24	RCRRSKETEVFYELAHELPLPHSVSSHLDKASI	2
65	RTHKLLSSVCSENESEAEADQQMDNLYLKA	3.8
144	RENLSLKNGSGFGKKSKDMSTERDFFMRMKCT	2.2

144	RENLSLKNGSGFGKKSKDMSTERDFFMRMKCTVT	2.9
179	RGRTVNLKSATWKVLHCTGQVKVYNNCPPHNSLC	2.2
197	GQVKVYNNCPPHNSLCGYKEPLLSCLIIMCE	2.5
247	RHSMDMKFTYCDDRITELIGYHPEELLGRSAYE	2.3
360	ESLFKPHLMAMNSIFDSSGKGAVSEKSNFLFT	2
610	DAGSKASLPPCCGQASTPLSSMGGRSNTQWPP	2.4
682	RSAKGFGARGPDVLSPAMVALSNKLKLKR	4.1
704	NKLKLKRQLEYEEQAFQDLSGGDPPGGST	2.6
705	KLKLKRQLEYEEQAFQDLSGGDPPGGSTSH	4.2
705	KLKLKRQLEYEEQAFQDLSGGDPPGGSTSHL	5.4
737	WKRMKNLRGGSCPLMPDKPLSANVPNDKFT	3.2
752	PDKPLSANVPNDKFTQNPMRGLGHPLRHLP	2.5
793	ENSKSRFPPQCYATQYQDYSLSSAHKVSGM	4
817	HKVSGMASRLLGPSFESYLLPELTRYDCE	3.2

NLStradamus

0.1 cut-off 4 state HMM static

3 - ADKEKKRSSSERRKEKSRDAARCRRSKETEV - 33

156 - GKKSK - 160

680 - KTRSAKGFGARGPDVLSPAMVALSNKLKLKR - 710

738 - KRMK - 741

NucPred

The NucPred score for your sequence is 0.86 (see score help below)

```
  1   MTADKEKKRSSSERRKEKSRDAARCRRSKETEVFYELAHELPLPHSVSSH    50
 51   LDKASIMRLAISFLRTHKLLSSVCSENESEAEADQQMDNLYLKALEGFIA   100
101   VVTQDGDMIFLSENISKFMGLTQVELTGHSIFDFTHPCDHEEIRENLSLK   150
151   NGSGFGKKSKDMSTERDFFMRMKCTVTNRGRTVNLKSATWKVLHCTGQVK   200
201   VYNNCPPHNSLCGYKEPLLSCLIIMCEPIQHPSHMDIPLDSKTFLSRHSM   250
251   DMKFTYCDDRITELIGYHPEELLGRSAYEFYHALDSENMTKSHQNLCTKG   300
301   QVVSGQYRMLAKHGGYVWLETQGTVIYNPRNLQPQCIMCVNYVLSEIEKN   350
351   DVVFSMDQTESLFKPHLMAMNSIFDSSGKGAVSEKSNFLFTKLKEEPEEL   400
401   AQLAPTPGDAIISLDFGNQNFEESSAYGKAILPPSQPWATELRSHSTQSE   450
451   AGSLPAFTVPQAAAPGSTTPSATSSSSSCSTPNSPEDYYTSLDNDLKIEV   500
501   IEKLFAMDTEAKDQCSTQTDFNELDLETLAPYIPMDGEDFQLSPICPEER   550
551   LLAENPQSTPQHCFSAMTNIFQPLAPVAPHSPFLLDKFQQQLESKKTEPE   600
601   HRPMSSIFFDAGSKASLPPCCGQASTPLSSMGGRSNTQWPPDPPLHFGPT   650
651   KWAVGDQRTEFLGAAPLGPPVSPPHVSTFKTRSAKGFGARGPDVLSPAMV   700
701   ALSNKLKLKRQLEYEEQAFQDLSGGDPPGGSTSHLMWKRMKNLRGGSCPL   750
751   MPDKPLSANVPNDKFTQNPMRGLGHPLRHLPLPQPPSAISPGENSKSRFP   800
801   PQCYATQYQDYSLSSAHKVSGMASRLLGPSFESYLLPELTRYDCEVNVPV   850
851   LGSSTLLQGGDLLRALDQAT                                 870
```

Positively and negatively influencing subsequences are coloured according to the following scale:

(non-nuclear) negative positive (nuclear)

PSORTII

```
NUCDISC: discrimination of nuclear localization signals
      pat4: none
      pat7: none
      bipartite: RRKEKSRDAARCRRSKE at   14
      content of basic residues:   9.8%
      NLS Score:  0.02
```

Seq NLS

Prediction result (The predicted NLS(s) are underlined)

Hif2a: MTADKEK<u>KRSSSE</u><u>RRKEKSRDAARCRRSKE</u>TEV<u>FYELAHE</u>LPLPHSVSSHLDKASIMRLA

The predicted NLS(s) (score cutoff = 0.5)

Protein ID	Predicted NLS	Start	Stop	The highest score of matches within the prediction
Hif2a	KRSSSERRKEKSRDAARCRRSKE	8	30	0.916
Hif2a	FYELAHE	34	40	0.844

ELM

Motif Probability Cutoff:100 No NLS and NES positive result

NES Finder

Position NES Type

==============================

34 FYELAHELPL 2-3-1

92 LKALEGFIAV 2-3-1

498 IEVIEKLFAM 2-3-1

526 LETLAPYIPM 2-3-1

699 MVALSNKLKL 2-3-1

773 LGHPLRHLPL 3-2-1

LocNES

Protein Name	Position	Sequence	Score
>LocNES1396965342_0	33-47	KETEVFYELAHELPL	0.135
>LocNES1396965342_0	49-63	HSVSSHLDKASIMRL	0.190
>LocNES1396965342_0	51-65	VSSHLDKASIMRLAI	0.078

>LocNES1396965342_0	53-67	SHLDKASIMRLAISF	0.084
>LocNES1396965342_0	54-68	HLDKASIMRLAISFL	0.171
>LocNES1396965342_0	63-77	LAISFLRTHKLLSSV	0.047
>LocNES1396965342_0	82-96	ESEAEADQQMDNLYL	0.158
>LocNES1396965342_0	91-105	MDNLYLKALEGFIAV	0.020
>LocNES1396965342_0	92-106	DNLYLKALEGFIAVV	0.011
>LocNES1396965342_0	108-122	QDGDMIFLSENISKF	0.043
>LocNES1396965342_0	111-125	DMIFLSENISKFMGL	0.059
>LocNES1396965342_0	116-130	SENISKFMGLTQVEL	0.105
>LocNES1396965342_0	124-138	GLTQVELTGHSIFDF	0.014
>LocNES1396965342_0	191-205	SATWKVLHCTGQVKV	0.011
>LocNES1396965342_0	337-351	QPQCIMCVNYVLSEI	0.045
>LocNES1396965342_0	449-463	HSTQSEAGSLPAFTV	0.032
>LocNES1396965342_0	497-511	DNDLKIEVIEKLFAM	0.234
>LocNES1396965342_0	516-530	KDQCSTQTDFNELDL	0.203
>LocNES1396965342_0	525-539	FNELDLETLAPYIPM	0.038
>LocNES1396965342_0	564-578	PQHCFSAMTNIFQPL	0.124
>LocNES1396965342_0	567-581	CFSAMTNIFQPLAPV	0.008
>LocNES1396965342_0	698-712	VLSPAMVALSNKLKL	0.354
>LocNES1396965342_0	770-784	TQNPMRGLGHPLRHL	0.056
>LocNES1396965342_0	772-786	NPMRGLGHPLRHLPL	0.166

NetNES 1.1 Server - prediction results

>Sequence - NetNES 1.1 prediction

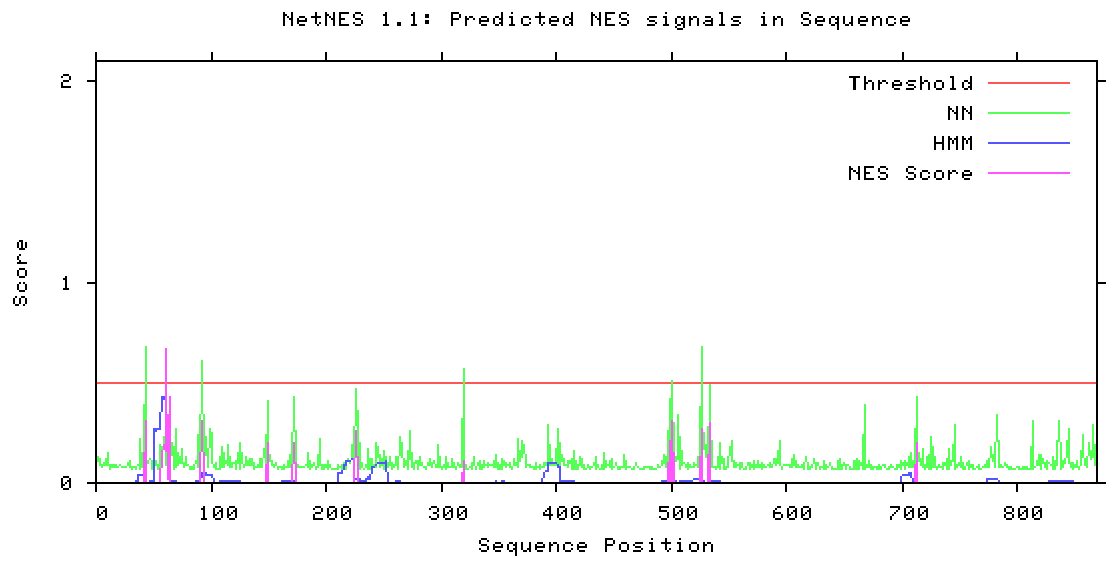

hHIF3α

```
>sp|Q9Y2N7|HIF3A_HUMAN Hypoxia-inducible factor 3-alpha OS=Homo sapiens
OX=9606 GN=HIF3A PE=1 SV=2
MALGLQRARSTTELRKEKSRDAARSRRSQETEVLYQLAHTLPFARGVSAHLDKASIMRLT
ISYLRMHRLCAAGEWNQVGAGGEPLDACYLKALEGFVMVLTAEGDMAYLSENVSKHLGLS
QLELIGHSIFDFIHPCDQEELQDALTPQQTLSRRKVEAPTERCFSLRMKSTLTSRGRTLN
LKAATWKVLNCSGHMRAYKPPAQTSPAGSPDSEPPLQCLVLICEAIPHPGSLEPPLGRGA
FLSRHSLDMKFTYCDDRIAEVAGYSPDDLIGCSAYEYIHALDSDAVSKSIHTLLSKGQAV
TGQYRFLARSGGYLWTQTQATVVSGGRGPQSESIVCVHFLISQVEETGVVLSLEQTEQHS
RRPIQRGAPSQKDTPNPGDSLDTPGPRILAFLHPPSLSEAALAADPRRFCSPDLRRLLGP
ILDGASVAATPSTPLATRHPQSPLSADLPDELPVGTENVHRLFTSGKDTEAVETDLDIAQ
DADALDLEMLAPYISMDDDFQLNASEQLPRAYHRPLGAVPRPRARSFHGLSPPALEPSLL
PRWGSDPRLSCSSPSRGDPSASSPMAGARKRTLAQSSEDEDEGVELLGVRPPKRSPSPEH
ENFLLFPLSLSFLLTGGPAPGSLQDPSTPLLNLNEPLGLGPSLLSPYSDEDTTQPGGPFQ
PRAGSAQAD
```

cNLS Mapper Result cut-off 2.0

Predicted NLSs in query sequence

```
MALGLQRARSTTELRKEKSRDAARSRRSQETEVLYQLAHTLPFARGVSAH  50
LDKASIMRLTISYLRMHRLCAAGEWNQVGAGGEPLDACYLKALEGFVMVL 100
TAEGDMAYLSENVSKHLGLSQLELIGHSIFDFIHPCDQEELQDALTPQQT 150
LSRRKVEAPTERCFSLRMKSTLTSRGRTLNLKAATWKVLNCSGHMRAYKP 200
PAQTSPAGSPDSEPPLQCLVLICEAIPHPGSLEPPLGRGAFLSRHSLDMK 250
FTYCDDRIAEVAGYSPDDLIGCSAYEYIHALDSDAVSKSIHTLLSKGQAV 300
TGQYRFLARSGGYLWTQTQATVVSGGRGPQSESIVCVHFLISQVEETGVV 350
LSLEQTEQHSRRPIQRGAPSQKDTPNPGDSLDTPGPRILAFLHPPSLSEA 400
ALAADPRRFCSPDLRRLLGPILDGASVAATPSTPLATRHPQSPLSADLPD 450
ELPVGTENVHRLFTSGKDTEAVETDLDIAQDADALDLEMLAPYISMDDDF 500
QLNASEQLPRAYHRPLGAVPRPRARSFHGLSPPALEPSLLPRWGSDPRLS 550
CSSPSRGDPSASSPMAGARKRTLAQSSEDEDEGVELLGVRPPKRSPSPEH 600
ENFLLFPLSLSFLLTGGPAPGSLQDPSTPLLNLNEPLGLGPSLLSPYSDE 650
DTTQPGGPFQPRAGSAQAD                                669
```

Predicted monopartite NLS

Pos.	Sequence	Score

Predicted bipartite NLS

Pos.	Sequence	Score
20	RDAARSRRSQETEVLYQLAHTLPFARGVSAHLDK	2.3
153	RRKVEAPTERCFSLRMKSTLTSRGRTLNLK	2.6
193	GHMRAYKPPAQTSPAGSPDSEPPLQCLVLI	2
196	RAYKPPAQTSPAGSPDSEPPLQCLVLICE	2.7
196	RAYKPPAQTSPAGSPDSEPPLQCLVLICEAI	3.3
361	RRPIQRGAPSQKDTPNPGDSLDTPGPRILAFL	2.3
405	DPRRFCSPDLRRLLGPILDGASVAATPSTP	2
407	RRFCSPDLRRLLGPILDGASVAATPSTPLATRHPQS	3.3

438	RHPQSPLSADLPDELPVGTENVHRLFTS	3.6
521	RPRARSFHGLSPPALEPSLLPRWGSDPRLSCS	2
542	RWGSDPRLSCSSPSRGDPSASSPMAGARKRTLA	3.7
569	RKRTLAQSSEDEDEGVELLGVRPPKRSP	2.3
567	GARKRTLAQSSEDEDEGVELLGVRPP	2.5
567	GARKRTLAQSSEDEDEGVELLGVRPPKRS	3.5
567	GARKRTLAQSSEDEDEGVELLGVRPPKRSP	5.2
567	GARKRTLAQSSEDEDEGVELLGVRPPKRSPS	6.8
569	RKRTLAQSSEDEDEGVELLGVRPPKRSPSP	5.9
590	RPPKRSPSPEHENFLLFPLSLSFLLTGGP	2
590	RPPKRSPSPEHENFLLFPLSLSFLLTGGPAP	3

NLStradamus

Pre-loaded models cutoff 0.1 4 state HMM static

7 - RARSTTELRKEKSRDAARSRRSQE - 30

512 - YHRPLGAVPRPRARSFHGLSPP - 533

563 - SPMAGARKRTLAQSSEDEDEGVELLGVRPPKRSPS - 597

NucPred

The NucPred score for your sequence is 0.90 (see score help below)

```
  1  MALGLQRARSTTELRKEKSRDAARSRRSQETEVLYQLAHTLPFARGVSAH   50
 51  LDKASIMRLTISYLRMHRLCAAGEWNQVGAGGEPLDACYLKALEGFVMVL  100
101  TAEGDMAYLSENVSKHLGLSQLELIGHSIFDFIHPCDQEELQDALTPQQT  150
151  LSRRKVEAPTERCFSLRMKSTLTSRGRTLNLKAATWKVLNCSGHMRAYKP  200
201  PAQTSPAGSPDSEPPLQCLVLICEAIPHPGSLEPPLGRGAFLSRHSLDMK  250
251  FTYCDDRIAEVAGYSPDDLIGCSAYEYIHALDSDAVSKSIHTLLSKGQAV  300
301  TGQYRFLARSGGYLWTQTQATVVSGGRGPQSESIVCVHFLISQVEETGVV  350
351  LSLEQTEQHSRRPIQRGAPSQKDTPNPGDSLDTPGPRILAFLHPPSLSEA  400
401  ALAADPRRFCSPDLRRLLGPILDGASVAATPSTPLATRHPQSPLSADLPD  450
451  ELPVGTENVHRLFTSGKDTEAVETDLDIAQDADALDLEMLAPYISMDDDF  500
501  QLNASEQLPRAYHRPLGAVPRPRARSFHGLSPPALEPSLLPRWGSDPRLS  550
551  CSSPSRGDPSASSPMAGARKRTLAQSSEDEDEGVELLGVRPPKRSPSPEH  600
601  ENFLLFPLSLSFLLTGGPAPGSLQDPSTPLLNLNEPLGLGPSLLSPYSDE  650
651  DTTQPGGPFQPRAGSAQAD                                 669
```

Positively and negatively influencing subsequences are coloured according to the following scale:

(non-nuclear) negative positive (nuclear)

PSORTII

NUCDISC: discrimination of nuclear localization signals

```
pat4: none
pat7: none
bipartite: none
content of basic residues:   9.6%
NLS Score: -0.47
```

SeqNLS

Prediction result (The predicted NLS(s) are underlined)

Hif3a: MALGLQRARSTTELRKEKSRDAARSRRSQETEVLYQLAHTLPFARGVSAHLDKASIMRLT

The predicted NLS(s) (score cutoff = 0.5)

Protein ID	Predicted NLS	Start	Stop	The highest score of matches within the prediction
Hif3a	RSRRSQETEVLY	24	35	0.805

ELM

Motif Probability Cutoff: 100, 50   No positive NLS/NES result

NES Finder

Position	NES	Type
34	LYQLAHTLPF	2-3-1
57	MRLTISYLRM	3-2-1
487	LEMLAPYISM	2-3-1
603	FLLFPLSLSF	2-3-1
630	LLNLNEPLGL	2-3-1

LocNES

Protein Name	Position	Sequence	Score
>LocNES1659617645_0	33-47	QETEVLYQLAHTLPF	0.091
>LocNES1659617645_0	49-63	RGVSAHLDKASIMRL	0.069
>LocNES1659617645_0	51-65	VSAHLDKASIMRLTI	0.095
>LocNES1659617645_0	54-68	HLDKASIMRLTISYL	0.224
>LocNES1659617645_0	56-70	DKASIMRLTISYLRM	0.265
>LocNES1659617645_0	88-102	PLDACYLKALEGFVM	0.043
>LocNES1659617645_0	89-103	LDACYLKALEGFVMV	0.011

>LocNES1659617645_0	90-104	DACYLKALEGFVMVL	0.016
>LocNES1659617645_0	99-113	GFVMVLTAEGDMAYL	0.075
>LocNES1659617645_0	109-123	DMAYLSENVSKHLGL	0.237
>LocNES1659617645_0	114-128	SENVSKHLGLSQLEL	0.256
>LocNES1659617645_0	122-136	GLSQLELIGHSIFDF	0.019
>LocNES1659617645_0	156-170	SRRKVEAPTERCFSL	0.016
>LocNES1659617645_0	334-348	QSESIVCVHFLISQV	0.022
>LocNES1659617645_0	343-357	FLISQVEETGVVLSL	0.032
>LocNES1659617645_0	444-458	PQSPLSADLPDELPV	0.089
>LocNES1659617645_0	468-482	TSGKDTEAVETDLDI	0.014
>LocNES1659617645_0	477-491	ETDLDIAQDADALDL	0.387
>LocNES1659617645_0	486-500	ADALDLEMLAPYISM	0.108
>LocNES1659617645_0	600-614	PSPEHENFLLFPLSL	0.265
>LocNES1659617645_0	602-616	PEHENFLLFPLSLSF	0.539
>LocNES1659617645_0	603-617	EHENFLLFPLSLSFL	0.500
>LocNES1659617645_0	604-618	HENFLLFPLSLSFLL	0.320
>LocNES1659617645_0	629-643	DPSTPLLNLNEPLGL	0.547

NetNES 1.1 Server - prediction results

>Sequence - NetNES 1.1 prediction

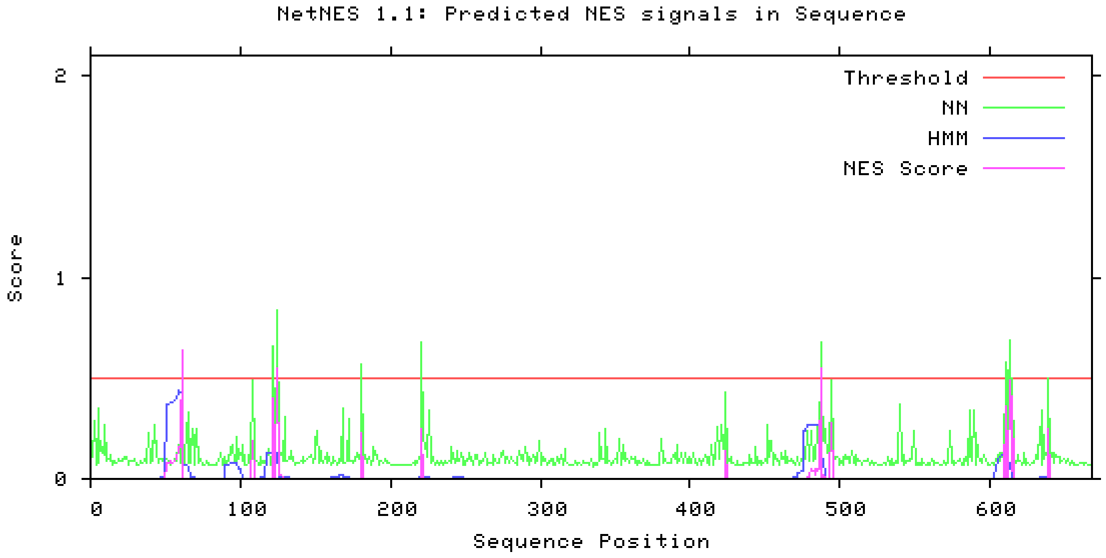

hSIM1

>sp|P81133|SIM1_HUMAN Single-minded homolog 1 OS=Homo sapiens OX=9606 GN=SIM1 PE=2 SV=2
MKEKSKNAARTRREKENSEFYELAKLLPLPSAITSQLDKASIIRLTTSYLKMRVVFPEGL
GEAWGHSSRTSPLDNVGRELGSHLLQTLDGFIFVVAPDGKIMYISETASVHLGLSQVELT

GNSIYEYIHPADHDEMTAVLTAHQPYHSHFVQEYEIERSFFLRMKCVLAKRNAGLTCGGY
KVIHCSGYLKIRQYSLDMSPFDGCYQNVGLVAVGHSLPPSAVTEIKLHSNMFMFRASLDM
KLIFLDSRVAELTGYEPQDLIEKTLYHHVHGCDTFHLRCAHHLLLVKGQVTTKYYRFLAK
HGGWVWVQSYATIVHNSRSSRPHCIVSVNYVLTDTEYKGLQLSLDQISASKPAFSYTSSS
TPTMTDNRKGAKSRLSSSKSKSRTSPYPQYSGFHTERSESDHDSQWGGSPLTDTASPQLL
DPADRPGSQHDASCAYRQFSDRSSLCYGFALDHSRLVEERHFHTQACEGGRCEAGRYFLG
TPQAGREPWWGSRAALPLTKASPESREAYENSMPHIASVHRIHGRGHWDEDSVVSSPDPG
SASESGDRYRTEQYQSSPHEPSKIETLIRATQQMIKEEENRLQLRKAPSDQLASINGAGK
KHSLCFANYQQPPPTGEVCHGSALANTSPCDHIQQREGKMLSPHENDYDNSPTALSRISS
PNSDRISKSSLILAKDYLHSDISPHQTAGDHPTVSPNCFGSHRQYFDKHAYTLTGYALEH
LYDSETIRNYSLGCNGSHFDVTSHLRMQPDPAQGHKGTSVIITNGS

cNLS Mapper Result cut-off 2.0

Predicted NLSs in query sequence

```
MKEKSKNAARTRREKENSEFYELAKLLPLPSAITSQLDKASIIRLTTSYL  50
KMRVVFPEGLGEAWGHSSRTSPLDNVGRELGSHLLQTLDGFIFVVAPDGK 100
IMYISETASVHLGLSQVELTGNSIYEYIHPADHDEMTAVLTAHQPYHSHF 150
VQEYEIERSFFLRMKCVLAKRNAGLTCGGYKVIHCSGYLKIRQYSLDMSP 200
FDGCYQNVGLVAVGHSLPPSAVTEIKLHSNMFMFRASLDMKLIFLDSRVA 250
ELTGYEPQDLIEKTLYHHVHGCDTFHLRCAHHLLLVKGQVTTKYYRFLAK 300
HGGWVWVQSYATIVHNSRSSRPHCIVSVNYVLTDTEYKGLQLSLDQISAS 350
KPAFSYTSSSTPTMTDNRKGAKSRLSSSKSKSRTSPYPQYSGFHTERSES 400
DHDSQWGGSPLTDTASPQLLDPADRPGSQHDASCAYRQFSDRSSLCYGFA 450
LDHSRLVEERHFHTQACEGGRCEAGRYFLGTPQAGREPWWGSRAALPLTK 500
ASPESREAYENSMPHIASVHRIHGRGHWDEDSVVSSPDPGSASESGDRYR 550
TEQYQSSPHEPSKIETLIRATQQMIKEEENRLQLRKAPSDQLASINGAGK 600
KHSLCFANYQQPPPTGEVCHGSALANTSPCDHIQQREGKMLSPHENDYDN 650
SPTALSRISSPNSDRISKSSLILAKDYLHSDISPHQTAGDHPTVSPNCFG 700
SHRQYFDKHAYTLTGYALEHLYDSETIRNYSLGCNGSHFDVTSHLRMQPD 750
PAQGHKGTSVIITNGS                                   766
```

Predicted monopartite NLS

Pos.	Sequence	Score

Predicted bipartite NLS

Pos.	Sequence	Score
2	KEKSKNAARTRREKENSEFYELAKLLPLP	2
4	KSKNAARTRREKENSEFYELAKLLPLPSAI	3
6	KNAARTRREKENSEFYELAKLLPLPSAITS	2.3
10	RTRREKENSEFYELAKLLPLPSAITSQLDKASI	2
74	DNVGRELGSHLLQTLDGFIFVVAPDGKIMYIS	2.3
158	RSFFLRMKCVLAKRNAGLTCGGYKVIHCSGYLKI	2.4
163	RMKCVLAKRNAGLTCGGYKVIHCSGYLKIRQYS	3.3
163	RMKCVLAKRNAGLTCGGYKVIHCSGYLKIRQY	2.4
163	RMKCVLAKRNAGLTCGGYKVIHCSGYLKIRQYSLD	3.4
187	GYLKIRQYSLDMSPFDGCYQNVGLVAVGH	2.3

223	TEIKLHSNMFMFRASLDMKLIFLDSRVAELT	2.2
235	RASLDMKLIFLDSRVAELTGYEPQDLIEKTLYH	2.8
293	KYYRFLAKHGGWVWVQSYATIVHNSRSS	2.4
347	ISASKPAFSYTSSSTPTMTDNRKGAKSRLSSS	2.7
486	REPWWGSRAALPLTKASPESREAYENSMPHIASV	2
493	RAALPLTKASPESREAYENSMPHIASVHRIHGRG	2.2
581	RLQLRKAPSDQLASINGAGKKHSLCFANYQQPP	3
597	GAGKKHSLCFANYQQPPPTGEVCHGSALANT	2
600	KKHSLCFANYQQPPPTGEVCHGSALANTS	3.6
636	REGKMLSPHENDYDNSPTALSRISSPNSDRI	2.2
703	RQYFDKHAYTLTGYALEHLYDSETIRNYSLGC	2.6

NLStradamus

cutoff 0.1; Pre-loaded models 4 state HMM static

2 - KEKSKNAARTRREKENSEFYELA - 24
365 - TDNRKGAKSRLSSSKSKSRTSP - 386

PSORTII

NUCDISC: discrimination of nuclear localization signals
pat4: none
pat7: none
bipartite: none
content of basic residues: 10.3%
NLS Score: -0.47

SeqNLS

Prediction result (The predicted NLS(s) are underlined)

Sim1: MKEKSKNAARTRREKENSEFYELAKLLPLPSAITSQLDKASIIRLTTSYLKMRVVFPEGL

The predicted NLS(s) (score cutoff = 0.5)

Protein ID	Predicted NLS	Start	Stop	The highest score of matches within the prediction

No NLS positive result

ELM

Motif Probability Cutoff: 100 and 50 No positive NLS/NES result

NES Finder

Position NES Type

==============================

20 FYELAKLLPL 2-3-1

231 MFMFRASLDM 2-3-1

LocNES

Protein Name	Position	Sequence	Score
>LocNES1522303438_0	19-33	KENSEFYELAKLLPL	0.254
>LocNES1522303438_0	35-49	SAITSQLDKASIIRL	0.253
>LocNES1522303438_0	42-56	DKASIIRLTTSYLKM	0.368
>LocNES1522303438_0	46-60	IIRLTTSYLKMRVVF	0.011
>LocNES1522303438_0	83-97	ELGSHLLQTLDGFIF	0.062
>LocNES1522303438_0	84-98	LGSHLLQTLDGFIFV	0.066
>LocNES1522303438_0	85-99	GSHLLQTLDGFIFVV	0.042
>LocNES1522303438_0	102-116	DGKIMYISETASVHL	0.130
>LocNES1522303438_0	104-118	KIMYISETASVHLGL	0.200
>LocNES1522303438_0	107-121	YISETASVHLGLSQV	0.030
>LocNES1522303438_0	109-123	SETASVHLGLSQVEL	0.088
>LocNES1522303438_0	217-231	VGHSLPPSAVTEIKL	0.042
>LocNES1522303438_0	223-237	PSAVTEIKLHSNMFM	0.087
>LocNES1522303438_0	230-244	KLHSNMFMFRASLDM	0.042
>LocNES1522303438_0	232-246	HSNMFMFRASLDMKL	0.029
>LocNES1522303438_0	233-247	SNMFMFRASLDMKLI	0.011
>LocNES1522303438_0	234-248	NMFMFRASLDMKLIF	0.034
>LocNES1522303438_0	235-249	MFMFRASLDMKLIFL	0.113
>LocNES1522303438_0	242-256	LDMKLIFLDSRVAEL	0.046
>LocNES1522303438_0	275-289	GCDTFHLRCAHHLLL	0.098
>LocNES1522303438_0	276-290	CDTFHLRCAHHLLLV	0.023
>LocNES1522303438_0	737-751	GCNGSHFDVTSHLRM	0.029

NetNES 1.1 Server - prediction results

>Sequence - NetNES 1.1 prediction

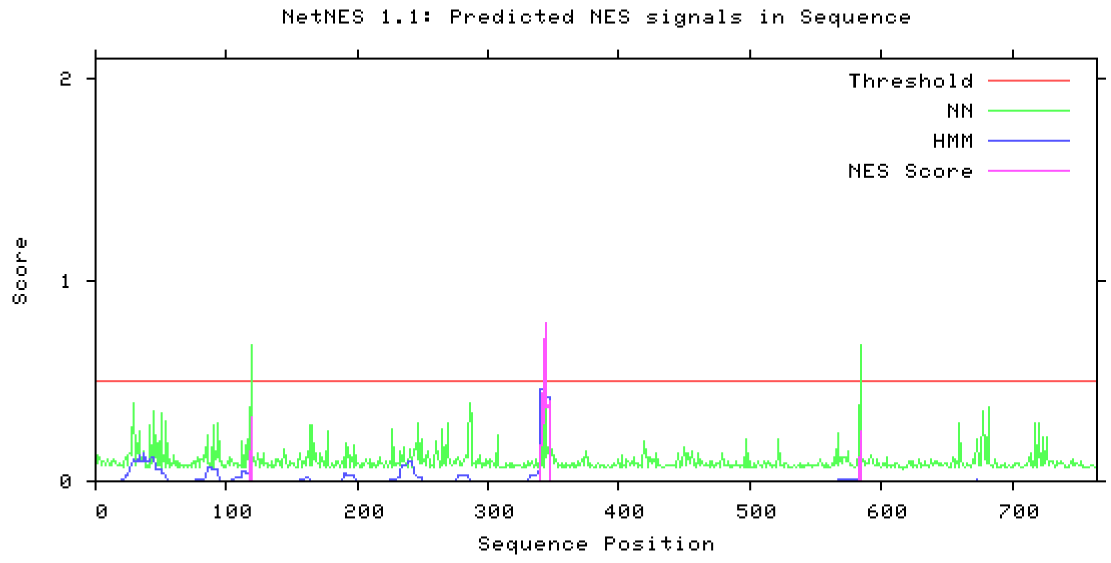

hSIM2

>sp|Q14190|SIM2_HUMAN Single-minded homolog 2 OS=Homo sapiens OX=9606 GN=SIM2 PE=1 SV=2
MKEKSKNAAKTRREKENGEFYELAKLLPLPSAITSQLDKASIIRLTTSYLKMRAVFPEGL
GDAWGQPSRAGPLDGVAKELGSHLLQTLDGFVFVVASDGKIMYISETASVHLGLSQVELT
GNSIYEYIHPSDHDEMTAVLTAHQPLHHHLLQEYEIERSFFLRMKCVLAKRNAGLTCSGY
KVIHCSGYLKIRQYMLDMSLYDSCYQIVGLVAVGQSLPPSAITEIKLYSNMFMFRASLDL
KLIFLDSRVTEVTGYEPQDLIEKTLYHHVHGCDVFHLRYAHHLLLVKGQVTTKYYRLLSK
RGGWVWVQSYATVVHNSRSSRPHCIVSVNYVLTEIEYKELQLSLEQVSTAKSQDSWRTAL
STSQETRKLVKPKNTKMKTKLRTNPYPPQQYSSFQMDKLECGQLGNWRASPPASAAAPPE
LQPHSESSDLLYTPSYSLPFSYHYGHFPLDSHVFSSKKPMLPAKFGQPQGSPCEVARFFL
STLPASGECQWHYANPLVPSSSSPAKNPPEPPANTARHSLVPSYEAPAAAVRRFGEDTAP
PSFPSCGHYREEPALGPAKAARQAARDGARLALARAAPECCAPPTPEAPGAPAQLPFVLL
NYHRVLARRGPLGGAAPAASGLACAPGGPEAATGALRLRHPSPAATSPPGAPLPHYLGAS
VIITNGR

cNLS Mapper Result cut-off score 2.0

Predicted NLSs in query sequence

MKEKSKNAAKTRREKENGEFYELAKLLPLPSAITSQLDKASIIRLTTSYL 50
KMRAVFPEGLGDAWGQPSRAGPLDGVAKELGSHLLQTLDGFVFVVASDGK 100
IMYISETASVHLGLSQVELTGNSIYEYIHPSDHDEMTAVLTAHQPLHHHL 150
LQEYEIERSFFLRMKCVLAKRNAGLTCSGYKVIHCSGYLKIRQYMLDMSL 200
YDSCYQIVGLVAVGQSLPPSAITEIKLYSNMFMFRASLDLKLIFLDSRVT 250
EVTGYEPQDLIEKTLYHHVHGCDVFHLRYAHHLLLVKGQVTTKYYRLLSK 300
RGGWVWVQSYATVVHNSRSSRPHCIVSVNYVLTEIEYKELQLSLEQVSTA 350
KSQDSWRTALSTSQETRKLVKPKNTKMKTKLRTNPYPPQQYSSFQMDKLE 400
CGQLGNWRASPPASAAAPPELQPHSESSDLLYTPSYSLPFSYHYGHFPLD 450
SHVFSSKKPMLPAKFGQPQGSPCEVARFFLSTLPASGECQWHYANPLVPS 500
SSSPAKNPPEPPANTARHSLVPSYEAPAAAVRRFGEDTAPPSFPSCGHYR 550

```
EEPALGPAKAARQAARDGARLALARAAPECCAPPTPEAPGAPAQLPFVLL  600
NYHRVLARRGPLGGAAPAASGLACAPGGPEAATGALRLRHPSPAATSPPG  650
APLPHYLGASVIITNGR                                   667
```

Predicted monopartite NLS		
**Pos.**	**Sequence**	**Score**

Predicted bipartite NLS		
**Pos.**	**Sequence**	**Score**
2	KEKSKNAAKTRREKENGEFYELAKLLPLP	2
4	KSKNAAKTRREKENGEFYELAKLLPLPSAI	2.9
6	KNAAKTRREKENGEFYELAKLLPLPSAITS	3.7
74	DGVAKELGSHLLQTLDGFVFVVASDGKIMYIS	3.5
158	RSFFLRMKCVLAKRNAGLTCSGYKVIHCSGYLKI	2.4
163	RMKCVLAKRNAGLTCSGYKVIHCSGYLKIRQY	3.5
163	RMKCVLAKRNAGLTCSGYKVIHCSGYLKIRQYM	2.7
163	RMKCVLAKRNAGLTCSGYKVIHCSGYLKIRQYMLD	3.2
187	GYLKIRQYMLDMSLYDSCYQIVGLVAVGQ	2.1
235	RASLDLKLIFLDSRVTEVTGYEPQDLIEKTLYH	2.8
273	DVFHLRYAHHLLLVKGQVTTKYYRLLSKRGGW	2.2
293	KYYRLLSKRGGWVWVQSYATVVHNSRSS	2.2
296	RLLSKRGGWVWVQSYATVVHNSRSSRPHCI	2.3
296	RLLSKRGGWVWVQSYATVVHNSRSSRPHCIVS	2.7
351	KSQDSWRTALSTSQETRKLVKPKNTKMK	6.1
365	ETRKLVKPKNTKMKTKLRTNPYPPQQYSSF	2.4
373	KNTKMKTKLRTNPYPPQQYSSFQMDKLECG	2.8
375	TKMKTKLRTNPYPPQQYSSFQMDKLECGQL	2.6
454	FSSKKPMLPAKFGQPQGSPCEVARFFLSTLP	2.6
454	FSSKKPMLPAKFGQPQGSPCEVARFFLSTLP	2.4
556	GPAKAARQAARDGARLALARAAPECCAPPTP	2.4
637	RLRHPSPAATSPPGAPLPHYLGASVIITN	2.9

NLStradamus

Prediction cutoff 0.1; Pre-loaded models 4 state HMM static

2 - KEKSKNAAKTRREKENGEFYELAK - 25

363 - SQETRKLVKPKNTKMKTKLRTNPY - 386

556 - GPAKAARQAARDGARLA - 572

NucPred

The NucPred score for your sequence is 0.47 (see score help below)

```
 1  MKEKSKNAAKTRREKENGEFYELAKLLPLPSAITSQLDKASIIRLTTSYL   50
51  KMRAVFPEGLGDAWGQPSRAGPLDGVAKELGSHLLQTLDGFVFVVASDGK  100
```

```
101   IMYISETASVHLGLSQVELTGNSIYEYIHPSDHDEMTAVLTAHQPLHHHL   150
151   LQEYEIERSFFLRMKCVLAKRNAGLTCSGYKVIHCSGYLKIRQYMLDMSL   200
201   YDSCYQIVGLVAVGQSLPPSAITEIKLYSNMFMFRASLDLKLIFLDSRVT   250
251   EVTGYEPQDLIEKTLYHHVHGCDVFHLRYAHHLLLVKGQVTTKYYRLLSK   300
301   RGGWVWVQSYATVVHNSRSSRPHCIVSVNYVLTEIEYKELQLSLEQVSTA   350
351   KSQDSWRTALSTSQETRKLVKPKNTKMKTKLRTNPYPPQQYSSFQMDKLE   400
401   CGQLGNWRASPPASAAAPPELQPHSESSDLLYTPSYSLPFSYHYGHFPLD   450
451   SHVFSSKKPMLPAKFGQPQGSPCEVARFFLSTLPASGECQWHYANPLVPS   500
501   SSSPAKNPPEPPANTARHSLVPSYEAPAAAVRRFGEDTAPPSFPSCGHYR   550
551   EEPALGPAKAARQAARDGARLALARAAPECCAPPTPEAPGAPAQLPFVLL   600
601   NYHRVLARRGPLGGAAPAASGLACAPGGPEAATGALRLRHPSPAATSPPG   650
651   APLPHYLGASVIITNGR                                    667
```

Positively and negatively influencing subsequences are coloured according to the following scale:

(non-nuclear) negative 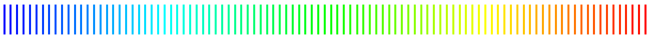 positive

PSORTII

NUCDISC: discrimination of nuclear localization signals

```
      pat4: none
      pat7: none
      bipartite: none
      content of basic residues:  10.3%
      NLS Score: -0.47
```

SeqNLS

Prediction result (The predicted NLS(s) are underlined)

Sim2: MKEK<u>SKNAAKTRREKENGEF</u>YELAKLLPLPSAITSQLDKASIIRLTTSYLKMRAVFPEGL

The predicted NLS(s) (score cutoff = 0.5)

Protein ID	Predicted NLS	Start	Stop	The highest score of matches within the prediction
Sim2	SKNAAKTRREKENGEF	5	20	0.598

ELM

Motif Probability Cutoff: 100 and 50 No positive NLS/NES result

NES Finder

Position   NES   Type

==============================

8   MANSINGLEM   3-2-1

65   FYELAKLLPL   2-3-1

276   MFMFRASLDL   2-3-1

LocNES

Protein Name	Position	Sequence	Score
>LocNES112173684_0	19-33	KENGEFYELAKLLPL	0.228
>LocNES112173684_0	35-49	SAITSQLDKASIIRL	0.352
>LocNES112173684_0	42-56	DKASIIRLTTSYLKM	0.338
>LocNES112173684_0	83-97	ELGSHLLQTLDGFVF	0.029
>LocNES112173684_0	84-98	LGSHLLQTLDGFVFV	0.055
>LocNES112173684_0	85-99	GSHLLQTLDGFVFVV	0.032
>LocNES112173684_0	102-116	DGKIMYISETASVHL	0.130
>LocNES112173684_0	104-118	KIMYISETASVHLGL	0.200
>LocNES112173684_0	107-121	YISETASVHLGLSQV	0.030
>LocNES112173684_0	109-123	SETASVHLGLSQVEL	0.088
>LocNES112173684_0	190-204	SGYLKIRQYMLDMSL	0.105
>LocNES112173684_0	217-231	VGQSLPPSAITEIKL	0.048
>LocNES112173684_0	223-237	PSAITEIKLYSNMFM	0.044
>LocNES112173684_0	230-244	KLYSNMFMFRASLDL	0.081
>LocNES112173684_0	232-246	YSNMFMFRASLDLKL	0.124
>LocNES112173684_0	233-247	SNMFMFRASLDLKLI	0.039
>LocNES112173684_0	234-248	NMFMFRASLDLKLIF	0.136
>LocNES112173684_0	235-249	MFMFRASLDLKLIFL	0.080
>LocNES112173684_0	242-256	LDLKLIFLDSRVTEV	0.018
>LocNES112173684_0	275-289	GCDVFHLRYAHHLLL	0.083
>LocNES112173684_0	276-290	CDVFHLRYAHHLLLV	0.026
>LocNES112173684_0	325-339	RPHCIVSVNYVLTEI	0.026
>LocNES112173684_0	590-604	PEAPGAPAQLPFVLL	0.054
>LocNES112173684_0	653-667	PGAPLPHYLGASVII	0.085

NetNES 1.1 Server - prediction results

```
>Sequence - NetNES 1.1 prediction
```

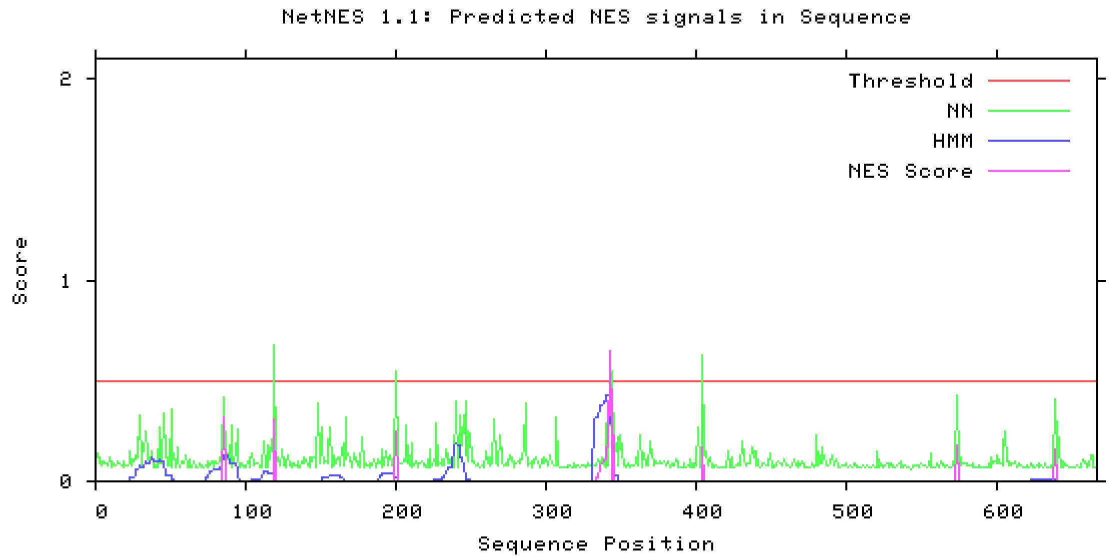

hClock

```
>sp|O15516|CLOCK_HUMAN Circadian locomoter output cycles protein kaput
OS=Homo sapiens OX=9606 GN=CLOCK PE=1 SV=1
MLFTVSCSKMSSIVDRDDSSIFDGLVEEDDKDKAKRVSRNKSEKKRRDQFNVLIKELGSM
LPGNARKMDKSTVLQKSIDFLRKHKEITAQSDASEIRQDWKPTFLSNEEFTQLMLEALDG
FFLAIMTDGSIIYVSESVTSLLEHLPSDLVDQSIFNFIPEGEHSEVYKILSTHLLESDSL
TPEYLKSKNQLEFCCHMLRGTIDPKEPSTYEYVKFIGNFKSLNSVSSSAHNGFEGTIQRT
HRPSYEDRVCFVATVRLATPQFIKEMCTVEEPNEEFTSRHSLEWKFLFLDHRAPPIIGYL
PFEVLGTSGYDYYHVDDLENLAKCHEHLMQYGKGKSCYYRFLTKGQQWIWLQTHYYITYH
QWNSRPEFIVCTHTVVSYAEVRAERRRELGIEESLPETAADKSQDSGSDNRINTVSLKEA
LERFDHSPTPSASSRSSRKSSHTAVSDPSSTPTKIPTDTSTPPRQHLPAHEKMVQRRSSF
SSQSINSQSVGSSLTQPVMSQATNLPIPQGMSQFQFSAQLGAMQHLKDQLEQRTRMIEAN
IHRQQEELRKIQEQLQMVHGQGLQMFLQQSNPGLNFGSVQLSSGNSSNIQQLAPINMQGQ
VVPTNQIQSGMNTGHIGTTQHMIQQQTLQSTSTQSQQNVLSGHSQQTSLPSQTQSTLTAP
LYNTMVISQPAAGSMVQIPSSMPQNSTQSAAVTTFTQDRQIRFSQGQQLVTKLVTAPVAC
GAVMVPSTMLMGQVVTAYPTFATQQQQSQTLSVTQQQQQQSSQEQQLTSVQQPSQAQLTQ
PPQQFLQTSRLLHGNPSTQLILSAAFPLQQSTFPQSHHQQHQSQQQQQLSRHRTDSLPDP
SKVQPQ
```

cNLS Mapper Result score 2.0

Predicted NLSs in query sequence

```
MLFTVSCSKMSSIVDRDDSSIFDGLVEEDDKDKAKRVSRNKSEKKRRDQF  50
NVLIKELGSMLPGNARKMDKSTVLQKSIDFLRKHKEITAQSDASEIRQDW 100
KPTFLSNEEFTQLMLEALDGFFLAIMTDGSIIYVSESVTSLLEHLPSDLV 150
DQSIFNFIPEGEHSEVYKILSTHLLESDSLTPEYLKSKNQLEFCCHMLRG 200
TIDPKEPSTYEYVKFIGNFKSLNSVSSSAHNGFEGTIQRTHRPSYEDRVC 250
FVATVRLATPQFIKEMCTVEEPNEEFTSRHSLEWKFLFLDHRAPPIIGYL 300
PFEVLGTSGYDYYHVDDLENLAKCHEHLMQYGKGKSCYYRFLTKGQQWIW 350
LQTHYYITYHQWNSRPEFIVCTHTVVSYAEVRAERRRELGIEESLPETAA 400
DKSQDSGSDNRINTVSLKEALERFDHSPTPSASSRSSRKSSHTAVSDPSS 450
TPTKIPTDTSTPPRQHLPAHEKMVQRRSSFSSQSINSQSVGSSLTQPVMS 500
QATNLPIPQGMSQFQFSAQLGAMQHLKDQLEQRTRMIEANIHRQQEELRK 550
```

```
IQEQLQMVHGQGLQMFLQQSNPGLNFGSVQLSSGNSSNIQQLAPINMQGQ  600
VVPTNQIQSGMNTGHIGTTQHMIQQQTLQSTSTQSQQNVLSGHSQQTSLP  650
SQTQSTLTAPLYNTMVISQPAAGSMVQIPSSMPQNSTQSAAVTTFTQDRQ  700
IRFSQGQQLVTKLVTAPVACGAVMVPSTMLMGQVVTAYPTFATQQQQSQT  750
LSVTQQQQQQSSQEQQLTSVQQPSQAQLTQPPQQFLQTSRLLHGNPSTQL  800
ILSAAFPLQQSTFPQSHHQQHQSQQQQQLSRHRTDSLPDPSKVQPQ      846
```

Predicted monopartite NLS		
Pos.	Sequence	Score

Predicted bipartite NLS		
Pos.	Sequence	Score
36	RVSRNKSEKKRRDQFNVLIKELGSMLPGNARKMDKS	3.7
39	RNKSEKKRRDQFNVLIKELGSMLPGNARKMDKS	2.8
39	RNKSEKKRRDQFNVLIKELGSMLPGNARKMDKST	7.3
39	RNKSEKKRRDQFNVLIKELGSMLPGNARKMDKST	3.2
239	RTHRPSYEDRVCFVATVRLATPQFIKEMCT	2.9
248	RVCFVATVRLATPQFIKEMCTVEEPNEEFTSRHSLE	2.1
256	RLATPQFIKEMCTVEEPNEEFTSRHSLEWKFLFLD	3.4
256	RLATPQFIKEMCTVEEPNEEFTSRHSLEWKFLFLD	2.4
411	RINTVSLKEALERFDHSPTPSASSRSSRKSSHT	3.5
411	RINTVSLKEALERFDHSPTPSASSRSSRKSSHTA	2.5
435	RSSRKSSHTAVSDPSSTPTKIPTDTSTPPR	2.4
451	TPTKIPTDTSTPPRQHLPAHEKMVQRRS	3.7
451	TPTKIPTDTSTPPRQHLPAHEKMVQRRSSFS	3.9
526	LKDQLEQRTRMIEANIHRQQEELRKIQE	3.1

NLStradamus cutoff 0.1

Posterior @ 0.1	27 - EEDDKDKAKRVSRNKSEKKRRDQFNVLIKELGSMLPGNARKMDKSTVLQKSIDFLRKHK - 85

NucPred

The NucPred score for your sequence is 0.94 (see score help below)

```
  1  MLFTVSCSKMSSIVDRDDSSIFDGLVEEDDKDKAKRVSRNKSEKKRRDQF   50
 51  NVLIKELGSMLPGNARKMDKSTVLQKSIDFLRKHKEITAQSDASEIRQDW  100
101  KPTFLSNEEFTQLMLEALDGFFLAIMTDGSIIYVSESVTSLLEHLPSDLV  150
151  DQSIFNFIPEGEHSEVYKILSTHLLESDSLTPEYLKSKNQLEFCCHMLRG  200
201  TIDPKEPSTYEYVKFIGNFKSLNSVSSSAHNGFEGTIQRTHRPSYEDRVC  250
251  FVATVRLATPQFIKEMCTVEEPNEEFTSRHSLEWKFLFLDHRAPPIIGYL  300
301  PFEVLGTSGYDYYHVDDLENLAKCHEHLMQYGKGKSCYYRFLTKGQQWIW  350
351  LQTHYYITYHQWNSRPEFIVCTHTVVSYAEVRAERRRELGIEESLPETAA  400
```

```
401   DKSQDSGSDNRINTVSLKEALERFDHSPTPSASSRSSRKSSHTAVSDPSS   450
451   TPTKIPTDTSTPPRQHLPAHEKMVQRRSSFSSQSINSQSVGSSLTQPVMS   500
501   QATNLPIPQGMSQFQFSAQLGAMQHLKDQLEQRTRMIEANIHRQQEELRK   550
551   IQEQLQMVHGQGLQMFLQQSNPGLNFGSVQLSSGNSSNIQQLAPINMQGQ   600
601   VVPTNQIQSGMNTGHIGTTQHMIQQQTLQSTSTQSQQNVLSGHSQQTSLP   650
651   SQTQSTLTAPLYNTMVISQPAAGSMVQIPSSMPQNSTQSAAVTTFTQDRQ   700
701   IRFSQGQQLVTKLVTAPVACGAVMVPSTMLMGQVVTAYPTFATQQQQSQT   750
751   LSVTQQQQQQSSQEQQLTSVQQPSQAQLTQPPQQFLQTSRLLHGNPSTQL   800
801   ILSAAFPLQQSTFPQSHHQQHQSQQQQQLSRHRTDSLPDPSKVQPQ       846
```

Positively and negatively influencing subsequences are coloured according to the following scale:

(non-nuclear) negative

PSORTII

NUCDISC: discrimination of nuclear localization signals

```
      pat4: KKRR (5) at    44
      pat4: RKHK (3) at    82
      pat7: none
      bipartite: none
      content of basic residues:    8.5%
      NLS Score:  0.03
```

SeqNLS

Prediction result (The predicted NLS(s) are underlined)

Clock: MLFTV<u>SCSKMSSIVDR</u>DDSSIFDGLVEEDDKDAKRVSRNKSE<u>KKRR</u>DQFNVLIKELGSM

Definition of different colors in predictions

score range	color
0.1-0.3	
0.3-0.5	
0.5-0.7	
0.7-0.8	
0.8-0.86	
0.86-0.89	
>0.89	

The predicted NLS(s) (score cutoff = 0.5)

Protein ID	Predicted NLS	Start	Stop	The highest score of matches within the prediction
Clock	KKRR	44	47	0.862
Clock	SCSKMSSIVDR	6	16	0.522

ELM cutoff 100

No positive NLS result

TRG_NES_CRM1_1 7.626e-04

546-561

547-561

NES Finder

Position   NES   Type

==============================

Position	NES	Type
507	IPQGMSQFQF	3-2-1
548	LRKIQEQLQM	2-3-1
588	NIQQLAPINM	2-2-1

LocNES

Protein Name	Position	Sequence	Score
>LocNES1255047839_0	50-64	RRDQFNVLIKELGSM	0.139
>LocNES1255047839_0	111-125	NEEFTQLMLEALDGF	0.373
>LocNES1255047839_0	113-127	EFTQLMLEALDGFFL	0.290
>LocNES1255047839_0	115-129	TQLMLEALDGFFLAI	0.612
>LocNES1255047839_0	131-145	TDGSIIYVSESVTSL	0.015
>LocNES1255047839_0	247-261	PSYEDRVCFVATVRL	0.059
>LocNES1255047839_0	259-273	VRLATPQFIKEMCTV	0.038
>LocNES1255047839_0	292-306	FLDHRAPPIIGYLPF	0.030
>LocNES1255047839_0	414-428	NRINTVSLKEALERF	0.119
>LocNES1255047839_0	497-511	SLTQPVMSQATNLPI	0.062
>LocNES1255047839_0	506-520	ATNLPIPQGMSQFQF	0.056
>LocNES1255047839_0	547-561	RQQEELRKIQEQLQM	0.122
>LocNES1255047839_0	548-562	QQEELRKIQEQLQMV	0.103
>LocNES1255047839_0	571-585	LQQSNPGLNFGSVQL	0.050
>LocNES1255047839_0	587-601	SGNSSNIQQLAPINM	0.073
>LocNES1255047839_0	657-671	TQSTLTAPLYNTMVI	0.047
>LocNES1255047839_0	721-735	PVACGAVMVPSTMLM	0.029
>LocNES1255047839_0	798-812	GNPSTQLILSAAFPL	0.136

NetNES 1.1 Server - prediction results

```
>Sequence - NetNES 1.1 prediction
```

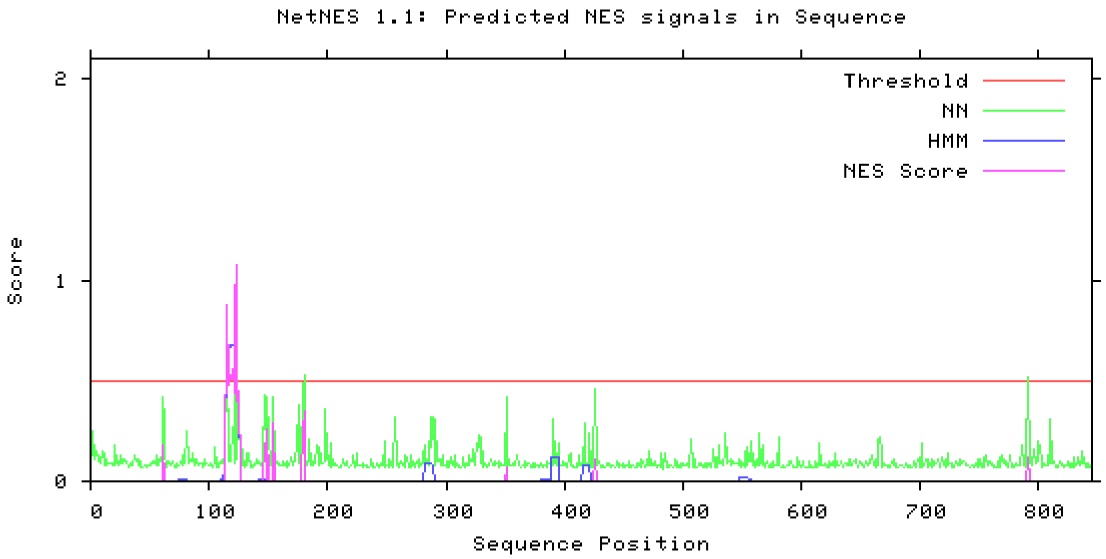

hNPAS1

>sp|Q99742|NPAS1_HUMAN Neuronal PAS domain-containing protein 1 OS=Homo sapiens OX=9606 GN=NPAS1 PE=2 SV=2
MAAPYPGSGGGSEVKCVGGRGASVPWDFLPGLMVKAPSGPCLQAQRKEKSRNAARSRRGK
ENLEFFELAKLLPLPGAISSQLDKASIVRLSVTYLRLRRFAALGAPPWGLRAAGPPAGLA
PGRRGPAALVSEVFEQHLGGHILQSLDGFVFALNQEGKFLYISETVSIYLGLSQVEMTGS
SVFDYIHPGDHSEVLEQLGLRTPTPGPPTPPSVSSSSSSSSSLADTPEIEASLTKVPPSS
LVQERSFFVRMKSTLTKRGLHVKASGYKVIHVTGRLRAHALGLVALGHTLPPAPLAELPL
HGHMIVFRLSLGLTILACESRVSDHMDLGPSELVGRSCYQFVHGQDATRIRQSHVDLLDK
GQVMTGYYRWLQRAGGFVWLQSVATVAGSGKSPGEHHVLWVSHVLSQAEGGQTPLDAFQL
PASVACEEASSPGPEPTEPEPPTEGKQAAPAENEAPQTQGKRIKVEPGPRETKGSEDSGD
EDPSSHPATPRPEFTSVIRAGVLKQDPVRPWGLAPPGDPPPTLLHAGFLPPVVRGLCTPG
TIRYGPAELGLVYPHLQRLGPGPALPEAFYPPLGLPYPGPAGTRLPRKGD

cNLS Mapper Result cut-off 2.0

Predicted NLSs in query sequence	
MAAPYPGSGGGSEVKCVGGRGASVPWDFLPGLMVKAPSGPCLQAQRKEKS	50
RNAARSRRGKENLEFFELAKLLPLPGAISSQLDKASIVRLSVTYLRLRRF	100
AALGAPPWGLRAAGPPAGLAPGRRGPAALVSEVFEQHLGGHILQSLDGFV	150
FALNQEGKFLYISETVSIYLGLSQVEMTGSSVFDYIHPGDHSEVLEQLGL	200
RTPTPGPPTPPSVSSSSSSSSSLADTPEIEASLTKVPPSSLVQERSFFVR	250
MKSTLTKRGLHVKASGYKVIHVTGRLRAHALGLVALGHTLPPAPLAELPL	300
HGHMIVFRLSLGLTILACESRVSDHMDLGPSELVGRSCYQFVHGQDATRI	350
RQSHVDLLDKGQVMTGYYRWLQRAGGFVWLQSVATVAGSGKSPGEHHVLW	400
VSHVLSQAEGGQTPLDAFQLPASVACEEASSPGPEPTEPEPPTEGKQAAP	450
AENEAPQTQGKRIKVEPGPRETKGSEDSGDEDPSSHPATPRPEFTSVIRA	500
GVLKQDPVRPWGLAPPGDPPPTLLHAGFLPPVVRGLCTPGTIRYGPAELG	550
LVYPHLQRLGPGPALPEAFYPPLGLPYPGPAGTRLPRKGD	590

Predicted monopartite NLS		
Pos.	Sequence	Score

Predicted bipartite NLS		
Pos.	Sequence	Score
33	MVKAPSGPCLQAQRKEKSRNAARSRRGKE	3.1
45	QRKEKSRNAARSRRGKENLEFFELAKLLP	2.5
46	RKEKSRNAARSRRGKENLEFFELAKLLPLP	2.4
46	RKEKSRNAARSRRGKENLEFFELAKLLPLP	2.4
51	RNAARSRRGKENLEFFELAKLLPLPGAISSQLDK	2.3
55	RSRRGKENLEFFELAKLLPLPGAISSQLDKASI	2.9
89	RLSVTYLRLRRFAALGAPPWGLRAAGPPAGLAPG	2.2
96	RLRRFAALGAPPWGLRAAGPPAGLAPGRRGP	2
230	EASLTKVPPSSLVQERSFFVRMKSTLTKRGLH	3.3
245	RSFFVRMKSTLTKRGLHVKASGYKVIHVTGRLRA	2.7
250	RMKSTLTKRGLHVKASGYKVIHVTGRLRAHALGL	3
458	TQGKRIKVEPGPRETKGSEDSGDEDPSSHP	2.2

NLStradamus

Posterior cutoff 0.1

33 - MVKAPSGPCLQAQRKEKSRNAARSRRGKENLEFFELAKLLPLPG - 76

96 - RLRRFAALGAPPWGLRAAGPPAGLAPGRRGPAA - 128

258 - RG - 259

456 - PQTQGKRIKVEPGPRETKGS - 475

579 - GPAGTRLPRKGD - 590

NucPred

The NucPred score for your sequence is 0.22 (see score help below)

```
  1  MAAPYPGSGGGSEVKCVGGRGASVPWDFLPGLMVKAPSGPCLQAQRKEKS   50
 51  RNAARSRRGKENLEFFELAKLLPLPGAISSQLDKASIVRLSVTYLRLRRF  100
101  AALGAPPWGLRAAGPPAGLAPGRRGPAALVSEVFEQHLGGHILQSLDGFV  150
151  FALNQEGKFLYISETVSIYLGLSQVEMTGSSVFDYIHPGDHSEVLEQLGL  200
201  RTPTPGPPTPPSVSSSSSSSSLADTPEIEASLTKVPPSSLVQERSFFVR   250
251  MKSTLTKRGLHVKASGYKVIHVTGRLRAHALGLVALGHTLPPAPLAELPL  300
301  HGHMIVFRLSLGLTILACESRVSDHMDLGPSELVGRSCYQFVHGQDATRI  350
351  RQSHVDLLDKGQVMTGYYRWLQRAGGFVWLQSVATVAGSGKSPGEHHVLW  400
401  VSHVLSQAEGGQTPLDAFQLPASVACEEASSPGPEPTEPEPPTEGKQAAP  450
451  AENEAPQTQGKRIKVEPGPRETKGSEDSGDEDPSSHPATPRPEFTSVIRA  500
501  GVLKQDPVRPWGLAPPGDPPPTLLHAGFLPPVVRGLCTPGTIRYGPAELG  550
551  LVYPHLQRLGPGPALPEAFYPPLGLPYPGPAGTRLPRKGD            590
```

Positively and negatively influencing subsequences are coloured according to the following scale:

(non-nuclear) negative ||||||||||||||||||||||||||||||||||||||||||||||||||||||||||||||||||||||||||||||||||||||||||||||||||||||| positive

PSORTII

NUCDISC: discrimination of nuclear localization signals

```
pat4: none
pat7: none
bipartite: none
content of basic residues:   9.7%
NLS Score: -0.47
```

SeqNLS

Prediction result (The predicted NLS(s) are underlined)

Npas1: MAAPYPGSGGGSEVKCVGGRGASVPWDFLPGLMVKAPSGP**CLQAQRKEK**SR**NAARSRRGK**

Definition of different colors in predictions

score range	color
0.1-0.3	
0.3-0.5	
0.5-0.7	
0.7-0.8	
0.8-0.86	
0.86-0.89	
>0.89	

The predicted NLS(s) (score cutoff = 0.3)

Protein ID	Predicted NLS	Start	Stop	The highest score of matches within the prediction
Npas1	CLQAQRKEK	41	49	0.447
Npas1	NAARSRRGK	52	60	0.351

ELM score 100

TRG_NLS_MonoExtC_3 7.252e-04

GKRIKVE 460-466

Monopartite variant of the classical basically charged NLS. C-extended version.

No positive NES result

NES Finder

Position	NES	Type
65	FFELAKLLPL	2-3-1
88	VRLSVTYLRL	3-2-1
300	LHGHMIVFRL	3-2-1
304	MIVFRLSLGL	2-3-1
306	VFRLSLGLTI	2-3-1

LocNES

Protein Name	Position	Sequence	Score
>LocNES859122758_0	24-38	RGASVPWDFLPGLMV	0.049
>LocNES859122758_0	64-78	KENLEFFELAKLLPL	0.245
>LocNES859122758_0	80-94	GAISSQLDKASIVRL	0.123
>LocNES859122758_0	82-96	ISSQLDKASIVRLSV	0.095
>LocNES859122758_0	85-99	QLDKASIVRLSVTYL	0.215
>LocNES859122758_0	87-101	DKASIVRLSVTYLRL	0.295
>LocNES859122758_0	90-104	SIVRLSVTYLRLRRF	0.143
>LocNES859122758_0	141-155	HLGGHILQSLDGFVF	0.026
>LocNES859122758_0	143-157	GGHILQSLDGFVFAL	0.035
>LocNES859122758_0	158-172	NQEGKFLYISETVSI	0.024
>LocNES859122758_0	160-174	EGKFLYISETVSIYL	0.012
>LocNES859122758_0	162-176	KFLYISETVSIYLGL	0.037
>LocNES859122758_0	165-179	YISETVSIYLGLSQV	0.005
>LocNES859122758_0	167-181	SETVSIYLGLSQVEM	0.024
>LocNES859122758_0	226-240	SLADTPEIEASLTKV	0.011
>LocNES859122758_0	273-287	VIHVTGRLRAHALGL	0.780
>LocNES859122758_0	276-290	VTGRLRAHALGLVAL	0.235
>LocNES859122758_0	290-304	LGHTLPPAPLAELPL	0.200
>LocNES859122758_0	296-310	PAPLAELPLHGHMIV	0.144
>LocNES859122758_0	299-313	LAELPLHGHMIVFRL	0.083
>LocNES859122758_0	301-315	ELPLHGHMIVFRLSL	0.190
>LocNES859122758_0	303-317	PLHGHMIVFRLSLGL	0.093
>LocNES859122758_0	305-319	HGHMIVFRLSLGLTI	0.139
>LocNES859122758_0	306-320	GHMIVFRLSLGLTIL	0.211
>LocNES859122758_0	410-424	SQAEGGQTPLDAFQL	0.029

>LocNES859122758_0	526-540	TLLHAGFLPPVVRGL	0.074
>LocNES859122758_0	549-563	GPAELGLVYPHLQRL	0.057
>LocNES859122758_0	565-579	PGPALPEAFYPPLGL	0.038

NetNES 1.1 Server - prediction results

```
>Sequence - NetNES 1.1 prediction
```

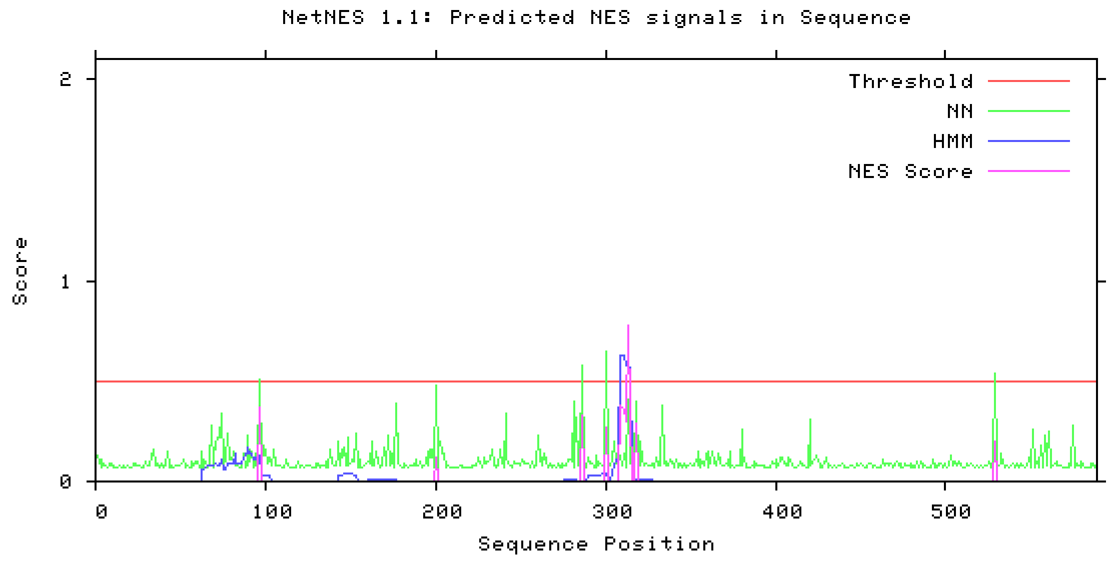

hNPAS2

```
>sp|Q99743|NPAS2_HUMAN Neuronal PAS domain-containing protein 2 OS=Homo
sapiens OX=9606 GN=NPAS2 PE=1 SV=3
MDEDEKDRAKRASRNKSEKKRRDQFNVLIKELSSMLPGNTRKMDKTTVLEKVIGFLQKHN
EVSAQTEICDIQQDWKPSFLSNEEFTQLMLEALDGFIIAVTTDGSIIYVSDSITPLLGHL
PSDVMDQNLLNFLPEQEHSEVYKILSSHMLVTDSPSPEYLKSDSDLEFYCHLLRGSLNPK
EFPTYEYIKFVGNFRSYNNVPSPSCNGFDNTLSRPCRVPLGKEVCFIATVRLATPQFLKE
MCIVDEPLEEFTSRHSLEWKFLFLDHRAPPIIGYLPFEVLGTSGYDYYHIDDLELLARCH
QHLMQFGKGKSCCYRFLTKGQQWIWLQTHYYITYHQWNSKPEFIVCTHSVVSYADVRVER
RQELALEDPPSEALHSSALKDKGSSLEPRQHFNTLDVGASGLNTSHSPSASSRSSHKSSH
TAMSEPTSTPTKLMAEASTPALPRSATLPQELPVPGLSQAATMPAPLPSPSSCDLTQQLL
PQTVLQSTPAPMAQFSAQFSMFQTIKDQLEQRTRILQANIRWQQEELHKIQEQLCLVQDS
NVQMFLQQPAVSLSFSSTQRPEAQQQLQQRSAAVTQPQLGAGPQLPGQISSAQVTSQHLL
RESSVISTQGPKPMRSSQLMQSSGRSGSSLVSPFSSATAALPPSLNLTTPASTSQDASQC
QPSPDFSHDRQLRLLLSQPIQPMMPGSCDARQPSEVSRTGRQVKYAQSQTVFQNPDAHPA
NSSSAPMPVLLMGQAVLHPSFPASQPSPLQPAQARQQPPQHYLQVQAPTSLHSEQQDSLL
LSTYSQQPGTLGYPQPPPAQPQPLRPPRRVSSLSESSGLQQPPR
```

cNLS Mapper Result cut-off score 2.0

Predicted NLSs in query sequence

```
MDEDEKDRAKRASRNKSEKKRRDQFNVLIKELSSMLPGNTRKMDKTTVLE  50
KVIGFLQKHNEVSAQTEICDIQQDWKPSFLSNEEFTQLMLEALDGFIIAV 100
TTDGSIIYVSDSITPLLGHLPSDVMDQNLLNFLPEQEHSEVYKILSSHML 150
VTDSPSPEYLKSDSDLEFYCHLLRGSLNPKEFPTYEYIKFVGNFRSYNNV 200
PSPSCNGFDNTLSRPCRVPLGKEVCFIATVRLATPQFLKEMCIVDEPLEE 250
FTSRHSLEWKFLFLDHRAPPIIGYLPFEVLGTSGYDYYHIDDLELLARCH 300
QHLMQFGKGKSCCYRFLTKGQQWIWLQTHYYITYHQWNSKPEFIVCTHSV 350
VSYADVRVERRQELALEDPPSEALHSSALKDKGSSLEPRQHFNTLDVGAS 400
GLNTSHSPSASSRSSHKSSHTAMSEPTSTPTKLMAEASTPALPRSATLPQ 450
ELPVPGLSQAATMPAPLPSPSSCDLTQQLLPQTVLQSTPAPMAQFSAQFS 500
MFQTIKDQLEQRTRILQANIRWQQEELHKIQEQLCLVQDSNVQMFLQQPA 550
VSLSFSSTQRPEAQQQLQQRSAAVTQPQLGAGPQLPGQISSAQVTSQHLL 600
RESSVISTQGPKPMRSSQLMQSSGRSGSSLVSPFSSATAALPPSLNLTTP 650
ASTSQDASQCQPSPDFSHDRQLRLLLSQPIQPMMPGSCDARQPSEVSRTG 700
RQVKYAQSQTVFQNPDAHPANSSSAPMPVLLMGQAVLHPSFPASQPSPLQ 750
PAQARQQPPQHYLQVQAPTSLHSEQQDSLLLSTYSQQPGTLGYPQPPPAQ 800
PQPLRPPRRVSSLSESSGLQQPPR                           824
```

Predicted monopartite NLS		
**Pos.**	**Sequence**	**Score**

Pos.	Sequence	Score
11	RASRNKSEKKRRDQFNVLIKELSSMLPGNTRKMDK	2.2
11	RASRNKSEKKRRDQFNVLIKELSSMLPGNTRKMDKT	3.6
14	RNKSEKKRRDQFNVLIKELSSMLPGNTRKMDKT	2.7
14	RNKSEKKRRDQFNVLIKELSSMLPGNTRKMDKTT	7.3
14	RNKSEKKRRDQFNVLIKELSSMLPGNTRKMDKTT	3.2
192	GNFRSYNNVPSPSCNGFDNTLSRPCRVPLGK	2
214	RPCRVPLGKEVCFIATVRLATPQFLKEMCI	3.4
227	IATVRLATPQFLKEMCIVDEPLEEFTSRHSLE	2
231	RLATPQFLKEMCIVDEPLEEFTSRHSLEWKFLFLD	3.7
231	RLATPQFLKEMCIVDEPLEEFTSRHSLEWKFLFLD	2.6
379	LKDKGSSLEPRQHFNTLDVGASGLNTSHS	2.1
502	FQTIKDQLEQRTRILQANIRWQQEELHKIQE	2.2
669	DRQLRLLLSQPIQPMMPGSCDARQPSEVSRT	2
698	RTGRQVKYAQSQTVFQNPDAHPANSSSAPMPVL	2.5

(Table title: Predicted bipartite NLS)

NLStradamus

4 - DEKDRAKRASRNKSEKKRRDQFNVLIKELSSMLPGNTRKMDKT - 46

803 - PLRPPRRVSSLSES - 816

821 - QPPR - 824

NucPred

The NucPred score for your sequence is 0.89 (see score help below)

```
  1   MDEDEKDRAKRASRNKSEKKRRDQFNVLIKELSSMLPGNTRKMDKTTVLE    50
 51   KVIGFLQKHNEVSAQTEICDIQQDWKPSFLSNEEFTQLMLEALDGFIIAV   100
101   TTDGSIIYVSDSITPLLGHLPSDVMDQNLLNFLPEQEHSEVYKILSSHML   150
151   VTDSPSPEYLKSDSDLEFYCHLLRGSLNPKEFPTYEYIKFVGNFRSYNNV   200
201   PSPSCNGFDNTLSRPCRVPLGKEVCFIATVRLATPQFLKEMCIVDEPLEE   250
251   FTSRHSLEWKFLFLDHRAPPIIGYLPFEVLGTSGYDYYHIDDLELLARCH   300
301   QHLMQFGKGKSCCYRFLTKGQQWIWLQTHYYITYHQWNSKPEFIVCTHSV   350
351   VSYADVRVERRQELALEDPPSEALHSSALKDKGSSLEPRQHFNTLDVGAS   400
401   GLNTSHSPSASSRSSHKSSHTAMSEPTSTPTKLMAEASTPALPRSATLPQ   450
451   ELPVPGLSQAATMPAPLPSPSSCDLTQQLLPQTVLQSTPAPMAQFSAQFS   500
501   MFQTIKDQLEQRTRILQANIRWQQEELHKIQEQLCLVQDSNVQMFLQQPA   550
551   VSLSFSSTQRPEAQQQLQQRSAAVTQPQLGAGPQLPGQISSAQVTSQHLL   600
601   RESSVISTQGPKPMRSSQLMQSSGRSGSSLVSPFSSATAALPPSLNLTTP   650
651   ASTSQDASQCQPSPDFSHDRQLRLLLSQPIQPMMPGSCDARQPSEVSRTG   700
701   RQVKYAQSQTVFQNPDAHPANSSSAPMPVLLMGQAVLHPSFPASQPSPLQ   750
751   PAQARQQPPQHYLQVQAPTSLHSEQQDSLLLSTYSQQPGTLGYPQPPPAQ   800
801   PQPLRPPRRVSSLSESSGLQQPPR                             824
```

Positively and negatively influencing subsequences are coloured according to the following scale:

(non-nuclear) negative positive (nuclear)

PSORTII

NUCDISC: discrimination of nuclear localization signals

```
      pat4: KKRR (5) at   19
      pat7: none
      bipartite: none
      content of basic residues:   8.4%
      NLS Score: -0.16
```

SeqNLS cut-off 0,5

Prediction result (The predicted NLS(s) are underlined)

Npas2: MDEDEKDRAKRASRNKSE<u>KKRR</u>DQFNVLIKELSSMLPGNTRKMDKTTVLEKVIGFLQKHN

Definition of different colors in predictions

score range	color
0.1-0.3	
0.3-0.5	
0.5-0.7	
0.7-0.8	
0.8-0.86	
0.86-0.89	
>0.89	

The predicted NLS(s) (score cutoff = 0.5)

Protein ID	Predicted NLS	Start	Stop	The highest score of matches within the prediction
Npas2	KKRR	19	22	0.873

ELM cut-off 100 and 50

No NLS positive result

TRG_NES_CRM1_1 7.626e-04 525-539; 526-539

NES Finder

```
Position        NES          Type
==============================
  492        MAQFSAQFSM    2-3-1
  527        LHKIQEQLCL    2-3-1
```

LocNES

Protein Name	Position	Sequence	Score
>LocNES271820630_0	25-39	RRDQFNVLIKELSSM	0.348
>LocNES271820630_0	45-59	RKMDKTTVLEKVIGF	0.027
>LocNES271820630_0	46-60	KMDKTTVLEKVIGFL	0.133
>LocNES271820630_0	86-100	NEEFTQLMLEALDGF	0.373
>LocNES271820630_0	88-102	EFTQLMLEALDGFII	0.398
>LocNES271820630_0	90-104	TQLMLEALDGFIIAV	0.172
>LocNES271820630_0	106-120	TDGSIIYVSDSITPL	0.010
>LocNES271820630_0	141-155	EHSEVYKILSSHMLV	0.018
>LocNES271820630_0	222-236	VPLGKEVCFIATVRL	0.054
>LocNES271820630_0	233-247	TVRLATPQFLKEMCI	0.010
>LocNES271820630_0	234-248	VRLATPQFLKEMCIV	0.019
>LocNES271820630_0	241-255	FLKEMCIVDEPLEEF	0.006
>LocNES271820630_0	267-281	FLDHRAPPIIGYLPF	0.034
>LocNES271820630_0	485-499	PQTVLQSTPAPMAQF	0.044
>LocNES271820630_0	491-505	STPAPMAQFSAQFSM	0.033
>LocNES271820630_0	492-506	TPAPMAQFSAQFSMF	0.029
>LocNES271820630_0	526-540	WQQEELHKIQEQLCL	0.150
>LocNES271820630_0	527-541	QQEELHKIQEQLCLV	0.030
>LocNES271820630_0	545-559	NVQMFLQQPAVSLSF	0.187
>LocNES271820630_0	637-651	PFSSATAALPPSLNL	0.055
>LocNES271820630_0	722-736	HPANSSSAPMPVLLM	0.022

NetNES 1.1 Server - prediction results

>Sequence - NetNES 1.1 prediction

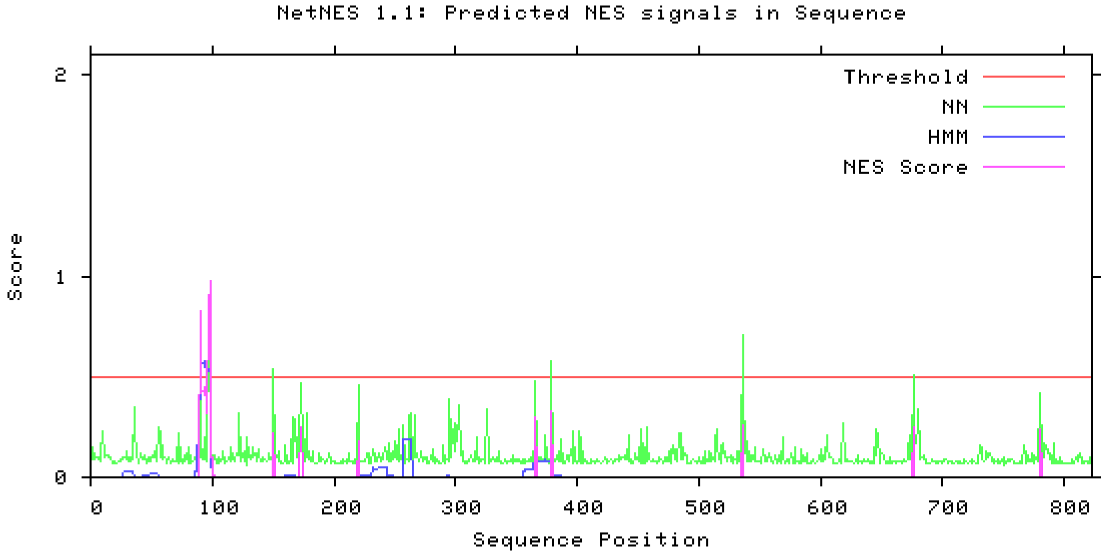

hNPAS3

>sp|Q8IXF0|NPAS3_HUMAN Neuronal PAS domain-containing protein 3 OS=Homo sapiens OX=9606 GN=NPAS3 PE=2 SV=1
MAPTKPSFQQDPSRRERITAQHPLPNQSECRKIYRYDGIYCESTYQNLQALRKEKSRDAA
RSRRGKENFEFYELAKLLPLPAAITSQLDKASIIRLTISYLKMRDFANQGDPPWNLRMEG
PPPNTSVKVIGAQRRRSPSALAIEVFEAHLGSHILQSLDGFVFALNQEGKFLYISETVSI
YLGLSQVELTGSSVFDYVHPGDHVEMAEQLGMKLPPGRGLLSQGTAEDGASSASSSSQSE
TPEPVESTSPSLLTTDNTLERSFFIRMKSTLTKRGVHIKSSGYKVIHITGRLRLRVSLSH
GRTVPSQIMGLVVVAHALPPPTINEVRIDCHMFVTRVNMDLNIIYCENRISDYMDLTPVD
IVGKRCYHFIHAEDVEGIRHSHLDLLNKGQCVTKYYRWMQKNGGYIWIQSSATIAINAKN
ANEKNIIWVNYLLSNPEYKDTPMDIAQLPHLPEKTSESSETSDSESDSKDTSGITEDNEN
SKSDEKGNQSENSEDPEPDRKKSGNACDNDMNCNDDGHSSSNPDSRDSDDSFEHSDFENP
KAGEDGFGALGAMQIKVERYVESESDLRLQNCESLTSDSAKDSDSAGEAGAQASSKHQKR
KKRRKRQKGGSASRRRLSSASSPGGLDAGLVEPPRLLSSPNSASVLKIKTEISEPINFDN
DSSIWNYPPNREISRNESPYSMTKPPSSEHFPSPQGGGGGGGGGGLHVAIPDSVLTPPG
ADGAAARKTQFGASATAALAPVASDPLSPPLSASPRDKHPGNGGGGGGGGGGAGGGGPSA
SNSLLYTGDLEALQRLQAGNVVLPLVHRVTGTLAATSTAAQRVYTTGTIRYAPAEVTLAM
QSNLLPNAHAVNFVDVNSPGFGLDPKTPMEMLYHHVHRLNMSGPFGGAVSAASLTQMPAG
NVFTTAEGLFSTLPFPVYSNGIHAAQTLERKED

cNLS Mapper Result 2.0

Predicted NLSs in query sequence
MAPTKPSFQQDPSRRERITAQHPLPNQSECRKIYRYDGIYCESTYQNLQA 50
LRKEKSRDAARSRRGKENFEFYELAKLLPLPAAITSQLDKASIIRLTISY 100
LKMRDFANQGDPPWNLRMEGPPPNTSVKVIGAQRRRSPSALAIEVFEAHL 150

```
GSHILQSLDGFVFALNQEGKFLYISETVSIYLGLSQVELTGSSVFDYVHP  200
GDHVEMAEQLGMKLPPGRGLLSQGTAEDGASSASSSSQSETPEPVESTSP  250
SLLTTDNTLERSFFIRMKSTLTKRGVHIKSSGYKVIHITGRLRLRVSLSH  300
GRTVPSQIMGLVVVAHALPPPTINEVRIDCHMFVTRVNMDLNIIYCENRI  350
SDYMDLTPVDIVGKRCYHFIHAEDVEGIRHSHLDLLNKGQCVTKYYRWMQ  400
KNGGYIWIQSSATIAINAKNANEKNIIWVNYLLSNPEYKDTPMDIAQLPH  450
LPEKTSESSETSDSESDSKDTSGITEDNENSKSDEKGNQSENSEDPEPDR  500
KKSGNACDNDMNCNDDGHSSSNPDSRDSDDSFEHSDFENPKAGEDGFGAL  550
GAMQIKVERYVESESDLRLQNCESLTSDSAKDSDSAGEAGAQASSKHQKR  600
KKRRKRQKGGSASRRRLSSASSPGGLDAGLVEPPRLLSSPNSASVLKIKT  650
EISEPINFDNDSSIWNYPPNREISRNESPYSMTKPPSSEHFPSPQGGGGG  700
GGGGGGLHVAIPDSVLTPPGADGAAARKTQFGASATAALAPVASDPLSPP  750
LSASPRDKHPGNGGGGGGGGGGAGGGGPSASNSLLYTGDLEALQRLQAGN  800
VVLPLVHRVTGTLAATSTAAQRVYTTGTIRYAPAEVTLAMQSNLLPNAHA  850
VNFVDVNSPGFGLDPKTPMEMLYHHVHRLNMSGPFGGAVSAASLTQMPAG  900
NVFTTAEGLFSTLPFPVYSNGIHAAQTLERKED                   933
```

Predicted monopartite NLS

Pos.	Sequence	Score
596	KHQKRKKRR	2.5
596	KHQKRKKRRK	4
597	HQKRKKRRKR	10
599	KRKKRRKRQ	8
598	QKRKKRRKRQ	6
599	KRKKRRKRQK	10
600	RKKRRKRQKGG	9

Predicted bipartite NLS

Pos.	Sequence	Score
3	PTKPSFQQDPSRRERITAQHPLPNQSECRK	2.1
29	ECRKIYRYDGIYCESTYQNLQALRKEKSRD	3.4
51	LRKEKSRDAARSRRGKENFEFYELAKLLP	2.6
57	RDAARSRRGKENFEFYELAKLLPLPAAITSQLDK	2.2
61	RSRRGKENFEFYELAKLLPLPAAITSQLDKASI	2.3
95	RLTISYLKMRDFANQGDPPWNLRMEGPPPNTSV	2
130	IGAQRRRSPSALAIEVFEAHLGSHILQSLDG	3
261	RSFFIRMKSTLTKRGVHIKSSGYKVIHITGRLRL	2.6
266	RMKSTLTKRGVHIKSSGYKVIHITGRLRLRVSLS	2.2
266	RMKSTLTKRGVHIKSSGYKVIHITGRLRLRVSL	2.7
360	DIVGKRCYHFIHAEDVEGIRHSHLDLLNKG	2.5
374	DVEGIRHSHLDLLNKGQCVTKYYRWMQKNGGYI	2
392	VTKYYRWMQKNGGYIWIQSSATIAINAKN	2
497	EPDRKKSGNACDNDMNCNDDGHSSSNPDSRDS	2
578	DSAKDSDSAGEAGAQASSKHQKRKKRRKRQK	3.9
596	KHQKRKKRRKRQKGGSASRRRLSSASSPGG	2.1
600	RKKRRKRQKGGSASRRRLSSASSPGGLDAGLVE	2.6
606	RQKGGSASRRRLSSASSPGGLDAGLVEPPRLLSSP	4.2
606	RQKGGSASRRRLSSASSPGGLDAGLVEPPRLLSSP	5.1
614	RRRLSSASSPGGLDAGLVEPPRLLSSP	2.3
614	RRRLSSASSPGGLDAGLVEPPRLLSSP	3.3
614	RRRLSSASSPGGLDAGLVEPPRLLSSPNS	2.7
645	VLKIKTEISEPINFDNDSSIWNYPPNREIS	3.2
649	KTEISEPINFDNDSSIWNYPPNREISR	2.7

682<br>727	MTKPPSSEHFPSPQGGGGGGGGGGGLHVAI<br>RKTQFGASATAALAPVASDPLSPPLSASP635	2<br>3.6

NLSstradamus cut-off 0,1

Viterbi Path	596 - KHQKRKKRRKRQKGGSASRRR - 616
Posterior @ 0.1	44 - TYQNLQALRKEKSRDAARSRRGKENFEFYELAKLL - 78
	133 - QRRR - 136
	273 - KR - 274
	582 - DSDSAGEAGAQASSKHQKRKKRRKRQKGGSASRRRLSSASSPGGLDAGLVEPPR - 635
	754 - SPRDKHPGNGGGGGGGGGGA - 773

NucPred

The NucPred score for your sequence is 0.96 (see score help below)

```
  1   MAPTKPSFQQDPSRRERITAQHPLPNQSECRKIYRYDGIYCESTYQNLQA    50
 51   LRKEKSRDAARSRRGKENFEFYELAKLLPLPAAITSQLDKASIIRLTISY   100
101   LKMRDFANQGDPPWNLRMEGPPPNTSVKVIGAQRRRSPSALAIEVFEAHL   150
151   GSHILQSLDGFVFALNQEGKFLYISETVSIYLGLSQVELTGSSVFDYVHP   200
201   GDHVEMAEQLGMKLPPGRGLLSQGTAEDGASSASSSSQSETPEPVESTSP   250
251   SLLTTDNTLERSFFIRMKSTLTKRGVHIKSSGYKVIHITGRLRLRVSLSH   300
301   GRTVPSQIMGLVVVAHALPPPTINEVRIDCHMFVTRVNMDLNIIYCENRI   350
351   SDYMDLTPVDIVGKRCYHFIHAEDVEGIRHSHLDLLNKGQCVTKYYRWMQ   400
401   KNGGYIWIQSSATIAINAKNANEKNIIWVNYLLSNPEYKDTPMDIAQLPH   450
451   LPEKTSESSETSDSESDSKDTSGITEDNENSKSDEKGNQSENSEDPEPDR   500
501   KKSGNACDNDMNCNDDGHSSSNPDSRDSDDSFEHSDFENPKAGEDGFGAL   550
551   GAMQIKVERYVESESDLRLQNCESLTSDSAKDSDSAGEAGAQASSKHQKR   600
601   KKRRKRQKGGSASRRRLSSASSPGGLDAGLVEPPRLLSSPNSASVLKIKT   650
651   EISEPINFDNDSSIWNYPPNREISRNESPYSMTKPPSSEHFPSPQGGGGG   700
701   GGGGGGLHVAIPDSVLTPPGADGAAARKTQFGASATAALAPVASDPLSPP   750
751   LSASPRDKHPGNGGGGGGGGGGAGGGGPSASNSLLYTGDLEALQRLQAGN   800
801   VVLPLVHRVTGTLAATSTAAQRVYTTGTIRYAPAEVTLAMQSNLLPNAHA   850
851   VNFVDVNSPGFGLDPKTPMEMLYHHVHRLNMSGPFGGAVSAASLTQMPAG   900
901   NVFTTAEGLFSTLPFPVYSNGIHAAQTLERKED                    933
```

Positively and negatively influencing subsequences are coloured according to the following scale:

(non-nuclear) negative |||||||||||||||||||||||||||||||||||||||||||||||||||||||||||||||||||||||||||||||||||||||||||||||||||| positive (nuclear)

PSORTII

NUCDISC: discrimination of nuclear localization signals

```
      pat4: KRKK (5) at  599
      pat4: RKKR (5) at  600
      pat4: KKRR (5) at  601
      pat4: KRRK (5) at  602
      pat4: RRKR (5) at  603
      pat7: PSRRERI (4) at   12
      pat7: PEPDRKK (3) at  496
      pat7: PDRKKSG (5) at  498
      bipartite: RKKRRKRQKGGSASRRR at  600
      bipartite: KKRRKRQKGGSASRRRL at  601
      bipartite: KRRKRQKGGSASRRRLS at  602
      content of basic residues:  10.3%
      NLS Score:  3.61
```

SeqNLS

Prediction result (The predicted NLS(s) are underlined)

Npas3: MAPTKPSFQQDPSRRERITAQHPLPNQSECRKIYRYDGIYCESTYQNLQALRKEKSRDAA

Definition of different colors in predictions

score range	color
0.1-0.3	
0.3-0.5	
0.5-0.7	
0.7-0.8	
0.8-0.86	
0.86-0.89	
>0.89	

The predicted NLS(s) (score cutoff = 0.5)

Protein ID	Predicted NLS	Start	Stop	The highest score of matches within the prediction
Npas3	MAPTKPSFQQDPSRRERI	1	18	0.908

ELM cut-off 100

TRG_NLS_MonoCore_2 2.345e-04

QKRKKR 598-603

KRKKRR 599-604

RKKRRK 600-605

KKRRKR 601-606

Monopartite variant of the classical basically charged NLS. Strong core version.

TRG_NLS_MonoExtC_3 7.252e-04

QKRKKR 598-603

KRKKRR 599-604

RKKRRK 600-604

KKRRKR 601-605

RRKRQK 603-608

Monopartite variant of the classical basically charged NLS. C-extended version.

TRG_NLS_MonoExtN_4 1.276e-03

KHQKRKKR 596-603

KRKKRRKR 599-606

RKKRRKR 600-606

KKRRKRQK 601-608

KRRKRQKG 602-609

RRKRQKG 603-609

RKRQKG 604-609

Monopartite variant of the classical basically charged NLS. N-extended version.

No NES positive results

NES Finder

Position NES Type

==============================

71 FYELAKLLPL 2-3-1

94 IRLTISYLKM 3-2-1

285 VIHITGRLRL 2-3-1

334 VTRVNMDLNI 2-3-1

872 LYHHVHRLNM 3-2-1

LocNES

Protein Name	Position	Sequence	Score
>LocNES264943790_0	70-84	KENFEFYELAKLLPL	0.101
>LocNES264943790_0	86-100	AAITSQLDKASIIRL	0.297
>LocNES264943790_0	88-102	ITSQLDKASIIRLTI	0.507
>LocNES264943790_0	91-105	QLDKASIIRLTISYL	0.318
>LocNES264943790_0	93-107	DKASIIRLTISYLKM	0.409
>LocNES264943790_0	153-167	HLGSHILQSLDGFVF	0.057
>LocNES264943790_0	155-169	GSHILQSLDGFVFAL	0.030
>LocNES264943790_0	170-184	NQEGKFLYISETVSI	0.027
>LocNES264943790_0	172-186	EGKFLYISETVSIYL	0.097
>LocNES264943790_0	174-188	KFLYISETVSIYLGL	0.323
>LocNES264943790_0	177-191	YISETVSIYLGLSQV	0.052
>LocNES264943790_0	179-193	SETVSIYLGLSQVEL	0.127
>LocNES264943790_0	202-216	VHPGDHVEMAEQLGM	0.122
>LocNES264943790_0	255-269	SLLTTDNTLERSFFI	0.022
>LocNES264943790_0	284-298	SSGYKVIHITGRLRL	0.266
>LocNES264943790_0	288-302	KVIHITGRLRLRVSL	0.112
>LocNES264943790_0	303-317	SHGRTVPSQIMGLVV	0.020
>LocNES264943790_0	304-318	HGRTVPSQIMGLVVV	0.012
>LocNES264943790_0	318-332	VAHALPPPTINEVRI	0.012
>LocNES264943790_0	329-343	EVRIDCHMFVTRVNM	0.014
>LocNES264943790_0	331-345	RIDCHMFVTRVNMDL	0.014
>LocNES264943790_0	333-347	DCHMFVTRVNMDLNI	0.084
>LocNES264943790_0	334-348	CHMFVTRVNMDLNII	0.043
>LocNES264943790_0	351-365	ENRISDYMDLTPVDI	0.014
>LocNES264943790_0	545-559	KAGEDGFGALGAMQI	0.039
>LocNES264943790_0	547-561	GEDGFGALGAMQIKV	0.030
>LocNES264943790_0	648-662	SVLKIKTEISEPINF	0.059
>LocNES264943790_0	793-807	DLEALQRLQAGNVVL	0.233
>LocNES264943790_0	828-842	YTTGTIRYAPAEVTL	0.013
>LocNES264943790_0	843-857	AMQSNLLPNAHAVNF	0.022
>LocNES264943790_0	846-860	SNLLPNAHAVNFVDV	0.006
>LocNES264943790_0	869-883	PKTPMEMLYHHVHRL	0.038
>LocNES264943790_0	871-885	TPMEMLYHHVHRLNM	0.079
>LocNES264943790_0	903-917	AGNVFTTAEGLFSTL	0.187
>LocNES264943790_0	905-919	NVFTTAEGLFSTLPF	0.136
>LocNES264943790_0	907-921	FTTAEGLFSTLPFPV	0.020

NetNES 1.1 Server - prediction results

>Sequence - NetNES 1.1 prediction

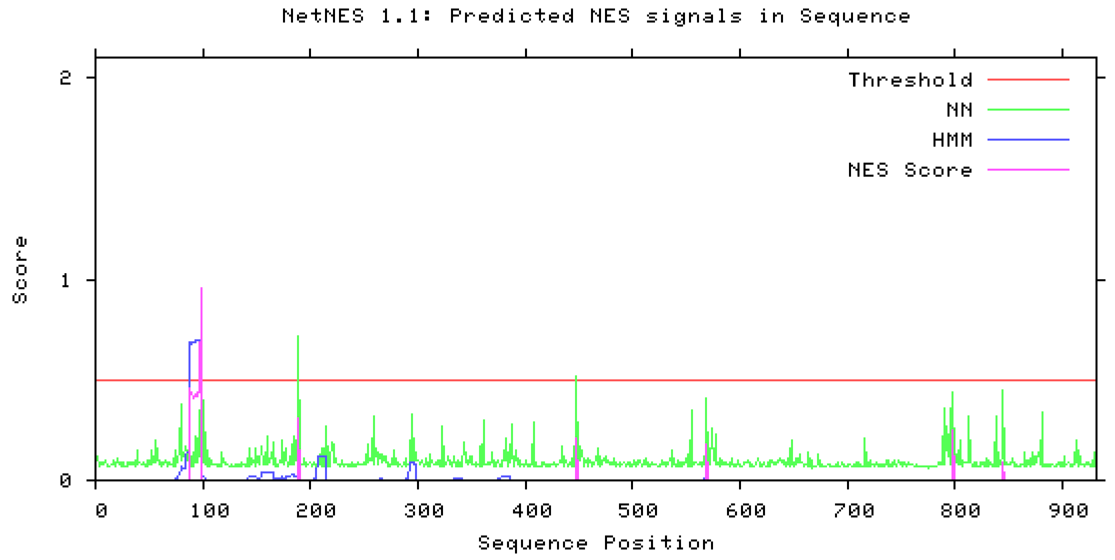

hNPAS4

```
>sp|Q8IUM7|NPAS4_HUMAN Neuronal PAS domain-containing protein 4 OS=Homo
sapiens OX=9606 GN=NPAS4 PE=1 SV=1
MYRSTKGASKARRDQINAEIRNLKELLPLAEADKVRLSYLHIMSLACIYTRKGVFFAGGT
PLAGPTGLLSAQELEDIVAALPGFLLVFTAEGKLLYLSESVSEHLGHSMVDLVAQGDSIY
DIIDPADHLTVRQQLTLPSALDTDRLFRCRFNTSKSLRRQSAGNKLVLIRGRFHAHPPGA
YWAGNPVFTAFCAPLEPRPRPGPGPGPGPASLFLAMFQSRHAKDLALLDISESVLIYLGF
ERSELLCKSWYGLLHPEDLAHASAQHYRLLAESGDIQAEMVVRLQAKTGGWAWIYCLLYS
EGPEGPITANNYPISDMEAWSLRQQLNSEDTQAAYVLGTPTMLPSFPENILSQEECSSTN
PLFTAALGAPRSTSFPSAPELSVVSASEELPRPSKELDFSYLTFPSGPEPSLQAELSKDL
VCTPPYTPHQPGGCAFLFSLHEPFQTHLPTPSSTLQEQLTPSTATFSDQLTPSSATFPDP
LTSPLQGQLTETSVRSYEDQLTPCTSTFPDQLLPSTATFPEPLGSPAHEQLTPPSTAFQA
HLDSPSQTFPEQLSPNPTKTYFAQEGCSFLYEKLPPSPSSPGNGDCTLLALAQLRGPLSV
DVPLVPEGLLTPEASPVKQSFFHYSEKEQNEIDRLIQQISQLAQGMDRPFSAEAGTGGLE
PLGGLEPLDSNLSLSGAGPPVLSLDLKPWKCQELDFLADPDNMFLEETPVEDIFMDLSTP
DPSEEWGSGDPEAEGPGGAPSPCNNLSPEDHSFLEDLATYETAFETGVSAFPYDGFTDEL
HQLQSQVQDSFHEDGSGGEPTF
```

cNLS Mapper Result score cut-off 2.0

Predicted NLSs in query sequence
**MYRSTKGASKARRDQINAEIRNLKELLPLAEADKVRLSYLHIMSLACIYT 50**<br>**RKGVFFAGGTPLAGPTGLLSAQELEDIVAALPGFLLVFTAEGKLLYLSES 100**<br>**VSEHLGHSMVDLVAQGDSIYDIIDPADHLTVRQQLTLPSALDTDRLFRCR 150**

```
FNTSKSLRRQSAGNKLVLIRGRFHAHPPGAYWAGNPVFTAFCAPLEPRPR 200
PGPGPGPGPASLFLAMFQSRHAKDLALLDISESVLIYLGFERSELLCKSW 250
YGLLHPEDLAHASAQHYRLLAESGDIQAEMVVRLQAKTGGWAWIYCLLYS 300
EGPEGPITANNYPISDMEAWSLRQQLNSEDTQAAYVLGTPTMLPSFPENI 350
LSQEECSSTNPLFTAALGAPRSTSFPSAPELSVVSASEELPRPSKELDFS 400
YLTFPSGPEPSLQAELSKDLVCTPPYTPHQPGGCAFLFSLHEPFQTHLPT 450
PSSTLQEQLTPSTATFSDQLTPSSATFPDPLTSPLQGQLTETSVRSYEDQ 500
LTPCTSTFPDQLLPSTATFPEPLGSPAHEQLTPPSTAFQAHLDSPSQTFP 550
EQLSPNPTKTYFAQEGCSFLYEKLPPSPSSPGNGDCTLLALAQLRGPLSV 600
DVPLVPEGLLTPEASPVKQSFFHYSEKEQNEIDRLIQQISQLAQGMDRPF 650
SAEAGTGGLEPLGGLEPLDSNLSLSGAGPPVLSLDLKPWKCQELDFLADP 700
DNMFLEETPVEDIFMDLSTPDPSEEWGSGDPEAEGPGGAPSPCNNLSPED 750
HSFLEDLATYETAFETGVSAFPYDGFTDELHQLQSQVQDSFHEDGSGGEP 800
TF                                                 802
```

Predicted monopartite NLS		
**Pos.**	**Sequence**	**Score**

Predicted bipartite NLS		
**Pos.**	**Sequence**	**Score**
10	KARRDQINAEIRNLKELLPLAEADKVRLSY	2.5
19	EIRNLKELLPLAEADKVRLSYLHIMSLACIYT	2.3
158	RRQSAGNKLVLIRGRFHAHPPGAYWAGNPVFTAF	2.1
220	RHAKDLALLDISESVLIYLGFERSELLCKSW	3.2
283	RLQAKTGGWAWIYCLLYSEGPEGPITANNYPI	2.3
593	QLRGPLSVDVPLVPEGLLTPEASPVKQSF	3

NLSStradamus cut-off 0,1 4HMM static

8 - ASKARRDQI - 16
158 - RRQSAGNKLVLIRGRFHAHPP - 178
193 - APLEPRPRPGPGPGPGPAS - 211

NucPred

The NucPred score for your sequence is 0.51 (see score help below)

```
  1   MYRSTKGASKARRDQINAEIRNLKELLPLAEADKVRLSYLHIMSLACIYT    50
 51   RKGVFFAGGTPLAGPTGLLSAQELEDIVAALPGFLLVFTAEGKLLYLSES   100
101   VSEHLGHSMVDLVAQGDSIYDIIDPADHLTVRQQLTLPSALDTDRLFRCR   150
151   FNTSKSLRRQSAGNKLVLIRGRFHAHPPGAYWAGNPVFTAFCAPLEPRPR   200
201   PGPGPGPGPASLFLAMFQSRHAKDLALLDISESVLIYLGFERSELLCKSW   250
251   YGLLHPEDLAHASAQHYRLLAESGDIQAEMVVRLQAKTGGWAWIYCLLYS   300
```

```
301   EGPEGPITANNYPISDMEAWSLRQQLNSEDTQAAYVLGTPTMLPSFPENI    350
351   LSQEECSSTNPLFTAALGAPRSTSFPSAPELSVVSASEELPRPSKELDFS    400
401   YLTFPSGPEPSLQAELSKDLVCTPPYTPHQPGGCAFLFSLHEPFQTHLPT    450
451   PSSTLQEQLTPSTATFSDQLTPSSATFPDPLTSPLQGQLTETSVRSYEDQ    500
501   LTPCTSTFPDQLLPSTATFPEPLGSPAHEQLTPPSTAFQAHLDSPSQTFP    550
551   EQLSPNPTKTYFAQEGCSFLYEKLPPSPSSPGNGDCTLLALAQLRGPLSV    600
601   DVPLVPEGLLTPEASPVKQSFFHYSEKEQNEIDRLIQQISQLAQGMDRPF    650
651   SAEAGTGGLEPLGGLEPLDSNLSLSGAGPPVLSLDLKPWKCQELDFLADP    700
701   DNMFLEETPVEDIFMDLSTPDPSEEWGSGDPEAEGPGGAPSPCNNLSPED    750
751   HSFLEDLATYETAFETGVSAFPYDGFTDELHQLQSQVQDSFHEDGSGGEP    800
801   TF                                                    802
```

Positively and negatively influencing subsequences are coloured according to the following scale:

(non-nuclear) negative positive (nuclear)

PSORT II

No NLS positive result

SeqNLS cut-0ff 0,5

No predicted NLS

Prediction result (The predicted NLS(s) are underlined)
Npas4: MYRSTKGASKARRDQINAEIRNLKELLPLAEADKVRLSYLHIMSLACIYTRKGVFFAGGT

Definition of different colors in predictions

score range	color
0.1-0.3	
0.3-0.5	
0.5-0.7	
0.7-0.8	
0.8-0.86	
0.86-0.89	
>0.89	

ELM cut-off 50

No NLS positive result

TRG_NES_CRM1_1 QLRGPLSVDVPLVPE 593-607 7.626e-04

NESFinder

Position	NES	Type
20	IRNLKELLPL	2-3-1
591	LAQLRGPLSV	2-3-1
665	LEPLDSNLSL	2-3-1

LocNES

Protein Name	Position	Sequence	Score
>LocNES576996605_0	19-33	QINAEIRNLKELLPL	0.104
>LocNES576996605_0	27-41	LKELLPLAEADKVRL	0.059
>LocNES576996605_0	32-46	PLAEADKVRLSYLHI	0.664
>LocNES576996605_0	35-49	EADKVRLSYLHIMSL	0.198
>LocNES576996605_0	71-85	GLLSAQELEDIVAAL	0.145
>LocNES576996605_0	74-88	SAQELEDIVAALPGF	0.112
>LocNES576996605_0	76-90	QELEDIVAALPGFLL	0.081
>LocNES576996605_0	77-91	ELEDIVAALPGFLLV	0.060
>LocNES576996605_0	78-92	LEDIVAALPGFLLVF	0.130
>LocNES576996605_0	87-101	GFLLVFTAEGKLLYL	0.019
>LocNES576996605_0	121-135	DSIYDIIDPADHLTV	0.151
>LocNES576996605_0	220-234	MFQSRHAKDLALLDI	0.531
>LocNES576996605_0	226-240	AKDLALLDISESVLI	0.118
>LocNES576996605_0	230-244	ALLDISESVLIYLGF	0.343
>LocNES576996605_0	336-350	QAAYVLGTPTMLPSF	0.040
>LocNES576996605_0	373-387	APRSTSFPSAPELSV	0.028
>LocNES576996605_0	394-408	LPRPSKELDFSYLTF	0.143
>LocNES576996605_0	590-604	CTLLALAQLRGPLSV	0.124
>LocNES576996605_0	594-608	ALAQLRGPLSVDVPL	0.030
>LocNES576996605_0	632-646	EQNEIDRLIQQISQL	0.068
>LocNES576996605_0	664-678	EPLGGLEPLDSNLSL	0.602
>LocNES576996605_0	674-688	SNLSLSGAGPPVLSL	0.142
>LocNES576996605_0	676-690	LSLSGAGPPVLSLDL	0.070
>LocNES576996605_0	705-719	DNMFLEETPVEDIFM	0.094

NetNES 1.1 Server - prediction results

```
>Sequence - NetNES 1.1 prediction
```

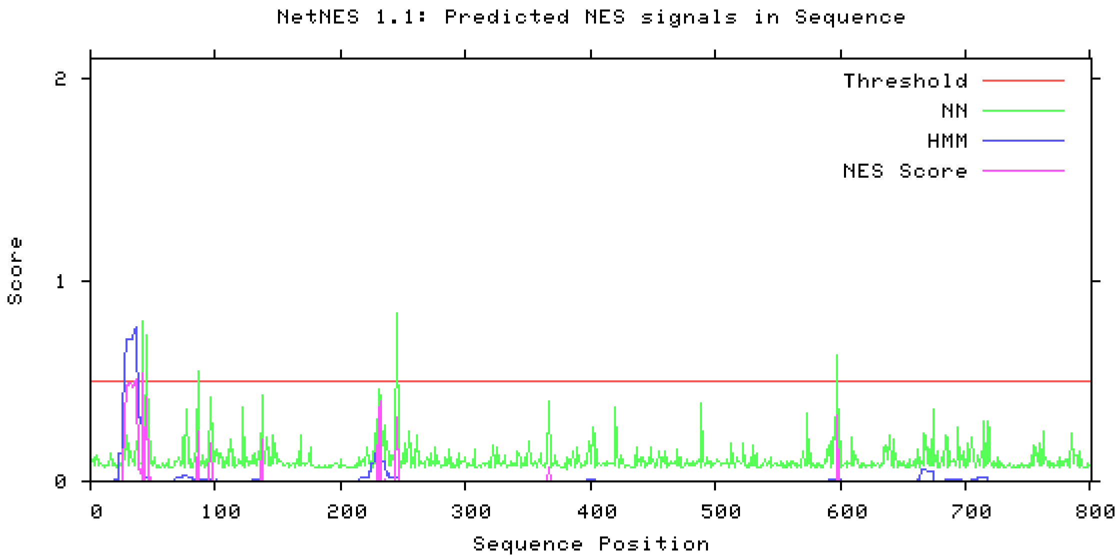

NetNES 1.1: Predicted NES signals in Sequence
Threshold
NN
HMM
NES Score
Score
2
1
0
0
100
200
300
400
500
600
700
800
Sequence Position
