# Supplementary material for: Subcellular Localization Signals of bHLH-PAS Proteins: Their Significance, Current State of Knowledge and Future Perspectives
Source: Int J Mol Sci. 2019 Sep 24;20(19):4746. doi: 10.3390/ijms20194746 (PMC6801399; doi:10.3390/ijms20194746)

Subcellular Localization Signals of bHLH-PAS Proteins: their Significance, Current State of Knowledge and Future Perspectives

Beata Greb-Markiewicz [1,*] and Marta Kolonko [1]

Supplementary Materials Table 2

Predictors used for NLS prediction:
cNLS Mapper
NLSTRADAMUS
NucPred
PSORTII
SeqNLS
ELM

Predictors used for NES prediction:
ELM
NES Finder
NetNES
LocNES

hARNT

```
>sp|P27540|ARNT_HUMAN Aryl hydrocarbon receptor nuclear translocator
OS=Homo sapiens OX=9606 GN=ARNT PE=1 SV=1
MAATTANPEMTSDVPSLGPAIASGNSGPGIQGGGAIVQRAIKRRPGLDFDDDGEGNSKFL
RCDDDQMSNDKERFARSDDEQSSADKERLARENHSEIERRRRNKMTAYITELSDMVPTCS
ALARKPDKLTILRMAVSHMKSLRGTGNTSTDGSYKPSFLTDQELKHLILEAADGFLFIVS
CETGRVVYVSDSVTPVLNQPQSEWFGSTLYDQVHPDDVDKLREQLSTSENALTGRILDLK
TGTVKKEGQQSSMRMCMGSRRSFICRMRCGSSSVDPVSVNRLSFVRNRCRNGLGSVKDGE
PHFVVVHCTGYIKAWPPAGVSLPDDDPEAGQGSKFCLVAIGRLQVTSSPNCTDMSNVCQP
TEFISRHNIEGIFTFVDHRCVATVGYQPQELLGKNIVEFCHPEDQQLLRDSFQQVVKLKG
QVLSVMFRFRSKNQEWLWMRTSSFTFQNPYSDEIEYIICTNTNVKNSSQEPRPTLSNTIQ
RPQLGPTANLPLEMGSGQLAPRQQQQTELDMVPGRDGLASYNHSQVVQPVTTTGPEHSK
PLEKSDGLFAQDRDPRFSEIYHNINADQSKGISSSTVPATQQLFSQGNTFPPTPRPAENF
RNSGLAPPVTIVQPSASAGQMLAQISRHSNPTQGATPTWTPTTRSGFSAQQVATQATAKT
RTSQFGVGSFQTPSSFSSMSLPGAPTASPGAAAYPSLTNRGSNFAPETGQTAGQFQTRTA
EGVGVWPQWQGQQPHHRSSSSEQHVQQPPAQQPGQPEVFQEMLSMLGDQSNSYNNEEFPD
LTMFPPFSE
```

cNLS Mapper Result score cut-off 2.0

Predicted NLSs in query sequence

```
MAATTANPEMTSDVPSLGPAIASGNSGPGIQGGGAIVQRAIKRRPGLDFD  50
DDGEGNSKFLRCDDDQMSNDKERFARSDDEQSSADKERLARENHSEIERR 100
RRNKMTAYITELSDMVPTCSALARKPDKLTILRMAVSHMKSLRGTGNTST 150
DGSYKPSFLTDQELKHLILEAADGFLFIVSCETGRVVYVSDSVTPVLNQP 200
QSEWFGSTLYDQVHPDDVDKLREQLSTSENALTGRILDLKTGTVKKEGQQ 250
SSMRMCMGSRRSFICRMRCGSSSVDPVSVNRLSFVRNRCRNGLGSVKDGE 300
PHFVVVHCTGYIKAWPPAGVSLPDDDPEAGQGSKFCLVAIGRLQVTSSPN 350
CTDMSNVCQPTEFISRHNIEGIFTFVDHRCVATVGYQPQELLGKNIVEFC 400
HPEDQQLLRDSFQQVVKLKGQVLSVMFRFRSKNQEWLWMRTSSFTFQNPY 450
SDEIEYIICTNTNVKNSSQEPRPTLSNTIQRPQLGPTANLPLEMGSGQLA 500
PRQQQQQTELDMVPGRDGLASYNHSQVVQPVTTTGPEHSKPLEKSDGLFA 550
QDRDPRFSEIYHNINADQSKGISSSTVPATQQLFSQGNTFPPTPRPAENF 600
RNSGLAPPVTIVQPSASAGQMLAQISRHSNPTQGATPTWTPTTRSGFSAQ 650
QVATQATAKTRTSQFGVGSFQTPSSFSSMSLPGAPTASPGAAAYPSLTNR 700
GSNFAPETGQTAGQFQTRTAEGVGVWPQWQGQQPHHRSSSSEQHVQQPPA 750
QQPGQPEVFQEMLSMLGDQSNSYNNEEFPDLTMFPPFSE            789
```

Predicted monopartite NLS		
Pos.	Sequence	Score
39	RAIKRRPGLD	5

Predicted bipartite NLS		
Pos.	Sequence	Score
39	RAIKRRPGLDFDDDGEGNSKFLRCD	8.6
39	RAIKRRPGLDFDDDGEGNSKFLRCDDDQM	2.3
39	RAIKRRPGLDFDDDGEGNSKFLRCD	2.5
91	RENHSEIERRRRNKMTAYITELSDMVPTCSALARKP	2.8
96	EIERRRRNKMTAYITELSDMVPTCSALARKPD	2.6
96	EIERRRRNKMTAYITELSDMVPTCSALARKPDK	2.6
98	ERRRRNKMTAYITELSDMVPTCSALARKPD	3.3
99	RRRRNKMTAYITELSDMVPTCSALARKPDKLT	4.6
99	RRRRNKMTAYITELSDMVPTCSALARKPDKLTI	3
151	DGSYKPSFLTDQELKHLILEAADGFLFIVSCE	2.3
235	RILDLKTGTVKKEGQQSSMRMCMGSRRSFICR	2.1
260	RRSFICRMRCGSSSVDPVSVNRLSFVRNRCRNG	2.3
286	RNRCRNGLGSVKDGEPHFVVVHCTGYIKAWPP	2.4
310	GYIKAWPPAGVSLPDDDPEAGQGSKFCLV	2.6
390	ELLGKNIVEFCHPEDQQLLRDSFQQVVKLKG	3.1
409	RDSFQQVVKLKGQVLSVMFRFRSKNQEWLWMRTS	2.2
478	TIQRPQLGPTANLPLEMGSGQLAPRQQQQQT	2.1

NLStradamus cut-off 0,1

Posterior @ 0.1	34 - GAIVQRAIKRRPGL - 47
	83 - SADKERLARENHSEIERRRRNKMTAYITELSDMVPTCSALARKPDK - 128

NucPred

The NucPred score for your sequence is 0.88 (see score help below)

```
  1  MAATTANPEMTSDVPSLGPAIASGNSGPGIQGGGAIVQRAIKRRPGLDFD    50
 51  DDGEGNSKFLRCDDDQMSNDKERFARSDDEQSSADKERLARENHSEIERR   100
101  RRNKMTAYITELSDMVPTCSALARKPDKLTILRMAVSHMKSLRGTGNTST   150
151  DGSYKPSFLTDQELKHLILEAADGFLFIVSCETGRVVYVSDSVTPVLNQP   200
201  QSEWFGSTLYDQVHPDDVDKLREQLSTSENALTGRILDLKTGTVKKEGQQ   250
251  SSMRMCMGSRRSFICRMRCGSSSVDPVSVNRLSFVRNRCRNGLGSVKDGE   300
301  PHFVVVHCTGYIKAWPPAGVSLPDDDPEAGQGSKFCLVAIGRLQVTSSPN   350
351  CTDMSNVCQPTEFISRHNIEGIFTFVDHRCVATVGYQPQELLGKNIVEFC   400
401  HPEDQQLLRDSFQQVVKLKGQVLSVMFRFRSKNQEWLWMRTSSFTFQNPY   450
451  SDEIEYIICTNTNVKNSSQEPRPTLSNTIQRPQLGPTANLPLEMGSGQLA   500
501  PRQQQQQTELDMVPGRDGLASYNHSQVVQPVTTTGPEHSKPLEKSDGLFA   550
551  QDRDPRFSEIYHNINADQSKGISSSTVPATQQLFSQGNTFPPTPRPAENF   600
601  RNSGLAPPVTIVQPSASAGQMLAQISRHSNPTQGATPTWTPTTRSGFSAQ   650
651  QVATQATAKTRTSQFGVGSFQTPSSFSSMSLPGAPTASPGAAAYPSLTNR   700
701  GSNFAPETGQTAGQFQTRTAEGVGVWPQWQGQQPHHRSSSSEQHVQQPPA   750
751  QQPGQPEVFQEMLSMLGDQSNSYNNEEFPDLTMFPPFSE              789
```

Positively and negatively influencing subsequences are coloured according to the following scale:

(non-nuclear) negative positive (nuclear)

PSORTII

NUCDISC: discrimination of nuclear localization signals

```
      pat4: KRRP (4) at   42
      pat4: RRRR (5) at   99
      pat7: none
      bipartite: none
      content of basic residues:   9.4%
      NLS Score:  0.09
```

SeqNLS

Prediction result (The predicted NLS(s) are underlined)

ARNT: MAATTANPEMTSDVPSLGPAIASGNSGPGIQGGGAIVQRAIKRRPGLDFDDDGEGNSKFL

Definition of different colors in predictions

score range	color
0.1-0.3	
0.3-0.5	
0.5-0.7	

0.7-0.8	
0.8-0.86	
0.86-0.89	
>0.89	

The predicted NLS(s) (score cutoff = 0.5)

Protein ID	Predicted NLS	Start	Stop	The highest score of matches within the prediction
ARNT	RAIKRRPGLDFD	39	50	0.820

ELM

TRG_NLS_MonoExtN_4 RAIKRRPG 39-46 Probability 1.276e-03

Monopartite variant of the classical basically charged NLS. N-extended version.

No NES positive result

NESFinder

Position NES Type

==============================

336 CLVAIGRLQV 2-2-1

LocNES

Protein Name	Position	Sequence	Score
>LocNES827787822_0	126-140	LARKPDKLTILRMAV	0.005
>LocNES827787822_0	129-143	KPDKLTILRMAVSHM	0.042
>LocNES827787822_0	132-146	KLTILRMAVSHMKSL	0.041
>LocNES827787822_0	159-173	KPSFLTDQELKHLIL	0.222
>LocNES827787822_0	167-181	ELKHLILEAADGFLF	0.039
>LocNES827787822_0	168-182	LKHLILEAADGFLFI	0.113
>LocNES827787822_0	169-183	KHLILEAADGFLFIV	0.069
>LocNES827787822_0	179-193	FLFIVSCETGRVVYV	0.003
>LocNES827787822_0	186-200	ETGRVVYVSDSVTPV	0.006

>LocNES827787822_0	274-288	GSSSVDPVSVNRLSF	0.308
>LocNES827787822_0	335-349	QGSKFCLVAIGRLQV	0.030
>LocNES827787822_0	408-422	DQQLLRDSFQQVVKL	0.083
>LocNES827787822_0	415-429	SFQQVVKLKGQVLSV	0.079
>LocNES827787822_0	417-431	QQVVKLKGQVLSVMF	0.198
>LocNES827787822_0	436-450	KNQEWLWMRTSSFTF	0.046
>LocNES827787822_0	482-496	TIQRPQLGPTANLPL	0.071
>LocNES827787822_0	484-498	QRPQLGPTANLPLEM	0.086
>LocNES827787822_0	671-685	VGSFQTPSSFSSMSL	0.104
>LocNES827787822_0	715-729	TAGQFQTRTAEGVGV	0.021

NetNES 1.1 Server - prediction results

>sp_P27540_ARNT_HUMAN - NetNES 1.1 prediction

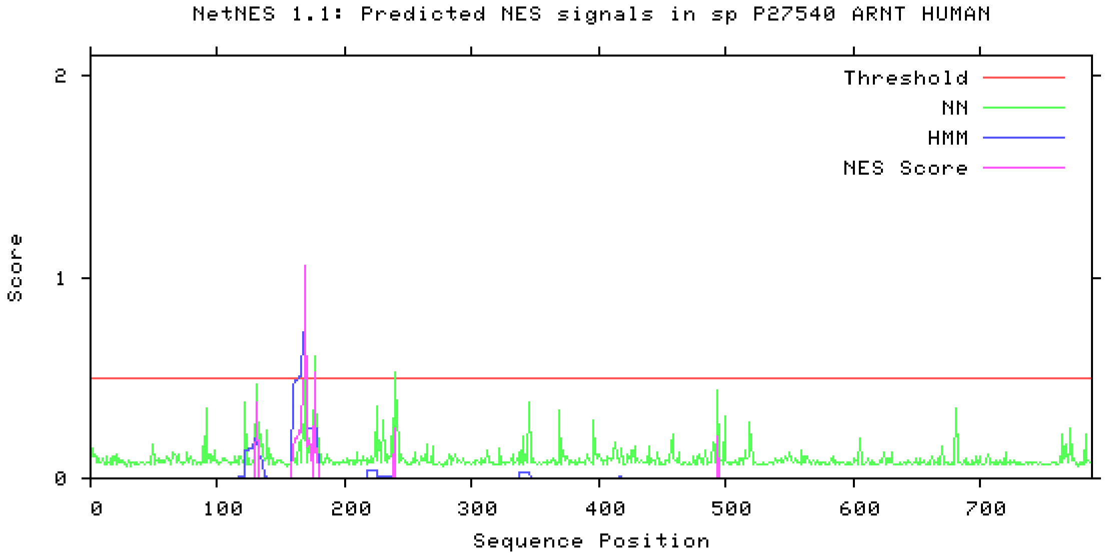

hARNT2

```
>sp|Q9HBZ2|ARNT2_HUMAN Aryl hydrocarbon receptor nuclear translocator 2
OS=Homo sapiens OX=9606 GN=ARNT2 PE=1 SV=2
MATPAAVNPPEMASDIPGSVTLPVAPMAATGQVRMAGAMPARGGKRRSGMDFDDEDGEGP
SKFSRENHSEIERRRRNKMTQYITELSDMVPTCSALARKPDKLTILRMAVSHMKSMRGTG
NKSTDGAYKPSFLTEQELKHLILEAADGFLFVVAAETGRVIYVSDSVTPVLNQPQSEWFG
STLYEQVHPDDVEKLREQLCTSENSMTGRILDLKTGTVKKEGQQSSMRMCMGSRRSFICR
MRCGNAPLDHLPLNRITTMRKRFRNGLGPVKEGEAQYAVVHCTGYIKAWPPAGMTIPEED
ADVGQGSKYCLVAIGRLQVTSSPVCMDMNGMSVPTEFLSRHNSDGIITFVDPRCISVIGY
QPQDLLGKDILEFCHPEDQSHLRESFQQVVKLKGQVLSVMYRFRTKNREWMLIRTSSFTF
QNPYSDEIEYIICTNTNVKQLQQQQAELEVHQRDGLSSYDLSQVPVPNLPAGVHEAGKSV
EKADAIFSQERDPRFAEMFAGISASEKKMMSSASAAGTQQIYSQGSPFPSGHSGKAFSSS
VVHVPGVNDIQSSSSTGQNMSQISRQLNQSQVAWTGSRPPFPGQQIPSQSSKTQSSPFGI
GTSHTYPADPSSYSPLSSPATSSPSGNAYSSLANRTPGFAESGQSSGQFQGRPSEVWSQW
QSQHHGQQSGEQHSHQQPGQTEVFQDMLPMPGDPTQGTGNYNIEDFADLGMFPPFSE
```

cNLS Mapper Result score cut-off 2.0

Predicted NLSs in query sequence

```
MATPAAVNPPEMASDIPGSVTLPVAPMAATGQVRMAGAMPARGGKRRSGM  50
DFDDEDGEGPSKFSRENHSEIERRRRNKMTQYITELSDMVPTCSALARKP  100
DKLTILRMAVSHMKSMRGTGNKSTDGAYKPSFLTEQELKHLILEAADGFL  150
FVVAAETGRVIYVSDSVTPVLNQPQSEWFGSTLYEQVHPDDVEKLREQLC  200
TSENSMTGRILDLKTGTVKKEGQQSSMRMCMGSRRSFICRMRCGNAPLDH  250
LPLNRITTMRKRFRNGLGPVKEGEAQYAVVHCTGYIKAWPPAGMTIPEED  300
ADVGQGSKYCLVAIGRLQVTSSPVCMDMNGMSVPTEFLSRHNSDGIITFV  350
DPRCISVIGYQPQDLLGKDILEFCHPEDQSHLRESFQQVVKLKGQVLSVM  400
YRFRTKNREWMLIRTSSFTFQNPYSDEIEYIICTNTNVKQLQQQQAELEV  450
HQRDGLSSYDLSQVPVPNLPAGVHEAGKSVEKADAIFSQERDPRFAEMFA  500
GISASEKKMMSSASAAGTQQIYSQGSPFPSGHSGKAFSSSVVHVPGVNDI  550
QSSSSTGQNMSQISRQLNQSQVAWTGSRPPFPGQQIPSQSSKTQSSPFGI  600
GTSHTYPADPSSYSPLSSPATSSPSGNAYSSLANRTPGFAESGQSSGQFQ  650
GRPSEVWSQWQSQHHGQQSGEQHSHQQPGQTEVFQDMLPMPGDPTQGTGN  700
YNIEDFADLGMFPPFSE                                   717
```

Predicted monopartite NLS

Pos.	Sequence	Score
42	RGGKRRSGMD	4
42	RGGKRRSGMDF	2.5
256	ITTMRKRFRNG	6

Predicted bipartite NLS

Pos.	Sequence	Score
34	RMAGAMPARGGKRRSGMDFDDEDGEGPSKFSRE	2.1
42	RGGKRRSGMDFDDEDGEGPSKFSRE	10.5
42	RGGKRRSGMDFDDEDGEGPSKFSREN	2.5
42	RGGKRRSGMDFDDEDGEGPSKFSRENHSEI	3.8
42	RGGKRRSGMDFDDEDGEGPSKFSRENHSEIE	3.3
42	RGGKRRSGMDFDDEDGEGPSKFSRE	4.6
42	RGGKRRSGMDFDDEDGEGPSKFSRENHSEIE	2
65	RENHSEIERRRRNKMTQYITELSDMVPTCSALARKP	2.8
70	EIERRRRNKMTQYITELSDMVPTCSALARKPD	2.6
70	EIERRRRNKMTQYITELSDMVPTCSALARKPDK	2.6
72	ERRRRNKMTQYITELSDMVPTCSALARKPD	3.3
73	RRRRNKMTQYITELSDMVPTCSALARKPDKLT	4
73	RRRRNKMTQYITELSDMVPTCSALARKPDKLTI	2.6
209	RILDLKTGTVKKEGQQSSMRMCMGSRRSFICR	2.1
228	RMCMGSRRSFICRMRCGNAPLDHLPLNRITTMRK	2.2
228	RMCMGSRRSFICRMRCGNAPLDHLPLNRITTMR	2
234	RRSFICRMRCGNAPLDHLPLNRITTMRKRFRNG	3.2
255	RITTMRKRFRNGLGPVKEGEAQYAVVHCTGYIK	2.6
255	RITTMRKRFRNGLGPVKEGEAQYAVVHCTGYI	2.9
255	RITTMRKRFRNGLGPVKEGEAQYAVVHCTGYIKAW	2.2

260	RKRFRNGLGPVKEGEAQYAVVHCTGYIKAWPP	2.4
284	GYIKAWPPAGMTIPEEDADVGQGSKYCLV	2.1
364	DLLGKDILEFCHPEDQSHLRESFQQVVKLKG	3.4
383	RESFQQVVKLKGQVLSVMYRFRTKNREWMLIRTS	2
591	SKTQSSPFGIGTSHTYPADPSSYSPLSSP	2.1

NLStradamus cut off 0.1

Posterior @ 0.1	34 - RMAGAMPARGGKRRSGMDFDDEDGEGPSKFSRENHSEIERRRRNKMTQYITE- 85
	95 - ALARKPDK - 102
	258 - TMRKRFRNGLGP - 269

NucPred

The NucPred score for your sequence is 0.89 (see score help below)

```
  1  MATPAAVNPPEMASDIPGSVTLPVAPMAATGQVRMAGAMPARGGKRRSGM   50
 51  DFDDEDGEGPSKFSRENHSEIERRRRNKMTQYITELSDMVPTCSALARKP  100
101  DKLTILRMAVSHMKSMRGTGNKSTDGAYKPSFLTEQELKHLILEAADGFL  150
151  FVVAAETGRVIYVSDSVTPVLNQPQSEWFGSTLYEQVHPDDVEKLREQLC  200
201  TSENSMTGRILDLKTGTVKKEGQQSSMRMCMGSRRSFICRMRCGNAPLDH  250
251  LPLNRITTMRKRFRNGLGPVKEGEAQYAVVHCTGYIKAWPPAGMTIPEED  300
301  ADVGQGSKYCLVAIGRLQVTSSPVCMDMNGMSVPTEFLSRHNSDGIITFV  350
351  DPRCISVIGYQPQDLLGKDILEFCHPEDQSHLRESFQQVVKLKGQVLSVM  400
401  YRFRTKNREWMLIRTSSFTFQNPYSDEIEYIICTNTNVKQLQQQQAELEV  450
451  HQRDGLSSYDLSQVPVPNLPAGVHEAGKSVEKADAIFSQERDPRFAEMFA  500
501  GISASEKKMMSSASAAGTQQIYSQGSPFPSGHSGKAFSSSVVHVPGVNDI  550
551  QSSSSTGQNMSQISRQLNQSQVAWTGSRPPFPGQQIPSQSSKTQSSPFGI  600
601  GTSHTYPADPSSYSPLSSPATSSPSGNAYSSLANRTPGFAESGQSSGQFQ  650
651  GRPSEVWSQWQSQHHGQQSGEQHSHQQPGQTEVFQDMLPMPGDPTQGTGN  700
701  YNIEDFADLGMFPPFSE                                   717
```

Positively and negatively influencing subsequences are coloured according to the following scale:

(non-nuclear) negative positive (nuclear)

PSORT II

NUCDISC: discrimination of nuclear localization signals

```
      pat4: RRRR (5) at    73
      pat7: none
      bipartite: none
      content of basic residues:   9.3%
```

```
NLS Score: -0.16
```

SeqNLS

Prediction result (The predicted NLS(s) are underlined)

Arnt2: MATPAAVNPPEMASDIPGSVTLPVAPMAATGQVRMAGAMPARGGKRRSGMDFDDEDGEGP

Definition of different colors in predictions

score range	color
0.1-0.3	
0.3-0.5	
0.5-0.7	
0.7-0.8	
0.8-0.86	
0.86-0.89	
>0.89	

The predicted NLS(s) (score cutoff = 0.5)

Protein ID	Predicted NLS	Start	Stop	The highest score of matches within the prediction
Arnt2	MPARGGKRRSGMD	39	51	0.883
Arnt2	DIPGSVTLPVAPMAA	15	29	0.862

ELM

TRG_NLS_MonoExtC_3 MRKRFR 259-264 Probability 7.252e-04

Monopartite variant of the classical basically charged NLS. C-extended version.

TRG_NLS_MonoExtN_4 Probability 1.276e-03

RGGKRRSG 42-49

RKRFRN 260-265

Monopartite variant of the classical basically charged NLS. N-extended version.

No positive NES result

NES Finder 0.2

Position NES Type

==============================

310 CLVAIGRLQV 2-2-1

702 NIEDFADLGM 2-2-1

LocNES

No NES candidate

NetNES 1.1 Server - prediction results

>Sequence - NetNES 1.1 prediction

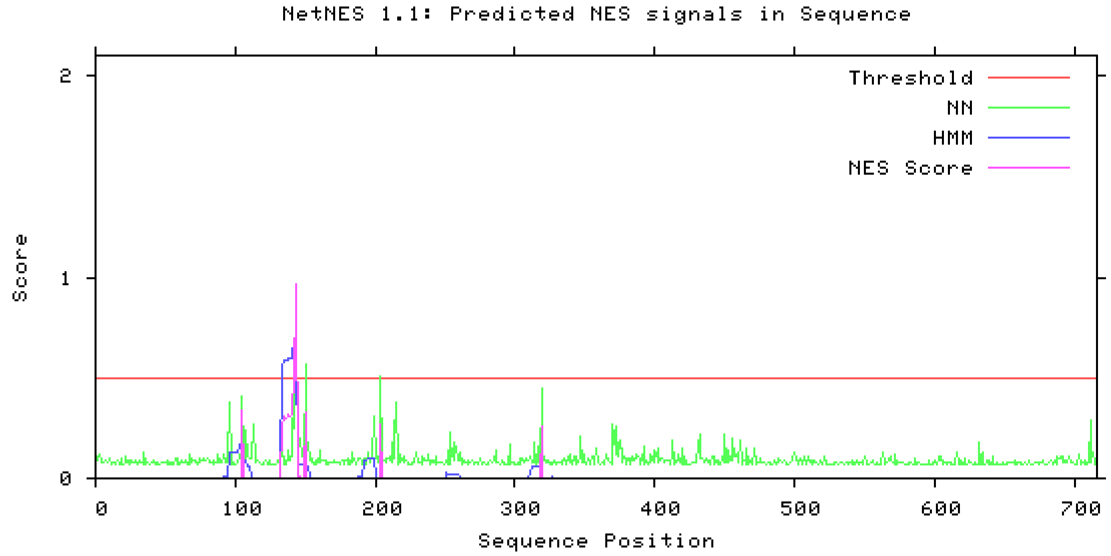

hBMAL1

```
>sp|O00327|BMAL1_HUMAN Aryl hydrocarbon receptor nuclear translocator-like
protein 1 OS=Homo sapiens OX=9606 GN=ARNTL PE=1 SV=2
MADQRMDISSTISDFMSPGPTDLLSSSLGTSGVDCNRKRKGSSTDYQESMDTDKDDPHGR
LEYTEHQGRIKNAREAHSQIEKRRRDKMNSFIDELASLVPTCNAMSRKLDKLTVLRMAVQ
HMKTLRGATNPYTEANYKPTFLSDDELKHLILRAADGFLFVVGCDRGKILFVSESVFKIL
NYSQNDLIGQSLFDYLHPKDIAKVKEQLSSSDTAPRERLIDAKTGLPVKTDITPGPSRLC
SGARRSFFCRMKCNRPSVKVEDKDFPSTCSKKKADRKSFCTIHSTGYLKSWPPTKMGLDE
DNEPDNEGCNLSCLVAIGRLHSHVVPQPVNGEIRVKSMEYVSRHAIDGKFVFVDQRATAI
LAYLPQELLGTSCYEYFHQDDIGHLAECHRQVLQTREKITTNCYKFKIKDGSFITLRSRW
FSFMNPWTKEVEYIVSTNTVVLANVLEGGDPTFPQLTASPHSMDSMLPSGEGGPKRTHPT
VPGIPGGTRAGAGKIGRMIAEEIMEIHRIRGSSPSSCGSSPLNITSTPPPDASSPGGKKI
LNGGTPDIPSSGLLSGQAQENPGYPYSDSSSILGENPHIGIDMIDNDQGSSSPSNDEAAM
AVIMSLLEADAGLGGPVDFSDLPWPL
```

cNLS Mapper Result 2.0

Predicted NLSs in query sequence

```
MADQRMDISSTISDFMSPGPTDLLSSSLGTSGVDCNRKRKGSSTDYQESM  50
DTDKDDPHGRLEYTEHQGRIKNAREAHSQIEKRRRDKMNSFIDELASLVP  100
TCNAMSRKLDKLTVLRMAVQHMKTLRGATNPYTEANYKPTFLSDDELKHL  150
ILRAADGFLFVVGCDRGKILFVSESVFKILNYSQNDLIGQSLFDYLHPKD  200
IAKVKEQLSSSDTAPRERLIDAKTGLPVKTDITPGPSRLCSGARRSFFCR  250
MKCNRPSVKVEDKDFPSTCSKKKADRKSFCTIHSTGYLKSWPPTKMGLDE  300
DNEPDNEGCNLSCLVAIGRLHSHVVPQPVNGEIRVKSMEYVSRHAIDGKF  350
VFVDQRATAILAYLPQELLGTSCYEYFHQDDIGHLAECHRQVLQTREKIT  400
TNCYKFKIKDGSFITLRSRWFSFMNPWTKEVEYIVSTNTVVLANVLEGGD  450
PTFPQLTASPHSMDSMLPSGEGGPKRTHPTVPGIPGGTRAGAGKIGRMIA  500
EEIMEIHRIRGSSPSSCGSSPLNITSTPPPDASSPGGKKILNGGTPDIPS  550
SGLLSGQAQENPGYPYSDSSSILGENPHIGIDMIDNDQGSSSPSNDEAAM  600
AVIMSLLEADAGLGGPVDFSDLPWPL                          626
```

Predicted monopartite NLS

Pos.	Sequence	Score
34	DCNRKRKGSST	5
79	QIEKRRRDKM	5

Predicted bipartite NLS

Pos.	Sequence	Score
34	DCNRKRKGSSTDYQESMDTDKDDPHGRLEYT	4.3
34	DCNRKRKGSSTDYQESMDTDKDDPHGRLEYTE	4.8
37	RKRKGSSTDYQESMDTDKDDPHGRLEYTEH	2.6
74	REAHSQIEKRRRDKMNSFIDELASLVPTCNAMSRK	2.8
74	REAHSQIEKRRRDKMNSFIDELASLVPTCNAMSR	4.3
80	IEKRRRDKMNSFIDELASLVPTCNAMSRKLDK	2.4
81	EKRRRDKMNSFIDELASLVPTCNAMSRKLD	2.6
83	RRRDKMNSFIDELASLVPTCNAMSRKLDKLT	5.4
83	RRRDKMNSFIDELASLVPTCNAMSRKLDKLT	2.1
123	KTLRGATNPYTEANYKPTFLSDDELKHLIL	2.1
174	ESVFKILNYSQNDLIGQSLFDYLHPKDIAKVK	3.1
194	DYLHPKDIAKVKEQLSSSDTAPRERLIDAKTG	2
212	DTAPRERLIDAKTGLPVKTDITPGPSRLCSG	2.3
216	RERLIDAKTGLPVKTDITPGPSRLCSGARRSFF	2
216	RERLIDAKTGLPVKTDITPGPSRLCSGARRSFFC	3.2
227	PVKTDITPGPSRLCSGARRSFFCRMKCNRP	2.6
244	RRSFFCRMKCNRPSVKVEDKDFPSTCSKKKAD	3.3
244	RRSFFCRMKCNRPSVKVEDKDFPSTCSKKKADRK	4.5
244	RRSFFCRMKCNRPSVKVEDKDFPSTCSKKKAD	4
244	RRSFFCRMKCNRPSVKVEDKDFPSTCSKKKADRK	4.6
244	RRSFFCRMKCNRPSVKVEDKDFPSTCSKKKADRKS	3.4
244	RRSFFCRMKCNRPSVKVEDKDFPSTCSKKKADRK	3
250	RMKCNRPSVKVEDKDFPSTCSKKKADRKSFCTI	2.7
268	TCSKKKADRKSFCTIHSTGYLKSWPPTKMG	2.1
272	KKADRKSFCTIHSTGYLKSWPPTKMGLDE	3.4
291	WPPTKMGLDEDNEPDNEGCNLSCLVAIGRL	2.7
291	WPPTKMGLDEDNEPDNEGCNLSCLVAIGRLHS	3
407	KIKDGSFITLRSRWFSFMNPWTKEVEYI	2.5
471	EGGPKRTHPTVPGIPGGTRAGAGKIGRMI	3.7

471	EGGPKRTHPTVPGIPGGTRAGAGKIGRMIAEE	3.8
489	RAGAGKIGRMIAEEIMEIHRIRGSSPSSCGSSP	2.7
535	PGGKKILNGGTPDIPSSGLLSGQAQENPGYP	2.2

NLStradamus cutoff 0,1

37 - RKRKGSSTDYQESMDTDKDDPHGRLEYTEHQGRIKNAREAHSQIEKRRRDKMNSF - 91
258 - VKVEDKDFPSTCSKKKADRKSF - 279
472 - GGPKRTHPTVPGIPGGTRAGAGKIGRMI - 499

NucPred

The NucPred score for your sequence is 0.87 (see score help below)

```
  1   MADQRMDISSTISDFMSPGPTDLLSSSLGTSGVDCNRKRKGSSTDYQESM    50
 51   DTDKDDPHGRLEYTEHQGRIKNAREAHSQIEKRRRDKMNSFIDELASLVP   100
101   TCNAMSRKLDKLTVLRMAVQHMKTLRGATNPYTEANYKPTFLSDDELKHL   150
151   ILRAADGFLFVVGCDRGKILFVSESVFKILNYSQNDLIGQSLFDYLHPKD   200
201   IAKVKEQLSSSDTAPRERLIDAKTGLPVKTDITPGPSRLCSGARRSFFCR   250
251   MKCNRPSVKVEDKDFPSTCSKKKADRKSFCTIHSTGYLKSWPPTKMGLDE   300
301   DNEPDNEGCNLSCLVAIGRLHSHVVPQPVNGEIRVKSMEYVSRHAIDGKF   350
351   VFVDQRATAILAYLPQELLGTSCYEYFHQDDIGHLAECHRQVLQTREKIT   400
401   TNCYKFKIKDGSFITLRSRWFSFMNPWTKEVEYIVSTNTVVLANVLEGGD   450
451   PTFPQLTASPHSMDSMLPSGEGGPKRTHPTVPGIPGGTRAGAGKIGRMIA   500
501   EEIMEIHRIRGSSPSSCGSSPLNITSTPPPDASSPGGKKILNGGTPDIPS   550
551   SGLLSGQAQENPGYPYSDSSSILGENPHIGIDMIDNDQGSSSPSNDEAAM   600
601   AVIMSLLEADAGLGGPVDFSDLPWPL                           626
```

Positively and negatively influencing subsequences are coloured according to the following scale:

(non-nuclear) negative ||||||||||||||||||||||||||||||||||||||||||||||||||||||||||||||||||||||||||||||||||||||||||||||||||||||| positive

PSORT II

NUCDISC: discrimination of nuclear localization signals

```
      pat4: RKRK (5) at   37
      pat4: KRRR (5) at   82
      pat7: none
      bipartite: none
      content of basic residues:  11.7%
      NLS Score:  0.15
```

SeqNLS

Prediction result (The predicted NLS(s) are underlined)

Bmal1: MADQRMDISSTISDFMSPGPTDLLSSSLGTSGVDCNRKRKGSSTDYQESMDTDKDDPHGR

Definition of different colors in predictions

score range	color
0.1-0.3	
0.3-0.5	
0.5-0.7	
0.7-0.8	
0.8-0.86	
0.86-0.89	
>0.89	

The predicted NLS(s) (score cutoff = 0.5)

Protein ID	Predicted NLS	Start	Stop	The highest score of matches within the prediction
Bmal1	LLSSSLGTSGVDCNRKRKGSSTD	23	45	0.879

ELM

TRG_NLS_MonoCore_2 NRKRKG 36-41 Probability 2.345e-04

Monopartite variant of the classical basically charged NLS. Strong core version.

TRG_NLS_MonoExtN_4 RKRKGS 37-42 Probability 1.276e-03

Monopartite variant of the classical basically charged NLS. N-extended version.

No NES positive result

NES Finder

Position NES Type

==============================

105 MSRKLDKLTV 3-2-1

108 KLDKLTVLRM 2-2-1

LocNES

Protein Name	Position	Sequence	Score
>LocNES1322284958_0	88-102	RRDKMNSFIDELASL	0.120
>LocNES1322284958_0	104-118	PTCNAMSRKLDKLTV	0.565
>LocNES1322284958_0	107-121	NAMSRKLDKLTVLRM	0.636
>LocNES1322284958_0	109-123	MSRKLDKLTVLRMAV	0.154
>LocNES1322284958_0	112-126	KLDKLTVLRMAVQHM	0.048
>LocNES1322284958_0	115-129	KLTVLRMAVQHMKTL	0.150
>LocNES1322284958_0	150-164	ELKHLILRAADGFLF	0.021
>LocNES1322284958_0	151-165	LKHLILRAADGFLFV	0.048
>LocNES1322284958_0	152-166	KHLILRAADGFLFVV	0.010
>LocNES1322284958_0	169-183	DRGKILFVSESVFKI	0.006
>LocNES1322284958_0	218-232	APRERLIDAKTGLPV	0.023
>LocNES1322284958_0	325-339	HSHVVPQPVNGEIRV	0.014
>LocNES1322284958_0	432-446	TKEVEYIVSTNTVVL	0.028
>LocNES1322284958_0	609-623	SLLEADAGLGGPVDF	0.013

NetNES 1.1 Server - prediction results

>Sequence - NetNES 1.1 prediction

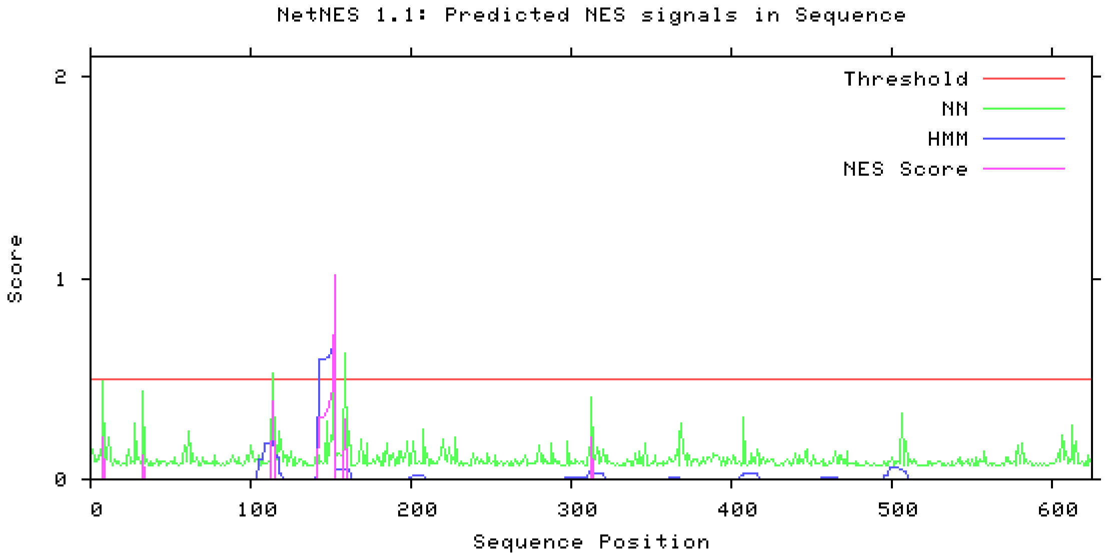

hBMAL2

```
>sp|Q8WYA1|BMAL2_HUMAN Aryl hydrocarbon receptor nuclear translocator-like
protein 2 OS=Homo sapiens OX=9606 GN=ARNTL2 PE=1 SV=2
MAAEEEAAAGGKVLREENQCIAPVVSSRVSPGTRPTAMGSFSSHMTEFPRKRKGSDSDPS
QSGIMTEKVVEKLSQNPLTYLLSTRIEISASSGSRVEDGEHQVKMKAFREAHSQTEKRRR
DKMNNLIEELSAMIPQCNPMARKLDKLTVLRMAVQHLRSLKGLTNSYVGSNYRPSFLQDN
ELRHLILKTAEGFLFVVGCERGKILFVSKSVSKILNYDQASLTGQSLFDFLHPKDVAKVK
EQLSSFDISPREKLIDAKTGLQVHSNLHAGRTRVYSGSRRSFFCRIKSCKISVKEEHGCL
PNSKKKEHRKFYTIHCTGYLRSWPPNIVGMEEERNSKKDNSNFTCLVAIGRLQPYIVPQN
SGEINVKPTEFITRFAVNGKFVYVDQRATAILGYLPQELLGTSCYEYFHQDDHNNLTDKH
```

KAVLQSKEKILTDSYKFRAKDGSFVTLKSQWFSFTNPWTKELEYIVSVNTLVLGHSEPGE
ASFLPCSSQSSEESSRQSCMSVPGMSTGTVLGAGSIGTDIANEILDLQRLQSSSYLDDSS
PTGLMKDTHTVNCRSMSNKELFPPSPSEMGELEATRQNQSTVAVHSHEPLLSDGAQLDFD
ALCDNDDTAMAAFMNYLEAEGGLGDPGDFSDIQWTL

cNLS Mapper Result 2.0

Predicted NLSs in query sequence	
MAAEEEAAAGGKVLREENQCIAPVVSSRVSPGTRPTAMGSFSSHMTEFPR	50
KRKGSDSDPSQSGIMTEKVVEKLSQNPLTYLLSTRIEISASSGSRVEDGE	100
HQVKMKAFREAHSQTEKRRRDKMNNLIEELSAMIPQCNPMARKLDKLTVL	150
RMAVQHLRSLKGLTNSYVGSNYRPSFLQDNELRHLILKTAEGFLFVVGCE	200
RGKILFVSKSVSKILNYDQASLTGQSLFDFLHPKDVAKVKEQLSSFDISP	250
REKLIDAKTGLQVHSNLHAGRTRVYSGSRRSFFCRIKSCKISVKEEHGCL	300
PNSKKKEHRKFYTIHCTGYLRSWPPNIVGMEEERNSKKDNSNFTCLVAIG	350
RLQPYIVPQNSGEINVKPTEFITRFAVNGKFVYVDQRATAILGYLPQELL	400
GTSCYEYFHQDDHNNLTDKHKAVLQSKEKILTDSYKFRAKDGSFVTLKSQ	450
WFSFTNPWTKELEYIVSVNTLVLGHSEPGEASFLPCSSQSSEESSRQSCM	500
SVPGMSTGTVLGAGSIGTDIANEILDLQRLQSSSYLDDSSPTGLMKDTHT	550
VNCRSMSNKELFPPSPSEMGELEATRQNQSTVAVHSHEPLLSDGAQLDFD	600
ALCDNDDTAMAAFMNYLEAEGGLGDPGDFSDIQWTL	636

Predicted monopartite NLS

Pos.	Sequence	Score
47	EFPRKRKGSDS	7
114	QTEKRRRDKM	6

Predicted bipartite NLS

Pos.	Sequence	Score
27	SRVSPGTRPTAMGSFSSHMTEFPRKRKGS	2.3
47	EFPRKRKGSDSDPSQSGIMTEKVVEKLSQNP	2.2
47	EFPRKRKGSDSDPSQSGIMTEKVVEKLS	3.3
47	EFPRKRKGSDSDPSQSGIMTEKVVEKLSQNP	5.5
47	EFPRKRKGSDSDPSQSGIMTEKVVEKLSQNP	4
50	RKRKGSDSDPSQSGIMTEKVVEKLSQNPLTY	2.9
109	REAHSQTEKRRRDKMNNLIEELSAMIPQCNPMA	2.1
109	REAHSQTEKRRRDKMNNLIEELSAMIPQCNPMAR	5.3
115	TEKRRRDKMNNLIEELSAMIPQCNPMARKLD	2.4
116	EKRRRDKMNNLIEELSAMIPQCNPMARKLDK	2.8
116	EKRRRDKMNNLIEELSAMIPQCNPMARKLD	3.3
118	RRRDKMNNLIEELSAMIPQCNPMARKLDKLT	5.4
158	RSLKGLTNSYVGSNYRPSFLQDNELRHLILK	2.2
183	RHLILKTAEGFLFVVGCERGKILFVSKSVSKI	2.1
209	KSVSKILNYDQASLTGQSLFDFLHPKDVAKVK	3.7
235	DVAKVKEQLSSFDISPREKLIDAKTGLQVHSN	2.3
251	REKLIDAKTGLQVHSNLHAGRTRVYSGSRRSFFC	2.2
271	RTRVYSGSRRSFFCRIKSCKISVKEEHGCLPNSKKK	2
279	RRSFFCRIKSCKISVKEEHGCLPNSKKKEHRKFY	2.3

279	RRSFFCRIKSCKISVKEEHGCLPNSKKKEHRKFYTI	3.3
285	RIKSCKISVKEEHGCLPNSKKKEHRKFYTIHCT	2.2
444	FVTLKSQWFSFTNPWTKELEYIVSVNTLVLG	2.2
554	RSMSNKELFPPSPSEMGELEATRQNQSTVAVHS	2.6

NLStradamus cutoff 0.1

Posterior @ 0.1	35 - PTAMGSFSSHMTEFPRKRKGSDSDPS - 60
	103 - VKMKAFREAHSQTEKRRRDKMNNLI - 127
	285 - RIKSCKISVKEEHGCLPNSKKKEHRKFYTIHCTGYLRS - 322

NucPred

The NucPred score for your sequence is 0.85 (see score help below)

```
  1  MAAEEEAAAGGKVLREENQCIAPVVSSRVSPGTRPTAMGSFSSHMTEFPR   50
 51  KRKGSDSDPSQSGIMTEKVVEKLSQNPLTYLLSTRIEISASSGSRVEDGE  100
101  HQVKMKAFREAHSQTEKRRRDKMNNLIEELSAMIPQCNPMARKLDKLTVL  150
151  RMAVQHLRSLKGLTNSYVGSNYRPSFLQDNELRHLILKTAEGFLFVVGCE  200
201  RGKILFVSKSVSKILNYDQASLTGQSLFDFLHPKDVAKVKEQLSSFDISP  250
251  REKLIDAKTGLQVHSNLHAGRTRVYSGSRRSFFCRIKSCKISVKEEHGCL  300
301  PNSKKKEHRKFYTIHCTGYLRSWPPNIVGMEEERNSKKDNSNFTCLVAIG  350
351  RLQPYIVPQNSGEINVKPTEFITRFAVNGKFVYVDQRATAILGYLPQELL  400
401  GTSCYEYFHQDDHNNLTDKHKAVLQSKEKILTDSYKFRAKDGSFVTLKSQ  450
451  WFSFTNPWTKELEYIVSVNTLVLGHSEPGEASFLPCSSQSSEESSRQSCM  500
501  SVPGMSTGTVLGAGSIGTDIANEILDLQRLQSSSYLDDSSPTGLMKDTHT  550
551  VNCRSMSNKELFPPSPSEMGELEATRQNQSTVAVHSHEPLLSDGAQLDFD  600
601  ALCDNDDTAMAAFMNYLEAEGGLGDPGDFSDIQWTL                636
```

Positively and negatively influencing subsequences are coloured according to the following scale:

(non-nuclear) negative |||||||||||||||||||||||||||||||||||||||||||||||||||||||||||||||||||||||| positive (nuclear)

PSORT II

```
NUCDISC: discrimination of nuclear localization signals
      pat4: PRKR (4) at   49
      pat4: RKRK (5) at   50
      pat4: KRRR (5) at  117
      pat7: PRKRKGS (5) at   49
      pat7: PNSKKKE (4) at  301
      bipartite: none
      content of basic residues:  11.9%
      NLS Score:  1.18
```

SeqNLS

Prediction result (The predicted NLS(s) are underlined)

Bmal2: MAAEEEAAAGGKVLREENQCIAPVVSSRVSPGTRPTAMGSFSSHMTEFPRKRKGSDSDPS

Definition of different colors in predictions

score range	color
0.1-0.3	
0.3-0.5	
0.5-0.7	
0.7-0.8	
0.8-0.86	
0.86-0.89	
>0.89	

The predicted NLS(s) (score cutoff = 0.5)

Protein ID	Predicted NLS	Start	Stop	The highest score of matches within the prediction
Bmal2	HMTEFPRKRKGSDSDP	44	59	0.895
Bmal2	MAAEEEAAAG	1	10	0.893
Bmal2	QCIAPVVS	19	26	0.791

ELM

TRG_NLS_MonoCore_2 PRKRKG 49-54 Probability 2.345e-04

Monopartite variant of the classical basically charged NLS. Strong core version.

TRG_NLS_MonoExtC_3 RKRKGSD 50-56 Probability 7.252e-04

Monopartite variant of the classical basically charged NLS. C-extended version.

TRG_NLS_MonoExtN_4 1.276e-03

PRKRKGS 49-55

RKRKGS 50-55

Monopartite variant of the classical basically charged NLS. N-extended version.

TRG_NES_CRM1_1 463-477 Probability 7.626e-04

Some proteins re-exported from the nucleus contain a Leucine-rich nuclear export signal (NES) binding to the CRM1 exportin protein.

NES Finder

Position NES Type

==============================

140 MARKLDKLTV 3-2-1

143	KLDKLTVLRM	2-2-1
239	VKEQLSSFDI	3-2-1

LocNES

Protein Name	Position	Sequence	Score
>LocNES684481929_0	38-52	RPTAMGSFSSHMTEF	0.041
>LocNES684481929_0	78-92	SQNPLTYLLSTRIEI	0.075
>LocNES684481929_0	123-137	RRDKMNNLIEELSAM	0.041
>LocNES684481929_0	139-153	PQCNPMARKLDKLTV	0.316
>LocNES684481929_0	142-156	NPMARKLDKLTVLRM	0.614
>LocNES684481929_0	144-158	MARKLDKLTVLRMAV	0.121
>LocNES684481929_0	147-161	KLDKLTVLRMAVQHL	0.224
>LocNES684481929_0	150-164	KLTVLRMAVQHLRSL	0.215
>LocNES684481929_0	185-199	ELRHLILKTAEGFLF	0.015
>LocNES684481929_0	186-200	LRHLILKTAEGFLFV	0.036
>LocNES684481929_0	187-201	RHLILKTAEGFLFVV	0.008
>LocNES684481929_0	204-218	ERGKILFVSKSVSKI	0.008
>LocNES684481929_0	236-250	HPKDVAKVKEQLSSF	0.210
>LocNES684481929_0	238-252	KDVAKVKEQLSSFDI	0.225
>LocNES684481929_0	253-267	SPREKLIDAKTGLQV	0.036
>LocNES684481929_0	367-381	EINVKPTEFITRFAV	0.013
>LocNES684481929_0	458-472	FTNPWTKELEYIVSV	0.069
>LocNES684481929_0	463-477	TKELEYIVSVNTLVL	0.084

NetNES 1.1 Server - prediction results

```
>Sequence - NetNES 1.1 prediction
```

NetNES 1.1: Predicted NES signals in Sequence

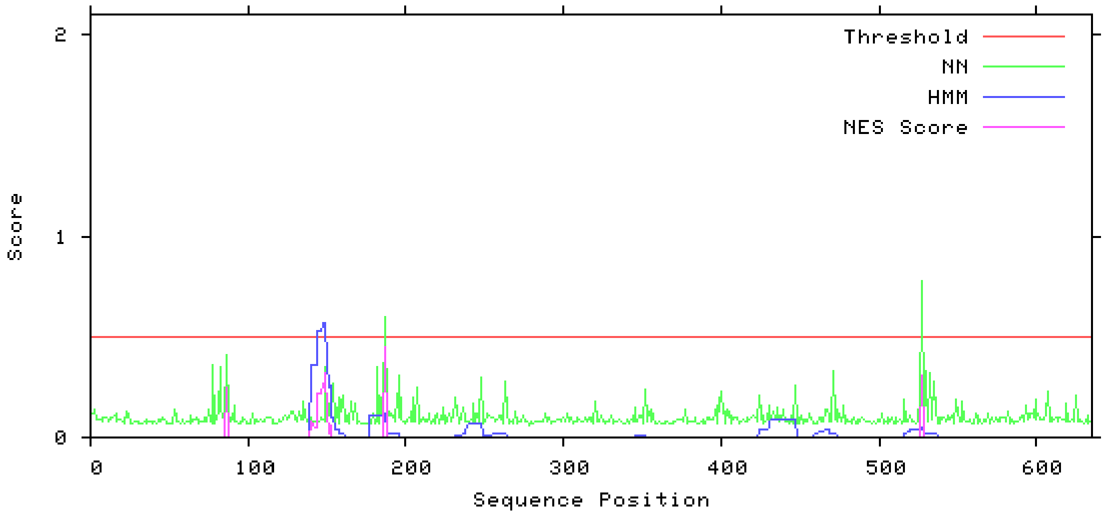

Supplement: Supplementary file 1 [file ijms-20-04746-s001.zip › SupplementaryIJMS/Supplementary materials2.pdf]
