# Supplementary material for: Subcellular Localization Signals of bHLH-PAS Proteins: Their Significance, Current State of Knowledge and Future Perspectives
Source: Int J Mol Sci. 2019 Sep 24;20(19):4746. doi: 10.3390/ijms20194746 (PMC6801399; doi:10.3390/ijms20194746)

Subcellular Localization Signals of bHLH-PAS Proteins: their Significance, Current State of Knowledge and Future Perspectives

Beata Greb-Markiewicz [1,*] and Marta Kolonko [1]

Supplementary Materials Table 3

Predictors used for NLS prediction:
cNLS Mapper
NLSTRADAMUS
NucPred
PSORTII
SeqNLS
ELM

Predictors used for NES prediction:
ELM
NES Finder
NetNES
LocNES

MET

```
>tr|Q9VYW2|Q9VYW2_DROME FI10506p OS=Drosophila melanogaster OX=7227 GN=Met
PE=2 SV=1
MAAPETGNTGSTGSAGSTGSGSGSGSGSGSSSDPANGREARNLAEKQRRDKLNASIQELA
TMVPHAAESSRRLDKTAVLRFATHGLRLQYVFGKSASRRRKKTGLKGTGMSASPVGDLPN
PSLHLTDTLMQLLDCCFLTLTCSGQIVLVSTSVEQLLGHCQSDLYGQNLLQITHPDDQDL
LRQQLIPRDIETLFYQHQHHQQQGHNPQQHSTSTSASTSGSDLEEEEMETEEHRLGRQQG
EADDDEDHPYNRRTPSPRRMAHLATIDDRLRMDRRCFTVRLARASTRAEATRHYERVKID
GCFRRSDSSLTGGAAANYPIVSQLIRRSRNNNMLAAAAAVAAEAATVPPQHDAIAQAALH
GISGNDIVLVAMARVLREERPPEETEGTVGLTIYRQPEPYQLEYHTRHLIDGSIIDCDQR
IGLVAGYMKDEVRNLSPFCFMHLDDVRWVIVALRQMYDCNSDYGESCYRLLSRNGRFIYL
HTKGFLEVDRGSNKVHSFLCVNTLLDEEAGRQKVQEMKEKFSTIIKAEMPTQSSSPDLPA
SQAPQQLERIVLYLIENLQKSVDSAETVGGQGMESLMDDGYSSPANTLTLEELAPSPTPA
LALVPPAPSSVKSSISKSVSVVNVTAARKFQQEHQKQRERDREQLKERTNSTQGVIRQLS
SCLSEAETASCILSPASSLSASEAPDTPDPHSNTSPPPSLHTRPSVLHRTLTSTLR
```

cNLS Mapper Result score 2.0

Predicted NLSs in query sequence

```
MAAPETGNTGSTGSAGSTGSGSGSGSGSGSSSDPANGREARNLAEKQRRD  50
KLNASIQELATMVPHAAESSRRLDKTAVLRFATHGLRLQYVFGKSASRRR  100
KKTGLKGTGMSASPVGDLPNPSLHLTDTLMQLLDCCFLTLTCSGQIVLVS  150
TSVEQLLGHCQSDLYGQNLLQITHPDDQDLLRQQLIPRDIETLFYQHQHH  200
QQQGHNPQQHSTSTSASTSGSDLEEEEMETEEHRLGRQQGEADDDEDHPY  250
NRRTPSPRRMAHLATIDDRLRMDRRCFTVRLARASTRAEATRHYERVKID  300
GCFRRSDSSLTGGAAANYPIVSQLIRRSRNNNMLAAAAAVAAEAATVPPQ  350
HDAIAQAALHGISGNDIVLVAMARVLREERPPEETEGTVGLTIYRQPEPY  400
QLEYHTRHLIDGSIIDCDQRIGLVAGYMKDEVRNLSPFCFMHLDDVRWVI  450
VALRQMYDCNSDYGESCYRLLSRNGRFIYLHTKGFLEVDRGSNKVHSFLC  500
VNTLLDEEAGRQKVQEMKEKFSTIIKAEMPTQSSSPDLPASQAPQQLERI  550
VLYLIENLQKSVDSAETVGGQGMESLMDDGYSSPANTLTLEELAPSPTPA  600
LALVPPAPSSVKSSISKSVSVVNVTAARKFQQEHQKQRERDREQLKERTN  650
STQGVIRQLSSCLSEAETASCILSPASSLSASEAPDTPDPHSNTSPPPSL  700
HTRPSVLHRTLTSTLR                                    716
```

Predicted monopartite NLS		
**Pos.**	**Sequence**	**Score**

Predicted bipartite NLS		
**Pos.**	**Sequence**	**Score**
38	REARNLAEKQRRDKLNASIQELATMVPHAAESSRR	2.9
41	RNLAEKQRRDKLNASIQELATMVPHAAESSRRLDKT	2.7
48	RRDKLNASIQELATMVPHAAESSRRLDKTA	2.1
71	RRLDKTAVLRFATHGLRLQYVFGKSASRRRKK	3.6
98	RRRKKTGLKGTGMSASPVGDLPNPSLHLT	3.2
98	RRRKKTGLKGTGMSASPVGDLPNPSLHLTD	2.1
98	RRRKKTGLKGTGMSASPVGDLPNPSLHLTDT	2.5
98	RRRKKTGLKGTGMSASPVGDLPNPSLHLTDT	3.8
232	EHRLGRQQGEADDDEDHPYNRRTPSPRRMAHL	3.3
247	DHPYNRRTPSPRRMAHLATIDDRLRMDRRCFT	2
251	NRRTPSPRRMAHLATIDDRLRMDRRCFTV	2
252	RRTPSPRRMAHLATIDDRLRMDRRCFTVRLARAS	3.9
252	RRTPSPRRMAHLATIDDRLRMDRRCFTVRLARAST	2.2
296	RVKIDGCFRRSDSSLTGGAAANYPIVSQLIRRSRN	3.5
447	RWVIVALRQMYDCNSDYGESCYRLLSRNGRFIYL	2.2
489	DRGSNKVHSFLCVNTLLDEEAGRQKVQEMK	2
489	DRGSNKVHSFLCVNTLLDEEAGRQKVQEMKE	3
516	EMKEKFSTIIKAEMPTQSSSPDLPASQAPQ	2
523	TIIKAEMPTQSSSPDLPASQAPQQLERIVLY	2.7
628	RKFQQEHQKQRERDREQLKERTNSTQGVI	2
628	RKFQQEHQKQRERDREQLKERTNSTQGVIRQLSSC	2.6

NLStradamus cutoff 0,1

38 - REARNLAEKQRRDKL - 52
68 - ESSRRLDKTAVLRFATHGLRLQYVFGKSASRRRKKTGLKGTGMSASPV - 115

252 - RRTPSPRRM - 260	
636 - KQRER - 640	

NucPred

The NucPred score for your sequence is 0.82 (see score help below)

```
  1   MAAPETGNTGSTGSAGSTGSGSGSGSGSGSSSDPANGREARNLAEKQRRD    50
 51   KLNASIQELATMVPHAAESSRRLDKTAVLRFATHGLRLQYVFGKSASRRR   100
101   KKTGLKGTGMSASPVGDLPNPSLHLTDTLMQLLDCCFLTLTCSGQIVLVS   150
151   TSVEQLLGHCQSDLYGQNLLQITHPDDQDLLRQQLIPRDIETLFYQHQHH   200
201   QQQGHNPQQHSTSTSASTSGSDLEEEEMETEEHRLGRQQGEADDDEDHPY   250
251   NRRTPSPRRMAHLATIDDRLRMDRRCFTVRLARASTRAEATRHYERVKID   300
301   GCFRRSDSSLTGGAAANYPIVSQLIRRSRNNNMLAAAAAVAAEAATVPPQ   350
351   HDAIAQAALHGISGNDIVLVAMARVLREERPPEETEGTVGLTIYRQPEPY   400
401   QLEYHTRHLIDGSIIDCDQRIGLVAGYMKDEVRNLSPFCFMHLDDVRWVI   450
451   VALRQMYDCNSDYGESCYRLLSRNGRFIYLHTKGFLEVDRGSNKVHSFLC   500
501   VNTLLDEEAGRQKVQEMKEKFSTIIKAEMPTQSSSPDLPASQAPQQLERI   550
551   VLYLIENLQKSVDSAETVGGQGMESLMDDGYSSPANTLTLEELAPSPTPA   600
601   LALVPPAPSSVKSSISKSVSVVNVTAARKFQQEHQKQRERDREQLKERTN   650
651   STQGVIRQLSSCLSEAETASCILSPASSLSASEAPDTPDPHSNTSPPPSL   700
701   HTRPSVLHRTLTSTLR                                     716
```

Positively and negatively influencing subsequences are coloured according to the following scale:

(non-nuclear) negative positive (nuclear)

PSORTII

NUCDISC: discrimination of nuclear localization signals

```
      pat4: RRRK (5) at    98
      pat4: RRKK (5) at    99
      pat7: none
      bipartite: none
      content of basic residues:  10.9%
      NLS Score:  0.15
```

SeqNLS

Final-score cutoff: 0.5

Prediction result (The predicted NLS(s) are underlined)

Met: MAAPETGNTGSTGSAGSTGSGSGSGSGSGSSSDPANGREARNLAEKQRRDKLNASIQELA

Definition of different colors in predictions

score range	color
0.1-0.3	
0.3-0.5	

0.5-0.7	
0.7-0.8	
0.8-0.86	
0.86-0.89	
>0.89	

The predicted NLS(s) (score cutoff = 0.5)

Protein ID	Predicted NLS	Start	Stop	The highest score of matches within the prediction
Met	PANGREARNL	34	43	0.548

ELM

TRG_NLS_MonoExtN_4 Probability 1.276e-03

RRRKKTG 98-104

RRKKTG 99-104

Monopartite variant of the classical basically charged NLS. N-extended version.

No NES positive result

NES Finder

Position	NES	Type
263	LATIDDRLRM	2-3-1
431	EVRNLSPFCF	2-2-1

LocNES

Protein Name	Position	Sequence	Score
>LocNES219057319_0	71-85	AESSRRLDKTAVLRF	0.096
>LocNES219057319_0	78-92	DKTAVLRFATHGLRL	0.201
>LocNES219057319_0	122-136	LPNPSLHLTDTLMQL	0.043
>LocNES219057319_0	123-137	PNPSLHLTDTLMQLL	0.213
>LocNES219057319_0	130-144	TDTLMQLLDCCFLTL	0.658
>LocNES219057319_0	146-160	CSGQIVLVSTSVEQL	0.006
>LocNES219057319_0	262-276	RRMAHLATIDDRLRM	0.092
>LocNES219057319_0	425-439	IGLVAGYMKDEVRNL	0.064

>LocNES219057319_0	430-444	GYMKDEVRNLSPFCF	0.013
>LocNES219057319_0	433-447	KDEVRNLSPFCFMHL	0.053
>LocNES219057319_0	446-460	HLDDVRWVIVALRQM	0.044
>LocNES219057319_0	478-492	NGRFIYLHTKGFLEV	0.022
>LocNES219057319_0	514-528	GRQKVQEMKEKFSTI	0.080
>LocNES219057319_0	542-556	LPASQAPQQLERIVL	0.178
>LocNES219057319_0	611-625	APSSVKSSISKSVSV	0.034
>LocNES219057319_0	614-628	SVKSSISKSVSVVNV	0.009

NetNES

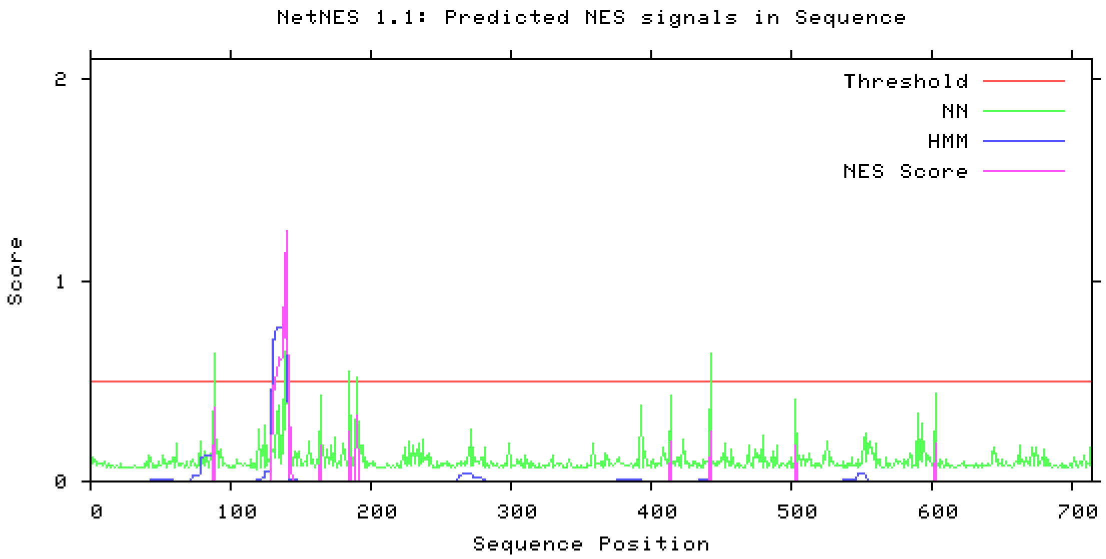

GCE 959aa isoform

```
>tr|Q9VXW7|Q9VXW7_DROME Germ cell-expressed bHLH-PAS, isoform C
OS=Drosophila melanogaster OX=7227 GN=gce PE=1 SV=2
MEGASRSRNSSTSHSQGRGQDIEDLKQDIPYFDEPPALDADLLVLGKSECQLDELAWDRD
ADGDADAPLETAPAVDLEEDNYPDENESSVLGSDYAPSGSGSGANSFYQSPTPSATGSGC
DLMLRPPSNSMYHFNYRSPGSPMPVAPGVTNSRGLHPYAHSPAHGNPPGFYPNMWYPNAP
YGSAGAAGSAGGAVSGGRYMGYGPGGVPGGTNSGPGAGPGAMQAAYPGHSAHMHALHHQY
PQPHPHAHHPQHPHHSPHPHHPHPHETMMEMFQLSNSGREARNRAEKNRRDKLNGSIQEL
STMVPHVAESPRRVDKTAVLRFAAHALRLKHAFGNSLMQQRPQITDTLMDMLDSFFLTLT
CHGHILLISASIEQHLGHCQSDLYGQSIMQITHPEDQNMLKQQLIPTELENLFDAHGDSD
AEGEPRQRSKAEEDAIDRKLREDRRSFRVRLARAGPRSEPTAYEVVKIDGCFRRSDEAPR
GVRSNHFSSNLQLIRRTRGRDDVIPLHTISGNDIILTGCARIIRPPKIASRLIDANTLEY
KTRHLIDGRIIDCDQRIGIVAGYMTDEVRNLSPFTFMHNDDVRWVIVALRQMYDCNSSYG
ESTYRLFTRNGNIIYLQSKGYLEIDKETNKVHSFVCVNTLLGEEEGKRRVQEMKKKFSVI
INTQIPQSTIDVPASEHPALLEKAVLRLIQNLQKSGENGGHDDGDEDDDAQDGDDDEEDD
DDDQDDGARSMSEFGDPYGSHHGRSHHGSSALSSHGHGNAKTPPLALVPPEASSVKSAIT
KSISVVNVTAAKHLRGIHASTAVKSPSSLGSCTCSDSHSPCDFCQGAPTTDLQAVGSNLK
RGSTAHVETEEKLSKRRFIPSTEIEHVLHTSLDQIGRNLTQQLNVARNLREQSQRYELPH
ANQRFDEIMQEHQKQSELYVNIKSEYEVQLQHKASTRKSSDSDRNQEQPPPPLQEDDQD
```

cNLS Mapper Result 2.0

Predicted NLSs in query sequence

```
MEGASRSRNSSTSHSQGRGQDIEDLKQDIPYFDEPPALDADLLVLGKSEC  50
QLDELAWDRDADGDADAPLETAPAVDLEEDNYPDENESSVLGSDYAPSGS 100
GSGANSFYQSPTPSATGSGCDLMLRPPSNSMYHFNYRSPGSPMPVAPGVT 150
NSRGLHPYAHSPAHGNPPGFYPNMWYPNAPYGSAGAAGSAGGAVSGGRYM 200
GYGPGGVPGGTNSGPGAGPGAMQAAYPGHSAHMHALHHQYPQPHPHAHHP 250
QHPHHSPHPHHPHPHETMMEMFQLSNSGREARNRAEKNRRDKLNGSIQEL 300
STMVPHVAESPRRVDKTAVLRFAAHALRLKHAFGNSLMQQRPQITDTLMD 350
MLDSFFLTLTCHGHILLISASIEQHLGHCQSDLYGQSIMQITHPEDQNML 400
KQQLIPTELENLFDAHGDSDAEGEPRQRSKAEEDAIDRKLREDRRSFRVR 450
LARAGPRSEPTAYEVVKIDGCFRRSDEAPRGVRSNHFSSNLQLIRRTRGR 500
DDVIPLHTISGNDIILTGCARIIRPPKIASRLIDANTLEYKTRHLIDGRI 550
IDCDQRIGIVAGYMTDEVRNLSPFTFMHNDDVRWVIVALRQMYDCNSSYG 600
ESTYRLFTRNGNIIYLQSKGYLEIDKETNKVHSFVCVNTLLGEEEGKRRV 650
QEMKKKFSVIINTQIPQSTIDVPASEHPALLEKAVLRLIQNLQKSGENGG 700
HDDGDEDDDAQDGDDDEEDDDDDQDDGARSMSEFGDPYGSHHGRSHHGSS 750
ALSSHGHGNAKTPPLALVPPEASSVKSAITKSISVVNVTAAKHLRGIHAS 800
TAVKSPSSLGSCTCSDSHSPCDFCQGAPTTDLQAVGSNLKRGSTAHVETE 850
EKLSKRRFIPSTEIEHVLHTSLDQIGRNLTQQLNVARNLREQSQRYELPH 900
ANQRFDEIMQEHQKQSELYVNIKSEYEVQLQHKASTRKSSDSDRNQEQPP 950
PPLQEDDQD                                          959
```

Predicted monopartite NLS

Pos.	Sequence	Score
851	EKLSKRRFIPS	2

Predicted bipartite NLS

Pos.	Sequence	Score
150	TNSRGLHPYAHSPAHGNPPGFYPNMWYPNAP	2
279	REARNRAEKNRRDKLNGSIQELSTMVPHVAESPRR	3.3
282	RNRAEKNRRDKLNGSIQELSTMVPHVAESPRRVDKT	2.8
284	RAEKNRRDKLNGSIQELSTMVPHVAESPRRVDKTA	2.2
328	RLKHAFGNSLMQQRPQITDTLMDMLDSFF	2
424	EPRQRSKAEEDAIDRKLREDRRSFRVRLARA	3
469	DGCFRRSDEAPRGVRSNHFSSNLQLIRRTRG	3.1
494	IRRTRGRDDVIPLHTISGNDIILTGCARII	2.4
521	RIIRPPKIASRLIDANTLEYKTRHLIDGRIIDCD	3.2
623	EIDKETNKVHSFVCVNTLLGEEEGKRRVQE	2.1
625	DKETNKVHSFVCVNTLLGEEEGKRRVQEMKK	2.7
648	RRVQEMKKKFSVIINTQIPQSTIDVPASEHPAL	2.2
801	TAVKSPSSLGSCTCSDSHSPCDFCQGAPTTD	2.3
838	NLKRGSTAHVETEEKLSKR	2
838	NLKRGSTAHVETEEKLSKRRFIP	12.5
838	NLKRGSTAHVETEEKLSKRRFIPSTEIEH	3
850	EEKLSKRRFIPSTEIEHVLHTSLDQIGRNLT	3.8
851	EKLSKRRFIPSTEIEHVLHTSLDQIGRNLTQ	3.1
911	EHQKQSELYVNIKSEYEVQLQHKASTRKSSD	2.5

NLStradamus

278 - GREARNRAEKNRRDKLN - 294

426 - RQRSKAEEDAIDRKLREDRRSFRVRLARAGPRS - 458

647 - KRRVQEMKKK - 656

NucPred

The NucPred score for your sequence is 0.87 (see score help below)

```
  1  MEGASRSRNSSTSHSQGRGQDIEDLKQDIPYFDEPPALDADLLVLGKSEC   50
 51  QLDELAWDRDADGDADAPLETAPAVDLEEDNYPDENESSVLGSDYAPSGS  100
101  GSGANSFYQSPTPSATGSGCDLMLRPPSNSMYHFNYRSPGSPMPVAPGVT  150
151  NSRGLHPYAHSPAHGNPPGFYPNMWYPNAPYGSAGAAGSAGGAVSGGRYM  200
201  GYGPGGVPGGTNSGPGAGPGAMQAAYPGHSAHMHALHHQYPQPHPHAHHP  250
251  QHPHHSPHPHHPHPHETMMEMFQLSNSGREARNRAEKNRRDKLNGSIQEL  300
301  STMVPHVAESPRRVDKTAVLRFAAHALRLKHAFGNSLMQQRPQITDTLMD  350
351  MLDSFFLTLTCHGHILLISASIEQHLGHCQSDLYGQSIMQITHPEDQNML  400
401  KQQLIPTELENLFDAHGDSDAEGEPRQRSKAEEDAIDRKLREDRRSFRVR  450
451  LARAGPRSEPTAYEVVKIDGCFRRSDEAPRGVRSNHFSSNLQLIRRTRGR  500
501  DDVIPLHTISGNDIILTGCARIIRPPKIASRLIDANTLEYKTRHLIDGRI  550
551  IDCDQRIGIVAGYMTDEVRNLSPFTFMHNDDVRWVIVALRQMYDCNSSYG  600
601  ESTYRLFTRNGNIIYLQSKGYLEIDKETNKVHSFVCVNTLLGEEEGKRRV  650
651  QEMKKKFSVIINTQIPQSTIDVPASEHPALLEKAVLRLIQNLQKSGENGG  700
701  HDDGDEDDDAQDGDDDEEDDDDDQDDGARSMSEFGDPYGSHHGRSHHGSS  750
751  ALSSHGHGNAKTPPLALVPPEASSVKSAITKSISVVNVTAAKHLRGIHAS  800
801  TAVKSPSSLGSCTCSDSHSPCDFCQGAPTTDLQAVGSNLKRGSTAHVETE  850
851  EKLSKRRFIPSTEIEHVLHTSLDQIGRNLTQQLNVARNLREQSQRYELPH  900
901  ANQRFDEIMQEHQKQSELYVNIKSEYEVQLQHKASTRKSSDSDRNQEQPP  950
951  PPLQEDDQD                                           959
```

Positively and negatively influencing subsequences are coloured according to the following scale:

(non-nuclear) negative positive (nuclear)

PSORT II

NUCDISC: discrimination of nuclear localization signals

```
      pat4: none
      pat7: none
      bipartite: KRGSTAHVETEEKLSKR at  840
      content of basic residues:  10.0%
      NLS Score:  0.02
```

SeqNLS

Prediction result (The predicted NLS(s) are underlined)

: MEGASRSRNSS**TSHSQGRGQDIEDL**KQDIPYFDEPPAL**DADLLVLGKSECQLDELA**WDRD

Definition of different colors in predictions

score range	color
0.1-0.3	
0.3-0.5	
0.5-0.7	
0.7-0.8	
0.8-0.86	
0.86-0.89	
>0.89	

The predicted NLS(s) (score cutoff = 0.3)

Protein ID	Predicted NLS	Start	Stop	The highest score of matches within the prediction
	DADLLVLGKSECQLDELA	39	56	0.399
	TSHSQGRGQDIEDL	12	25	0.346

ELM

TRG_NLS_Bipartite_1 KRGSTAHVETEEKLSKRRFI 840-859 Probability 2.588e-04
Bipartite variant of the classical basically charged NLS.
TRG_NLS_MonoExtC_3 GKRRVQE 646-652 Probability 7.252e-04
Monopartite variant of the classical basically charged NLS. C-extended version.
TRG_NLS_MonoExtN_4 KLSKRRFI 852-859 Probability 1.276e-03
Monopartite variant of the classical basically charged NLS. N-extended version.

No NES positive result

NES Finder

==============================
567 EVRNLSPFTF 2-2-1
650 VQEMKKKFSV 2-3-1

LocNES

Protein Name	Position	Sequence	Score
>LocNES1666756443_0	22-36	RGQDIEDLKQDIPYF	0.073
>LocNES1666756443_0	35-49	YFDEPPALDADLLVL	0.130
>LocNES1666756443_0	67-81	GDADAPLETAPAVDL	0.025
>LocNES1666756443_0	264-278	HHPHPHETMMEMFQL	0.084
>LocNES1666756443_0	297-311	LNGSIQELSTMVPHV	0.006

>LocNES1666756443_0	312-326	AESPRRVDKTAVLRF	0.306
>LocNES1666756443_0	319-333	DKTAVLRFAAHALRL	0.509
>LocNES1666756443_0	345-359	RPQITDTLMDMLDSF	0.389
>LocNES1666756443_0	347-361	QITDTLMDMLDSFFL	0.533
>LocNES1666756443_0	349-363	TDTLMDMLDSFFLTL	0.495
>LocNES1666756443_0	561-575	IGIVAGYMTDEVRNL	0.038
>LocNES1666756443_0	566-580	GYMTDEVRNLSPFTF	0.041
>LocNES1666756443_0	582-596	HNDDVRWVIVALRQM	0.044
>LocNES1666756443_0	627-641	EIDKETNKVHSFVCV	0.016
>LocNES1666756443_0	649-663	EGKRRVQEMKKKFSV	0.043
>LocNES1666756443_0	650-664	GKRRVQEMKKKFSVI	0.026
>LocNES1666756443_0	775-789	EASSVKSAITKSISV	0.011
>LocNES1666756443_0	778-792	SVKSAITKSISVVNV	0.016
>LocNES1666756443_0	875-889	SLDQIGRNLTQQLNV	0.038
>LocNES1666756443_0	898-912	QRYELPHANQRFDEI	0.015

NetNES 1.1 Server - prediction results

>Sequence - NetNES 1.1 prediction

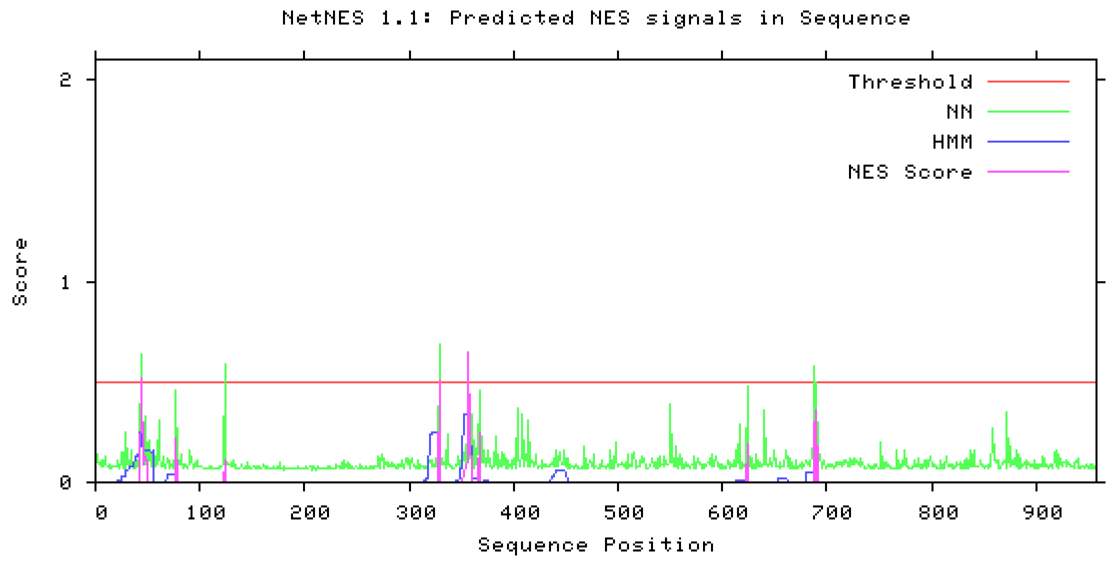

SIMA

>sp|Q24167|SIMA_DROME Protein similar OS=Drosophila melanogaster OX=7227 GN=sima PE=1 SV=2
MVSLIDTIEAAAEKQKQSQAVVTNTSASSSSCSSSFSSSPPSSSVGSPSPGAPKTNLTAS

```
GKPKEKRRNNEKRKEKSRDAARCRRSKETEIFMELSAALPLKTDDVNQLDKASVMRITIA
FLKIREMLQFVPSLRDCNDDIKQDIETAEDQQEVKPKLEVGTEDWLNGAEARELLKQTMD
GFLLVLSHEGDITYVSENVVEYLGITKIDTLGQQIWEYSHQCDHAEIKEALSLKRELAQK
VKDEPQQNSGVSTHHRDLFVRLKCTLTSRGRSINIKSASYKVIHITGHLVVNAKGERLLM
AIGRPIPHPSNIEIPLGTSTFLTKHSLDMRFTYVDDKMHDLLGYSPKDLLDTSLFSCQHG
ADSERLMATFKSVLSKGQGETSRYRFLGKYGGYCWILSQATIVYDKLKPQSVVCVNYVIS
NLENKHEIYSLAQQTAASEQKEQHHQAAETEKEPEKAADPEIIAQETKETVNTPIHTSEL
QAKPLQLESEKAEKTIEETKTIATIPPVTATSTADQIKQLPESNPYKQILQAELLIKREN
HSPGPRTITAQLLSGSSSGLRPEEKRPKSVTASVLRPSPAPPLTPPPTAVLCKKTPLGVE
PNLPPTTTATAAIISSSNQQLQIAQQTQLQNPQQPAQDMSKGFCSLFADDGRGLTMLKEE
PDDLSHHLASTNCIQLDEMTPFSDMLVGLMGTCLLPEDINSLDSTTCSTTASGQHYQSPS
SSSTSAPSNTSSSNNSYANSPLSPLTPNSTATASNPSHQQQQQHHNQQQQQQQQQQHHPQ
HHDNSNSSSNIDPLFNYREESNDTSCSQHLHSPSITSKSPEDSSLPSLCSPNSLTQEDDF
SFEAFAMRAPYIPIDDDMPLLTETDLMWCPPEDLQTMVPKEIDAIQQQLQQLQQQHHQQY
AGNTGYQQQQQQPQLQQQHFSNSLCSSPASTVSSLSPSPVQQHHQQQQAAVFTSDSSELA
ALLCGSGNGTLSILAGSGVTVAEECNERLQQHQQQQQQTSGNEFRTFQQLQQELQLQEEQ
QQRQQQQQQQQQQQQQQQLLSLNIECKKEKYDVQMGGSLCHPMEDAFENDYSKDSANLDC
WDLIQMQVVDTEPVSPNAASPTPCKVSAIQLLQQQQQLQQQQQQQQNIILNAVPLITIQN
NKELMQQQQQQQQQQQQEQLQQPAIKLLNGASIAPVNTKATIRLVESKPPTTTQSRMAKV
NLVPQQQQHGNKRHLNSATGAGNPVESKRLKSGTLCLDVQSPQLLQQLIGKDPAQQQTQA
AKRAGSERWQLSAESKQQKQQQQQSNSVLKNLLVSGRDDDDSEAMIIDEDNSLVQPIPLG
KYGLPLHCHTSTSSVLRDYHNNPLISGTNFQLSPVFGGSDSSGGDGETGSVVSLDDSVPP
GLTACDTDASSDSGIDENSLMDGASGSPRKRLSSTSNSTNQAESAPPALDVETPVTQKSV
EEEFEGGGSGSNAPSRKTSISFLDSSNPLLHTPAMMDLVNDDYIMGEGGFEFSDNQLEQV
LGWPEIA
```

cNLS Mapper Result 2.0

Predicted NLSs in query sequence

```
MVSLIDTIEAAAEKQKQSQAVVTNTSASSSSCSSSFSSSPPSSSVGSPSP  50
GAPKTNLTASGKPKEKRRNNEKRKEKSRDAARCRRSKETEIFMELSAALP  100
LKTDDVNQLDKASVMRITIAFLKIREMLQFVPSLRDCNDDIKQDIETAED  150
QQEVKPKLEVGTEDWLNGAEARELLKQTMDGFLLVLSHEGDITYVSENVV  200
EYLGITKIDTLGQQIWEYSHQCDHAEIKEALSLKRELAQKVKDEPQQNSG  250
VSTHHRDLFVRLKCTLTSRGRSINIKSASYKVIHITGHLVVNAKGERLLM  300
AIGRPIPHPSNIEIPLGTSTFLTKHSLDMRFTYVDDKMHDLLGYSPKDLL  350
DTSLFSCQHGADSERLMATFKSVLSKGQGETSRYRFLGKYGGYCWILSQA  400
TIVYDKLKPQSVVCVNYVISNLENKHEIYSLAQQTAASEQKEQHHQAAET  450
EKEPEKAADPEIIAQETKETVNTPIHTSELQAKPLQLESEKAEKTIEETK  500
TIATIPPVTATSTADQIKQLPESNPYKQILQAELLIKRENHSPGPRTITA  550
QLLSGSSSGLRPEEKRPKSVTASVLRPSPAPPLTPPPTAVLCKKTPLGVE  600
PNLPPTTTATAAIISSSNQQLQIAQQTQLQNPQQPAQDMSKGFCSLFADD  650
GRGLTMLKEEPDDLSHHLASTNCIQLDEMTPFSDMLVGLMGTCLLPEDIN  700
SLDSTTCSTTASGQHYQSPSSSSTSAPSNTSSSNNSYANSPLSPLTPNST  750
ATASNPSHQQQQQHHNQQQQQQQQQQHHPQHHDNSNSSSNIDPLFNYREE  800
SNDTSCSQHLHSPSITSKSPEDSSLPSLCSPNSLTQEDDFSFEAFAMRAP  850
YIPIDDDMPLLTETDLMWCPPEDLQTMVPKEIDAIQQQLQQLQQQHHQQY  900
AGNTGYQQQQQQPQLQQQHFSNSLCSSPASTVSSLSPSPVQQHHQQQQAA  950
VFTSDSSELAALLCGSGNGTLSILAGSGVTVAEECNERLQQHQQQQQQTS  1000
GNEFRTFQQLQQELQLQEEQQQRQQQQQQQQQQQQQQQLLSLNIECKKEK  1050
YDVQMGGSLCHPMEDAFENDYSKDSANLDCWDLIQMQVVDTEPVSPNAAS  1100
PTPCKVSAIQLLQQQQQLQQQQQQQQNIILNAVPLITIQNNKELMQQQQQ  1150
QQQQQQQEQLQQPAIKLLNGASIAPVNTKATIRLVESKPPTTTQSRMAKV  1200
```

```
NLVPQQQQHGNKRHLNSATGAGNPVESKRLKSGTLCLDVQSPQLLQQLIG 1250
KDPAQQQTQAAKRAGSERWQLSAESKQQKQQQQQSNSVLKNLLVSGRDDD 1300
DSEAMIIDEDNSLVQPIPLGKYGLPLHCHTSTSSVLRDYHNNPLISGTNF 1350
QLSPVFGGSDSSGGDGETGSVVSLDDSVPPGLTACDTDASSDSGIDENSL 1400
MDGASGSPRKRLSSTSNSTNQAESAPPALDVETPVTQKSVEEEFEGGGSG 1450
SNAPSRKTSISFLDSSNPLLHTPAMMDLVNDDYIMGEGGFEFSDNQLEQV 1500
LGWPEIA                                            1507
```

Predicted monopartite NLS

Pos.	Sequence	Score
63	PKEKRRNNEK	5
1409	RKRLSSTSN	3

Predicted bipartite NLS

Pos.	Sequence	Score
51	GAPKTNLTASGKPKEKRRNNEKRKEKSRDA	2.1
61	GKPKEKRRNNEKRKEKSRDAARCRRSKET	2.6
62	KPKEKRRNNEKRKEKSRDAARCRRSKETEI	2.9
67	RRNNEKRKEKSRDAARCRRSKETEIFMELSAA	2.6
67	RRNNEKRKEKSRDAARCRRSKETEIFMELSAALP	2.1
132	PSLRDCNDDIKQDIETAEDQQEVKPKLEVGT	2.6
150	DQQEVKPKLEVGTEDWLNGAEARELLKQTMDG	2.9
229	EALSLKRELAQKVKDEPQQNSGVSTHHRDLFV	2.7
235	RELAQKVKDEPQQNSGVSTHHRDLFVRLKCTLT	2.7
281	KVIHITGHLVVNAKGERLLMAIGRPIP	2.2
321	FLTKHSLDMRFTYVDDKMHDLLGYSPKDLLD	2.4
347	KDLLDTSLFSCQHGADSERLMATFKSV	3.1
383	RYRFLGKYGGYCWILSQATIVYDKLKPQSVVCV	3
439	EQKEQHHQAAETEKEPEKAADPEIIAQETK	2
522	ESNPYKQILQAELLIKRENHSPGPRTITAQLLS	2.9
538	RENHSPGPRTITAQLLSGSSSGLRPEEKRPKSVTAS	3.2
561	RPEEKRPKSVTASVLRPSPAPPLTPPPTAVL	3.3
561	RPEEKRPKSVTASVLRPSPAPPLTPPPTAVLCKKT	2.3
1044	IECKKEKYDVQMGGSLCHPMEDAFENDYSKDS	2.3
1102	TPCKVSAIQLLQQQQQLQQQQQQQQNIILN	2.8
1138	IQNNKELMQQQQQQQQQQQQEQLQQPAIKLL	2.3
1163	PAIKLLNGASIAPVNTKATIRLVESKPPTTT	2.1
1183	RLVESKPPTTTQSRMAKVNLVPQQQQHGNKRH	2
1212	KRHLNSATGAGNPVESKRLKS	6.4
1212	KRHLNSATGAGNPVESKRLKSGTL	4.1
1212	KRHLNSATGAGNPVESKRLKSGTLCLD	3.8
1226	ESKRLKSGTLCLDVQSPQLLQQLIGKDPA	2.4
1226	ESKRLKSGTLCLDVQSPQLLQQLIGKDPA	2.1
1262	KRAGSERWQLSAESKQQKQQQ	2.2
1262	KRAGSERWQLSAESKQQKQQQQQSNSV	2.9
1263	RAGSERWQLSAESKQQKQQQQQSNSVLKNLLVS	2.7
1288	VLKNLLVSGRDDDDSEAMIIDEDNSLVQPI	3.3
1317	IPLGKYGLPLHCHTSTSSVLRDYHNNPLISGT	3.2
1408	PRKRLSSTSNSTNQAESAPPALDVETPVT	2.9
1454	PSRKTSISFLDSSNPLLHTPAMMDLVNDDYI	2.3

NLStradamus cutoff 0,1

Posterior @ 0.1	46 - GSPSPGAPKTNLTASGKPKEKRRNNEKRKEKSRDAARCRRSKETEI - 91
	545 - PR - 546
	554 - SGSSSGLRPEEKRPKSVTASVLRPSPAP - 581

NucPred

The NucPred score for your sequence is 0.94 (see score help below)

```
   1  MVSLIDTIEAAAEKQKQSQAVVTNTSASSSSCSSSFSSSPPSSSVGSPSP    50
  51  GAPKTNLTASGKPKEKRRNNEKRKEKSRDAARCRRSKETEIFMELSAALP   100
 101  LKTDDVNQLDKASVMRITIAFLKIREMLQFVPSLRDCNDDIKQDIETAED   150
 151  QQEVKPKLEVGTEDWLNGAEARELLKQTMDGFLLVLSHEGDITYVSENVV   200
 201  EYLGITKIDTLGQQIWEYSHQCDHAEIKEALSLKRELAQKVKDEPQQNSG   250
 251  VSTHHRDLFVRLKCTLTSRGRSINIKSASYKVIHITGHLVVNAKGERLLM   300
 301  AIGRPIPHPSNIEIPLGTSTFLTKHSLDMRFTYVDDKMHDLLGYSPKDLL   350
 351  DTSLFSCQHGADSERLMATFKSVLSKGQGETSRYRFLGKYGGYCWILSQA   400
 401  TIVYDKLKPQSVVCVNYVISNLENKHEIYSLAQQTAASEQKEQHHQAAET   450
 451  EKEPEKAADPEIIAQETKETVNTPIHTSELQAKPLQLESEKAEKTIEETK   500
 501  TIATIPPVTATSTADQIKQLPESNPYKQILQAELLIKRENHSPGPRTITA   550
 551  QLLSGSSSGLRPEEKRPKSVTASVLRPSPAPPLTPPPTAVLCKKTPLGVE   600
 601  PNLPPTTTATAAIISSSNQQLQIAQQTQLQNPQQPAQDMSKGFCSLFADD   650
 651  GRGLTMLKEEPDDLSHHLASTNCIQLDEMTPFSDMLVGLMGTCLLPEDIN   700
 701  SLDSTTCSTTASGQHYQSPSSSSTSAPSNTSSSNNSYANSPLSPLTPNST   750
 751  ATASNPSHQQQQQHHNQQQQQQQQQQHHPQHHDNSNSSSNIDPLFNYREE   800
 801  SNDTSCSQHLHSPSITSKSPEDSSLPSLCSPNSLTQEDDFSFEAFAMRAP   850
 851  YIPIDDDMPLLTETDLMWCPPEDLQTMVPKEIDAIQQQLQQLQQQHHQQY   900
 901  AGNTGYQQQQQQPQLQQQHFSNSLCSSPASTVSSLSPSPVQQHHQQQQAA   950
 951  VFTSDSSELAALLCGSGNGTLSILAGSGVTVAEECNERLQQHQQQQQQTS  1000
1001  GNEFRTFQQLQQELQLQEEQQQRQQQQQQQQQQQQQQLLSLNIECKKEK  1050
1051  YDVQMGGSLCHPMEDAFENDYSKDSANLDCWDLIQMQVVDTEPVSPNAAS  1100
1101  PTPCKVSAIQLLQQQQQLQQQQQQQQNIILNAVPLITIQNNKELMQQQQQ  1150
1151  QQQQQQQEQLQQPAIKLLNGASIAPVNTKATIRLVESKPPTTTQSRMAKV  1200
1201  NLVPQQQQHGNKRHLNSATGAGNPVESKRLKSGTLCLDVQSPQLLQQLIG  1250
1251  KDPAQQQTQAAKRAGSERWQLSAESKQQKQQQQQSNSVLKNLLVSGRDDD  1300
1301  DSEAMIIDEDNSLVQPIPLGKYGLPLHCHTSTSSVLRDYHNNPLISGTNF  1350
1351  QLSPVFGGSDSSGGDGETGSVVSLDDSVPPGLTACDTDASSDSGIDENSL  1400
1401  MDGASGSPRKRLSSTSNSTNQAESAPPALDVETPVTQKSVEEEFEGGGSG  1450
1451  SNAPSRKTSISFLDSSNPLLHTPAMMDLVNDDYIMGEGGFEFSDNQLEQV  1500
1501  LGWPEIA                                             1507
```

Positively and negatively influencing subsequences are coloured according to the following scale:

(non-nuclear) negative |||||||||||||||||||||||||||||||||||||||||||||||||||||||||||||||||||||||||||||||||||||||||||||||||||| positive (nuclear)

PSORT II

[NUCDISC: discrimination of nuclear localization signals]

```
      pat4: KRPK (4) at  565
      pat4: PRKR (4) at 1408
      pat7: PKEKRRN- (5) at   63
      pat7: PEEKRPK (3) at  562
      pat7: PRKRLSS (5) at 1408
      bipartite: KRKEKSRDAARCRRSKE at   72
      content of basic residues:   8.0%
      NLS Score:  1.64
```

Seq NLS

Prediction result (The predicted NLS(s) are underlined)

: MVSLIDTIEAAAEKQKQSQAVVTNTSASSSSCSSSFSSSPPSSSVGSPSPGAPKTNLTAS

Definition of different colors in predictions

score range	color
0.1-0.3	
0.3-0.5	
0.5-0.7	
0.7-0.8	
0.8-0.86	
0.86-0.89	
>0.89	

The predicted NLS(s) (score cutoff = 0.3)

Protein ID	Predicted NLS	Start	Stop	The highest score of matches within the prediction
	LIDTIEAAAEKQKQSQAVVTNTSASSSSC	4	32	0.902
	SPSPGAPKTNLTA	47	59	0.605

ELM

TRG_NLS_Bipartite_1 KRHLNSATGAGNPVESKRLKS 1212-1232 2.588e-04

Bipartite variant of the classical basically charged NLS.

TRG_NLS_MonoExtN_4 PRKRLS 1408-1413 1.276e-03

Monopartite variant of the classical basically charged NLS. N-extended version.

TRG_NES_CRM1_1 90-105; 173-189 7.626e-04

Some proteins re-exported from the nucleus contain a Leucine-rich nuclear export signal (NES) binding to the CRM1 exportin protein.

NES Finder

Position	NES	Type
92	FMELSAALPL	2-3-1
115	MRITIAFLKI	3-2-1
121	FLKIREMLQF	2-3-1
1007	FQQLQQELQL	2-3-1
1129	ILNAVPLITI	3-2-1

LocNES

Protein Name	Position	Sequence	Score
>LocNES1701850396_0	91-105	KETEIFMELSAALPL	0.254
>LocNES1701850396_0	107-121	TDDVNQLDKASVMRI	0.142
>LocNES1701850396_0	109-123	DVNQLDKASVMRITI	0.105
>LocNES1701850396_0	111-125	NQLDKASVMRITIAF	0.149
>LocNES1701850396_0	112-126	QLDKASVMRITIAFL	0.163
>LocNES1701850396_0	114-128	DKASVMRITIAFLKI	0.318
>LocNES1701850396_0	117-131	SVMRITIAFLKIREM	0.031
>LocNES1701850396_0	120-134	RITIAFLKIREMLQF	0.139
>LocNES1701850396_0	121-135	ITIAFLKIREMLQFV	0.062
>LocNES1701850396_0	124-138	AFLKIREMLQFVPSL	0.036
>LocNES1701850396_0	174-188	EARELLKQTMDGFLL	0.098
>LocNES1701850396_0	175-189	ARELLKQTMDGFLLV	0.398
>LocNES1701850396_0	195-209	DITYVSENVVEYLGI	0.028
>LocNES1701850396_0	275-289	RSINIKSASYKVIHI	0.003
>LocNES1701850396_0	281-295	SASYKVIHITGHLVV	0.010
>LocNES1701850396_0	331-345	LDMRFTYVDDKMHDL	0.058
>LocNES1701850396_0	412-426	KPQSVVCVNYVISNL	0.040
>LocNES1701850396_0	477-491	TPIHTSELQAKPLQL	0.437
>LocNES1701850396_0	636-650	PQQPAQDMSKGFCSL	0.226

>LocNES1701850396_0	666-680	DDLSHHLASTNCIQL	0.032
>LocNES1701850396_0	677-691	CIQLDEMTPFSDMLV	0.086
>LocNES1701850396_0	679-693	QLDEMTPFSDMLVGL	0.125
>LocNES1701850396_0	692-706	GLMGTCLLPEDINSL	0.127
>LocNES1701850396_0	837-851	SLTQEDDFSFEAFAM	0.036
>LocNES1701850396_0	844-858	FSFEAFAMRAPYIPI	0.015
>LocNES1701850396_0	882-896	VPKEIDAIQQQLQQL	0.030
>LocNES1701850396_0	971-985	GNGTLSILAGSGVTV	0.105
>LocNES1701850396_0	1006-1020	NEFRTFQQLQQELQL	0.235
>LocNES1701850396_0	1101-1115	NAASPTPCKVSAIQL	0.019
>LocNES1701850396_0	1125-1139	QQQQQQNIILNAVPL	0.130
>LocNES1701850396_0	1128-1142	QQQNIILNAVPLITI	0.089
>LocNES1701850396_0	1192-1206	KPPTTTQSRMAKVNL	0.113
>LocNES1701850396_0	1229-1243	VESKRLKSGTLCLDV	0.066
>LocNES1701850396_0	1345-1359	NNPLISGTNFQLSPV	0.007
>LocNES1701850396_0	1468-1482	DSSNPLLHTPAMMDL	0.037
>LocNES1701850396_0	1469-1483	SSNPLLHTPAMMDLV	0.020

NetNES 1.1 Server - prediction results

>Sequence - NetNES 1.1 prediction

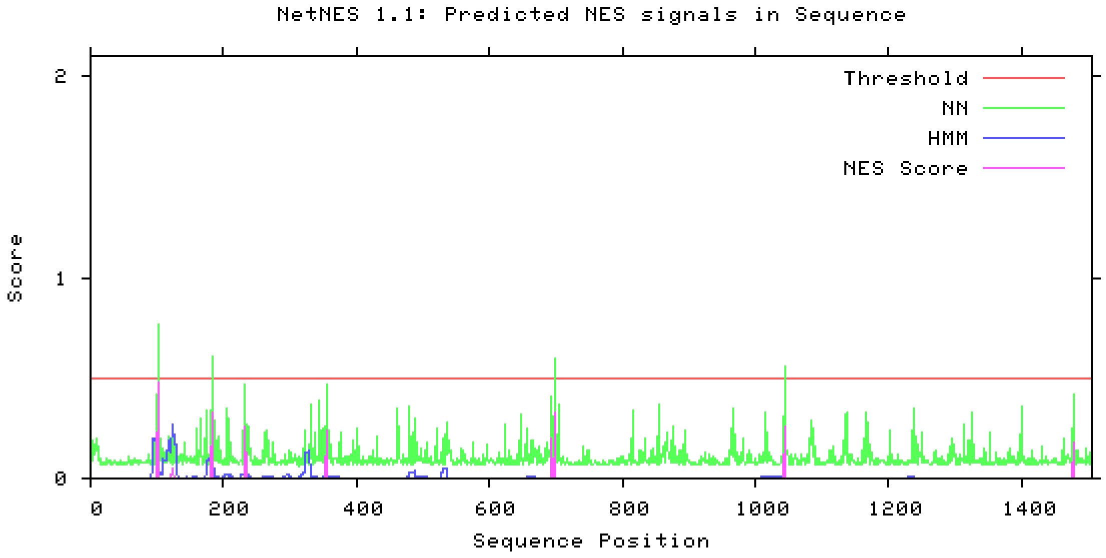

TANGO

>sp|O15945|ARNT_DROME Aryl hydrocarbon receptor nuclear translocator homolog OS=Drosophila melanogaster OX=7227 GN=tgo PE=1 SV=3
MDEANIQDKERFASRENHCEIERRRRNKMTAYITELSDMVPTCSALARKPDKLTILRMAV
AHMKALRGTGNTSSDGTYKPSFLTDQELKHLILEAADGFLFVVSCDSGRVIYVSDSVTPV
LNYTQSDWYGTSLYEHIHPDDREKIREQLSTQESQNAGRILDLKSGTVKKEGHQSSMRLS
MGARRGFICRMRVGNVNPESMVSGHLNRLKQRNSLGPSRDGTNYAVVHCTGYIKNWPPTD
MFPNMHMERDVDDMSSHCCLVAIGRLQVTSTAANDMSGSNNQSEFITRHAMDGKFTFVDQ
RVLNILGYTPTELLGKICYDFFHPEDQSHMKESFDQVLKQKGQMFSLLYRARAKNSEYVW

LRTQAYAFLNPYTDEVEYIVCTNSSGKTMHGAPLDAAAAHTPEQVQQQQQQEQHVYVQAA
PGVDYARRELTPVGSATNDGMYQTHMLAMQAPTPQQQQQQQQRPGSAQTTPVGYTYDTTH
SPYSAGGPSPLAKIPKSGTSPTPVAPNSWAALRPQQQQQQQQPVTEGYQYQQTSPARSPS
GPTYTQLSAGNGNRQQAQPGAYQAGPPPPPNAPGMWDWQQAGGHPHPPHPTAHPHHPHAH
PGGPAGAGQPQGQEFSDMLQMLDHTPTTFEDLNINMFSTPFE

cNLS Mapper Result 2.0

Predicted NLSs in query sequence	
MDEANIQDKERFASRENHCEIERRRRNKMTAYITELSDMVPTCSALARKP	50
DKLTILRMAVAHMKALRGTGNTSSDGTYKPSFLTDQELKHLILEAADGFL	100
FVVSCDSGRVIYVSDSVTPVLNYTQSDWYGTSLYEHIHPDDREKIREQLS	150
TQESQNAGRILDLKSGTVKKEGHQSSMRLSMGARRGFICRMRVGNVNPES	200
MVSGHLNRLKQRNSLGPSRDGTNYAVVHCTGYIKNWPPTDMFPNMHMERD	250
VDDMSSHCCLVAIGRLQVTSTAANDMSGSNNQSEFITRHAMDGKFTFVDQ	300
RVLNILGYTPTELLGKICYDFFHPEDQSHMKESFDQVLKQKGQMFSLLYR	350
ARAKNSEYVWLRTQAYAFLNPYTDEVEYIVCTNSSGKTMHGAPLDAAAAH	400
TPEQVQQQQQQEQHVYVQAAPGVDYARRELTPVGSATNDGMYQTHMLAMQ	450
APTPQQQQQQQQRPGSAQTTPVGYTYDTTHSPYSAGGPSPLAKIPKSGTS	500
PTPVAPNSWAALRPQQQQQQQQPVTEGYQYQQTSPARSPSGPTYTQLSAG	550
NGNRQQAQPGAYQAGPPPPPNAPGMWDWQQAGGHPHPPHPTAHPHHPHAH	600
PGGPAGAGQPQGQEFSDMLQMLDHTPTTFEDLNINMFSTPFE	642

Predicted monopartite NLS

Pos.	Sequence	Score

Predicted bipartite NLS

Pos.	Sequence	Score
15	RENHCEIERRRRNKMTAYITELSDMVPTCSALARKP	2.8
20	EIERRRRNKMTAYITELSDMVPTCSALARKPD	2.6
20	EIERRRRNKMTAYITELSDMVPTCSALARKPDK	2.6
22	ERRRRNKMTAYITELSDMVPTCSALARKPD	3.3
23	RRRRNKMTAYITELSDMVPTCSALARKPDKLT	4.6
23	RRRRNKMTAYITELSDMVPTCSALARKPDKLTI	3
75	DGTYKPSFLTDQELKHLILEAADGFLFVVSCD	2.2
85	DQELKHLILEAADGFLFVVSCDSGRVIYVSDS	2
167	TVKKEGHQSSMRLSMGARRGFICRMRVGNV	2.2
207	NRLKQRNSLGPSRDGTNYAVVHCTGYIKNWP	2.7
208	RLKQRNSLGPSRDGTNYAVVHCTGYIKNWPPT	2.6
231	GYIKNWPPTDMFPNMHMERDVDDMSSHCCL	2
288	RHAMDGKFTFVDQRVLNILGYTPTELLGKICYD	3.2
312	ELLGKICYDFFHPEDQSHMKESFDQVLKQKG	2.9
335	DQVLKQKGQMFSLLYRARAKNSEYVWLRTQAY	2.7
386	GKTMHGAPLDAAAAHTPEQVQQQQQQE	2.2
490	PLAKIPKSGTSPTPVAPNSWAALRPQQQQ	2.2
493	KIPKSGTSPTPVAPNSWAALRPQQQQQQQQP	2.1

NLStradamus cutoff 0,1

Posterior @ 0.1	11 - RFASRENHCEIERRRRNKMTAYITELSDMVPTCSALARKPDK - 52
	170 - K - 170

NucPred

The NucPred score for your sequence is 0.76 (see score help below)

```
  1   MDEANIQDKERFASRENHCEIERRRRNKMTAYITELSDMVPTCSALARKP    50
 51   DKLTILRMAVAHMKALRGTGNTSSDGTYKPSFLTDQELKHLILEAADGFL   100
101   FVVSCDSGRVIYVSDSVTPVLNYTQSDWYGTSLYEHIHPDDREKIREQLS   150
151   TQESQNAGRILDLKSGTVKKEGHQSSMRLSMGARRGFICRMRVGNVNPES   200
201   MVSGHLNRLKQRNSLGPSRDGTNYAVVHCTGYIKNWPPTDMFPNMHMERD   250
251   VDDMSSHCCLVAIGRLQVTSTAANDMSGSNNQSEFITRHAMDGKFTFVDQ   300
301   RVLNILGYTPTELLGKICYDFFHPEDQSHMKESFDQVLKQKGQMFSLLYR   350
351   ARAKNSEYVWLRTQAYAFLNPYTDEVEYIVCTNSSGKTMHGAPLDAAAAH   400
401   TPEQVQQQQQQEQHVYVQAAPGVDYARRELTPVGSATNDGMYQTHMLAMQ   450
451   APTPQQQQQQQQRPGSAQTTPVGYTYDTTHSPYSAGGPSPLAKIPKSGTS   500
501   PTPVAPNSWAALRPQQQQQQQQPVTEGYQYQQTSPARSPSGPTYTQLSAG   550
551   NGNRQQAQPGAYQAGPPPPPNAPGMWDWQQAGGHPHPPHPTAHPHHPHAH   600
601   PGGPAGAGQPQGQEFSDMLQMLDHTPTTFEDLNINMFSTPFE           642
```

PSORTII

NUCDISC: discrimination of nuclear localization signals

```
      pat4: RRRR (5) at    23
      pat7: none
      bipartite: none
      content of basic residues:   8.7%
      NLS Score: -0.16
```

SeqNLS

Prediction result (The predicted NLS(s) are underlined)

: MDEANIQDKERFASRENHCEIERRRRNKMTAYITELSDMVPTCSALARKPDKLTILRMAV

Definition of different colors in predictions

score range	color
0.1-0.3	
0.3-0.5	
0.5-0.7	
0.7-0.8	
0.8-0.86	
0.86-0.89	

>0.89	

The predicted NLS(s) (score cutoff = 0.3)

Protein ID	Predicted NLS	Start	Stop	The highest score of matches within the prediction

ELM

No NLS/NES positive result

NESFinder

```
Position        NES         Type
================================
  259        CLVAIGRLQV     2-2-1
```

LocNES

Protein Name	Position	Sequence	Score
>LocNES1465456712_0	50-64	LARKPDKLTILRMAV	0.020
>LocNES1465456712_0	53-67	KPDKLTILRMAVAHM	0.068
>LocNES1465456712_0	56-70	KLTILRMAVAHMKAL	0.076
>LocNES1465456712_0	83-97	KPSFLTDQELKHLIL	0.352
>LocNES1465456712_0	91-105	ELKHLILEAADGFLF	0.081
>LocNES1465456712_0	92-106	LKHLILEAADGFLFV	0.135
>LocNES1465456712_0	93-107	KHLILEAADGFLFVV	0.072
>LocNES1465456712_0	110-124	DSGRVIYVSDSVTPV	0.005
>LocNES1465456712_0	183-197	LSMGARRGFICRMRV	0.010
>LocNES1465456712_0	237-251	IKNWPPTDMFPNMHM	0.013
>LocNES1465456712_0	258-272	MSSHCCLVAIGRLQV	0.036
>LocNES1465456712_0	287-301	SEFITRHAMDGKFTF	0.010
>LocNES1465456712_0	295-309	MDGKFTFVDQRVLNI	0.030
>LocNES1465456712_0	423-437	AAPGVDYARRELTPV	0.017
>LocNES1465456712_0	439-453	SATNDGMYQTHMLAM	0.086
>LocNES1465456712_0	622-636	MLQMLDHTPTTFEDL	0.350
>LocNES1465456712_0	624-638	QMLDHTPTTFEDLNI	0.539

NetNES 1.1 Server - prediction results

```
>Sequence - NetNES 1.1 prediction
```

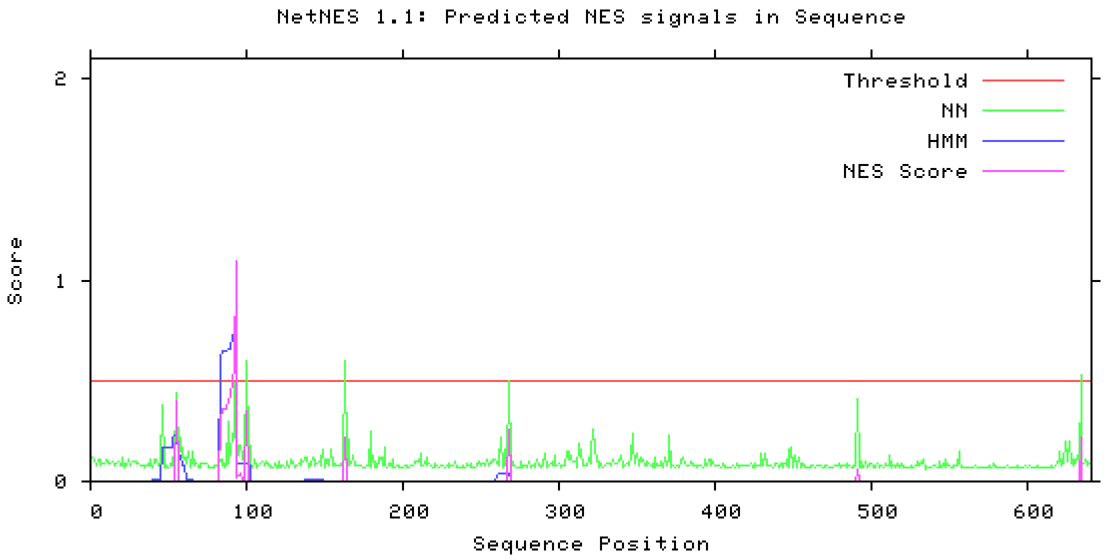

CLUSTAL 2.1 MULTIPLE SEQUENCE ALIGNMENT

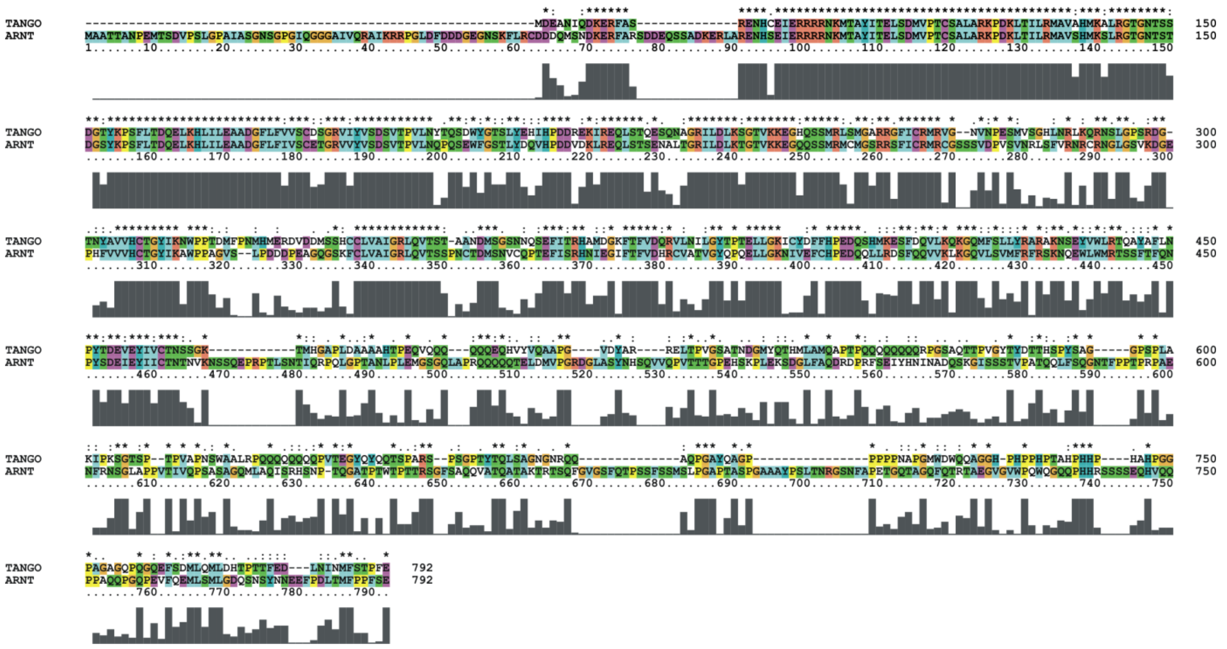

CYCLE

>sp|O61734|CYCL_DROME Protein cycle OS=Drosophila melanogaster OX=7227 GN=cyc PE=1 SV=2
MEVQEFCENMEEIEDENYDEEKSARTSDENRKQNHSEIEKRRRDKMNTYINELSSMIPMC
FAMQRKLDKLTVLRMAVQHLRGIRGSGSLHPFNGSDYRPSFLSDQELKMIILQASEGFLF
VVGCDRGRILYVSDSVSSVLNSTQADLLGQSWFDVLHPKDIGKVKEQLSSLEQCPRERLI
DAKTMLPVKTDVPQSLCRLCPGARRSFFCRMKLRTASNNQIKEESDTSSSSRSSTKRKSR
LTTGHKYRVIQCTGYLKSWTPIKDEDQDADSDEQTTNLSCLVAIGRIPPNVRNSTVPASL
DNHPNIRHVLFISRHSGEGKFLFIDQRATLVIGFLPQEILGTSFYEYFHNEDIAALMESH
KMVMQVPEKVTTQVYRFRCKDNSYIQLQSEWRAFKNPWTSEIDYIIAKNSVFL

cNLS Mapper Result 2.0

Predicted NLSs in query sequence	
MEVQEFCENMEEIEDENYDEEKSARTSDENRKQNHSEIEKRRRDKMNTYI	50
NELSSMIPMCFAMQRKLDKLTVLRMAVQHLRGIRGSGSLHPFNGSDYRPS	100
FLSDQELKMIILQASEGFLFVVGCDRGRILYVSDSVSSVLNSTQADLLGQ	150
SWFDVLHPKDIGKVKEQLSSLEQCPRERLIDAKTMLPVKTDVPQSLCRLC	200
PGARRSFFCRMKLRTASNNQIKEESDTSSSSRSSTKRKSRLTTGHKYRVI	250
QCTGYLKSWTPIKDEDQDADSDEQTTNLSCLVAIGRIPPNVRNSTVPASL	300
DNHPNIRHVLFISRHSGEGKFLFIDQRATLVIGFLPQEILGTSFYEYFHN	350
EDIAALMESHKMVMQVPEKVTTQVYRFRCKDNSYIQLQSEWRAFKNPWTS	400
EIDYIIAKNSVFL	413

Predicted monopartite NLS		
Pos.	Sequence	Score
37	EIEKRRRDKM	3

Predicted bipartite NLS		
Pos.	Sequence	Score
37	EIEKRRRDKMNTYINELSSMIPMCFAMQR	3.7
37	EIEKRRRDKMNTYINELSSMIPMCFAMQRKLDK	2.4
39	EKRRRDKMNTYINELSSMIPMCFAMQRKLD	2.5
41	RRRDKMNTYINELSSMIPMCFAMQRKLDKLT	4.8
154	DVLHPKDIGKVKEQLSSLEQCPRERLIDAKTM	2.1
160	DIGKVKEQLSSLEQCPRERLIDAKTMLPVKTD	2.3
176	RERLIDAKTMLPVKTDVPQSLCRLCPGARRSFF	2.4
176	RERLIDAKTMLPVKTDVPQSLCRLCPGARRSFFC	3.3
187	PVKTDVPQSLCRLCPGARRSFFCRMKLRT	3.1
204	RRSFFCRMKLRTASNNQIKEESDTSSSSRSSTKRKS	2.9
210	RMKLRTASNNQIKEESDTSSSSRSSTKRKSR	4.3
232	RSSTKRKSRLTTGHKYRVIQCTGYLKSWTPI	3.4
232	RSSTKRKSRLTTGHKYRVIQCTGYLKSWTPIK	3
232	RSSTKRKSRLTTGHKYRVIQCTGYLKSWTPIKDE	2.8
254	GYLKSWTPIKDEDQDADSDEQTTNLSCLVAI	2.1
259	WTPIKDEDQDADSDEQTTNLSCLVAIGRIPP	2

NLStradamus

Pre-loaded models cut-off 0.1 4 state HMM static

Posterior @ 0.1	25 - RTSDENRKQNHSEIEKRRRDKM - 46

	229 - SSSRSSTKRKSRLTTGHK - 246

NucPred

The NucPred score for your sequence is 0.90 (see score help below)

```
  1   MEVQEFCENMEEIEDENYDEEKSARTSDENRKQNHSEIEKRRRDKMNTYI    50
 51   NELSSMIPMCFAMQRKLDKLTVLRMAVQHLRGIRGSGSLHPFNGSDYRPS   100
101   FLSDQELKMIILQASEGFLFVVGCDRGRILYVSDSVSSVLNSTQADLLGQ   150
151   SWFDVLHPKDIGKVKEQLSSLEQCPRERLIDAKTMLPVKTDVPQSLCRLC   200
201   PGARRSFFCRMKLRTASNNQIKEESDTSSSSRSSTKRKSRLTTGHKYRVI   250
251   QCTGYLKSWTPIKDEDQDADSDEQTTNLSCLVAIGRIPPNVRNSTVPASL   300
301   DNHPNIRHVLFISRHSGEGKFLFIDQRATLVIGFLPQEILGTSFYEYFHN   350
351   EDIAALMESHKMVMQVPEKVTTQVYRFRCKDNSYIQLQSEWRAFKNPWTS   400
401   EIDYIIAKNSVFL                                        413
```

Positively and negatively influencing subsequences are coloured according to the following scale:

(non-nuclear) negative 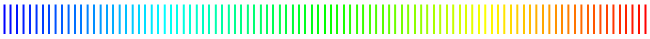 positive (nuclear)

PsortII

NUCDISC: discrimination of nuclear localization signals

```
      pat4: KRRR (5) at    40
      pat7: none
      bipartite: none
      content of basic residues:  13.6%
      NLS Score: -0.16
```

SeqNLS

Prediction result (The predicted NLS(s) are underlined)

cyc: MEVQEFCENMEEIEDENYDEEKSARTSDENRKQNHSEIEKRRRDKMNTYINELS<u>SMIPMC</u>

The predicted NLS(s) (score cutoff = 0.5)

Protein ID	Predicted NLS	Start	Stop	The highest score of matches within the prediction
cyc	SMIPMC	55	60	0.659

Prediction result (The predicted NLS(s) are underlined)

: MEVQEFCENMEEIEDENYDEEKSARTSDENRKQNHSEIEKRRRDKMNTYINELS<u>SMIPMC</u>

Definition of different colors in predictions

score range	color
0.1-0.3	
0.3-0.5	

0.5-0.7	
0.7-0.8	
0.8-0.86	
0.86-0.89	
>0.89	

The predicted NLS(s) (score cutoff = 0.3)

Protein ID	Predicted NLS	Start	Stop	The highest score of matches within the prediction
	SMIPMC	55	60	0.659

ELM

TRG_NLS_MonoExtC_3 TKRKSR 235-240 7.252e-04

Monopartite variant of the classical basically charged NLS. C-extended version.

TRG_NLS_MonoExtN_4 KRKSRL 236-241 1.276e-03

Monopartite variant of the classical basically charged NLS. N-extended version.

No NES positive result

NES Finder

Position NES Type

==============================

50 INELSSMIPM 2-3-1

63 MQRKLDKLTV 3-2-1

66 KLDKLTVLRM 2-2-1

LocNES

No NES candidates were found in your query protein

NetNES 1.1 Server - prediction results

>Sequence - NetNES 1.1 prediction

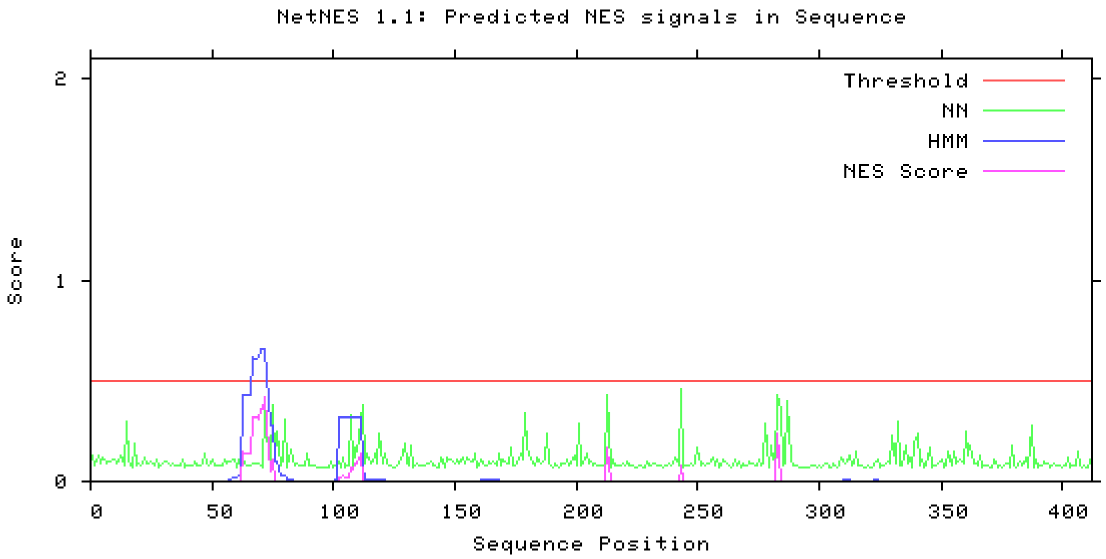

CLUSTAL 2.1 MULTIPLE SEQUENCE ALIGNMENT

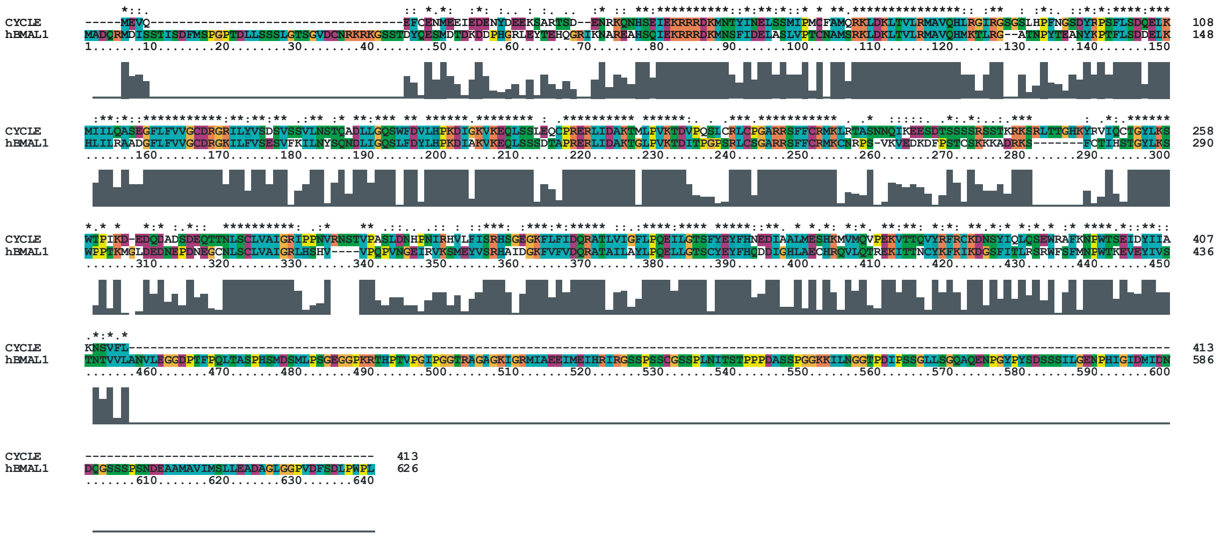

Supplement: Supplementary file 1 [file ijms-20-04746-s001.zip › SupplementaryIJMS/Supplementary materials3.pdf]
